# Efficacy and Safety of Various First-Line Therapeutic Strategies for Fetal Tachycardias: A Network Meta-Analysis and Systematic Review

**DOI:** 10.3389/fphar.2022.935455

**Published:** 2022-06-13

**Authors:** Jiangwei Qin, Zhengrong Deng, Changqing Tang, Yunfan Zhang, Ruolan Hu, Jiawen Li, Yimin Hua, Yifei Li

**Affiliations:** ^1^ Key Laboratory of Birth Defects and Related Diseases of Women and Children of MOE, Department of Pediatrics, West China Second University Hospital, Sichuan University, Chengdu, China; ^2^ Department of Pediatric Cardiology, Children’s Hospital of Soochow University, Suzhou, China

**Keywords:** fetal tachycardia, first-line therapy, digoxin, flecainide, network meta-analysis

## Abstract

**Background:** Fetal arrhythmias are common cardiac abnormalities associated with high mortality due to ventricular dysfunction and heart failure, particularly when accompanied by hydrops. Although several types of common fetal tachycardias have been relatively identified medications, such as digoxin, flecainide, and sotalol, there is no first-line drug treatment protocol established for the treatment of various types of fetal tachycardias.

**Methods:** We conducted a network meta-analysis using a Bayesian hierarchical framework to obtain a model for integrating both direct and indirect evidence. All tachycardia types (Total group), supraventricular tachycardia (SVT subgroup), atrial flutter (AF subgroup), hydrops subgroup, and non-hydrops subgroup fetuses were analyzed, and five first-line regimens were ranked according to treatment outcomes: digoxin monotherapy (D), flecainide monotherapy (F), sotalol monotherapy (S), digoxin plus flecainide combination therapy (DF), and digoxin plus sotalol combination therapy (DS). Effectiveness and safety were determined according to the cardioversion rate and intrauterine death rate.

**Results:** The pooled data indicated that DF combination therapy was always superior to D monotherapy, regardless of the tachycardia type or the presence of hydrops: Total, 2.44 (95% CrI: 1.59, 3.52); SVT, 2.77 (95% CrI: 1.59, 4.07); AF, 67.85 (95% CrI: 14.25, 168.68); hydrops, 6.03 (95% CrI: 2.54, 10.68); and non-hydrops, 5.06 (95% CrI: 1.87, 9.88). DF and F had a similar effect on control of fetal tachycardias. No significant differences were observed when comparing S, DS with D therapies across the subgroup analyses for the SVT, hydrops, and non-hydrops groups. No significant differences in mortality risks were among the various treatment regimens for the total group. And no significant differences were found in rates of intrauterine death rates at the same cardioversion amount.

**Conclusion** The flecainide monotherapy and combination of digoxin and flecainide should be considered the most superior therapeutic strategies for fetal tachycardia.

**Systematic Review Registration:** (https://www.crd.york.ac.uk/PROSPERO/display_record.php?RecordID=288997), identifier (288997).

## Introduction

Fetal arrhythmias refer to abnormalities of either the heart rate or rhythm observed in a fetus, which can be further categorized into tachycardias and bradycardias (Carvalho, 2019). Tachycardias are potentially more life-threatening than bradycardias, with an overall mortality rate of 8%–9%, and can progress to ventricular dysfunction or fetal heart failure, leading to intrauterine death. Fetal tachycardias are often misdiagnosed or unnoticed until the observation of hydrops, which may cause serious consequences such as lifetime neurological complications and deaths (Carvalho, 2019). Fetal hydrops is associated with a 35% intrauterine mortality rate, despite treatment, compared with a 0%–4% mortality rate in non-hydrops fetuses (Simpson, 2006).

The most common types of fetal tachycardias are supraventricular tachycardia (SVT) and atrial flutter (AF). SVT accounts for 66% of all fetal tachycardia cases (Crosson and Scheel, 1996), whereas AF has been reported in 25%–30% of cases (Krapp et al., 2003). Some types of tachycardias are rarely observed, including atrial ectopic tachycardia (AET) and permanent junctional reciprocating tachycardia (PJRT). In most fetuses with tachycardia, critical cardiac malformations are also considered to be significantly associated with tachycardia onset; however, congenital heart diseases (CHDs) represent major comorbidities.

Echocardiography and fetal cardiac magnetic resonance imaging (MRI) are the predominant methods used to diagnose fetal tachycardias. The optimal and timely administration of transplacental medications is necessary to regulate arrhythmias, which can develop into fetal hydrops, cardiomegaly, atrioventricular valve regurgitation, or intrauterine death (Nii et al., 2006; Carvalho et al., 2007). Patients should receive first-line treatments as soon as arrhythmias are detected or proceed to further treatments if necessary. Transplacental anti-arrhythmic therapy for fetal tachycardia was first reported in 1980 (Kerenyi et al., 1980). Since then, a series of treatment protocols have been described for fetal tachycardia, including the use of digoxin, flecainide, sotalol, amiodarone, verapamil, and propafenone. Digoxin is the most widely used first-line medication for controlling the heart rate *in utero*. Although several types of medication have been described for use as first-line treatment, well-designed multi-group comparison studies are difficult due to the limited cases of fetal tachycardiac patients. Several individual cohorts have reported on the efficacy and safety of currently available first-line treatments; therefore, a network meta-analysis can be conducted to demonstrate the differences across the various reported therapeutic strategies. Previously, two individual meta-analyses have investigated the efficacy of first-line treatments for fetal tachycardias (Alsaied et al., 2017; Hill et al., 2017). However, these studies only made pairwise comparisons among several therapeutic strategies using a typical meta-analysis approach, which may be associated with the potential existence of type II statistical errors. Moreover, recent European Society of Cardiology (ESC) guidelines indicated the need for future follow-up studies to establish specific and effective treatment protocols with minimal risks (Brugada et al., 2020). A rich body of retrospective studies that examine this issue is available, and a network meta-analysis can be applied to evaluate the efficacy and safety of various proposed first-line treatment regimens for fetal tachycardias.

## Methods

### Study Protocol

This analysis was conducted in accordance with a predetermined protocol, following the recommendations of a guideline for the reporting of systematic reviews of prognostic factor studies (Riley et al., 2019). The data collection and reporting were performed in accordance with the Preferred Reporting Items for Systematic Reviews and Meta-Analyses (PRISMA) (Moher et al., 2015) and the PRISMA Extension Statement for Reporting of Systemic Review Incorporating Network Meta-Analyses (Hutton et al., 2015).

### Search Strategy

We searched the PubMed, Web of Science, Medline, Embase, and Cochrane Library databases to identify studies comparing and evaluating the effects of various drug regimens used to regulate fetal tachycardias from database inception to July 2021, with no limits on the study design, country of origin, tachycardia type, administration route (placental, intravenous, intramuscular or other administration routes), or whether combination treatment was applied. Additional studies for inclusion were identified from among the references of relevant reviews (Alsaied et al., 2017; Hill et al., 2017; Carvalho, 2019; Yuan, 2020; Veduta et al., 2021). The search strategy was established previously, and the terms included digoxin; flecainide; sotalol; amiodarone; tachycardia(s); fetus; supraventricular tachycardia or SVT; and atrial flutter or AF. The PubMed search strategy was described as [Anti-Arrhythmia Agents (MeSH Terms)] OR [Amiodarone (MeSH Terms)] OR [Digoxin (MeSH Terms)] OR [Sotalol (MeSH Terms)] OR [Flecainide (MeSH Terms)] AND [Tachycardia (MeSH Terms)] OR [Tachycardia, Supraventricular (MeSH Terms)] OR [Atrial Flutter (MeSH Terms)] OR [Hydrops (MeSH Terms)] AND (fetus).

### Study Selection

Two authors (Jiangwei Qin and Zhengrong Deng) independently performed the literature search and performed data extraction using a standardized, pre-established form that distinguished the tachycardia types or whether hydrops existed. The full texts of the identified studies were independently assessed for inclusion by two other authors (Yifei Li and Yimin Hua), based on predetermined criteria. Each identified study was assessed for the following inclusion criteria: 1) the study population included fetuses treated with medication for sustained SVT or AF, with a diagnosis of fetal tachycardia based on the results of echocardiography or cardiac MRI; 2) the interventions were defined as anti-arrhythmia drugs administrated to the fetuses using a transplacental approach, so comparisons were made among first-line regimens: digoxin monotherapy (D), digoxin and flecainide combination therapy (DF), flecainide monotherapy (F), digoxin and sotalol combination therapy (DS), and sotalol monotherapy (S) (some drugs such as verapamil and DFS combination were discarded because they were not sufficient to produce results). As for combination therapy, to ensure that the evaluation of the efficacy of the drug is put first in this meta-analysis, considering that many doctors would promptly add drugs when a single drug is ineffective, our definition of combination drugs would be described as: if a second drug was started before the third day of treatment, it was defined as combination therapy; 3) the cardioversion success rate was assessed as the primary outcome, which was defined as the reversion to a normal sinus rhythm during or after the administration of therapeutic drugs using a transplacental approach, without recurrence or relapse until birth; 4) and the use of an appropriate cohort study design. Cardioversions that were successfully controlled during the fetal stage were considered successful, even if tachycardia recurred postnatally. The intrauterine death ratio was as used as a secondary endpoint in the treatment arms, while postpartum deaths were not included. The exclusion criteria included 1) AET and PJRT, due to inadequate sample size; 2) failure to report the primary outcomes of interest; 3) the initial diagnosis occurring after 37 gestational weeks; 4) the study did not distinguish between specific types of fetal tachycardia among the enrolled cases.

Non-treatment and placebo treatments are clinically unfavorable for patients and are ethically disallowed for consistent fetal tachycardia; therefore, every regimen was used as the reference baseline for all network meta-analysis comparisons for the results of each set of two-by-two comparisons can be clearly described. In this analysis, we only evaluated the therapeutic efficacy of first-line transplacental medication administration on fetal tachycardia and assessed the associated safety of each treatment option. We also collected the publication year of the study, the dosage of treatment for existing CHD comorbidities, and the occurrence of both maternal and neonatal adverse events.

### Study Quality Assessment

The risks of bias and article quality were assessed with the Newcastle–Ottawa Scale (NOS) (Wells et al., 2014) according to 3 aspects: Selection (4 stars), Comparability (2 stars), Outcome (3 stars). Publication bias was assessed using funnel plots in Stata, version 16.

### Data Assessment and Statistical Analysis

Five therapeutic regimens were evaluated, including monotherapies and combination therapies, namely D monotherapy, DF combination therapy, F monotherapy, DS combination therapy, and S monotherapy*,* data of which were then fed into the model (see Supplementary Materials). The sotalol and flecainide combination therapy, amiodarone monotherapy, and verapamil monotherapy were excluded from the meta-analysis due to inadequate data availability. Five subgroups were established according to the type of tachycardia, including total (means all the included patients), SVT, AF, hydrops, and non-hydrops groups. We used a Bayesian hierarchical model to establish a network analysis framework and used Markov Chain Monte Carlo (MCMC) simulation procedures to generate posterior distributions by R package (gemtc) (Harrer et al., 2021).

In Bayesian statistics, we calculated credible intervals (Crl) rather than confidence intervals around our estimates, and a 95% credible interval indicating that ‘there is a 95% probability that the true value of the parameter falls within our interval’ rather than ‘of every confidence interval calculated with each data set, 95% of the credible intervals (Crl) contain the true values of the parameters’ in frequentist statistics. The Bayesian hierarchical model uses uninformative priors on the effect in every comparison which doesn’t have a big impact on the posterior results, and the effects of a multi-arm study stem came from a multivariate (normal) distribution (Harrer et al., 2021). The model considered the replaceability of each treatment and used collected direct and indirect data based on uninformative priors, in order to obtain a relative accurate posterior result. The MCMC sampling allows to estimate the posterior distributions of our parameters, and thus generate the results of network meta-analysis (Harrer et al., 2021). Due to the inability to control for variables during observational studies, we did not assume that each study is an estimator of the same true effect size, but there are “study-specific” true effects estimated by each observed effect size and they are part of an overarching distribution of true effect sizes, and the variation between studies was estimated using a random-effects model.

A network plot was generated for the primary endpoint in each group using a random-effects model, using four Markov chains, 5,000 burn-in iterations, and 20,000 simulation iterations, where the parameters were considered enough for sensitive and well-converging network models, details on parameters could be accessed by command: *?mtc.run* of R package (gemtc) (Harrer et al., 2021). Convergence assessments were performed using Gelman–Rubin plots. This plot used the term Potential Scale Reduction Factor (PSRF) to compare the variation within each chain to the variation between chains, and how both develop over time. The PRSF would gradually shrink down with increasing numbers of iterations, and should at least be below 1.05 in the end for an enough converged model. We also evaluated the consistency of our network model using the node-split method. Using the network meta-analysis results, we calculated the probability of a treatment being the first, second, third, fourth, or fifth ranking treatment; presented the comparison in one cumulative probability plot; data of each set of two-by-two comparisons would be arranged in a table. We only represented forest plot by comparing with D monotherapy. The same approach was used to assess the secondary outcome of mortality across the whole population.

We integrated the safety and efficacy data in the total tachycardia population by introducing a metric we referred to as the term *safety index*, which was calculated as the number of deaths divided by the number of successful cardioversions, to simultaneously assess the risks and benefits for each therapeutic regimen. It was derived from a pharmacologic concept——therapeutic index, which evaluates the safety of a drug and calculated as the median lethal dose divided by median effective dose. We hypothesized that a higher safety index value indicates reduced benefits of a regimen, because there were more death events with the same recovery events. Different with the comparison barely on death rate and cardioversion rate, safety index may complement the analysis of the data. For example:

-In study A, 20 of D group for first-line treatment and 10 of F, 12 of D for cardioversion and 7 of F, and 1 of D for death and 1 of F.
-In study B, also 20 of D group for first-line treatment and 10 of F, but 11 of D for cardioversion and 8 of F, and 4 of D for death and 2 of F.
-For cardioversion, the rate shows 23/40 of D < 15/20 of F, but for death, 5/40 of D < 2/20 of F, we may tell F is more efficiency but more unsecure.
-But as for safety index, 5/23 of D > 3/15 of F, we find that D would have more death events with the same recovery events. Two conclusions are in conflict in this example.

From our point of view, the difference may have relation with existence of the unrecovered but also alive population after first-line therapy, causing some data hidden behind. So safety index does provide another aspect for explaining the results.

The R (gemtc) package (van Valkenhoef et al., 2012) and Stata were used to perform statistical calculations for this network meta-analysis (Salanti, 2012; Shim et al., 2017; Shim et al., 2019; Harrer et al., 2021). We have put the R code and collected data in Supplementary Materials for convenience of replication.

## Results

### Study Inclusion and Data Extraction

**FIGURE 1:**
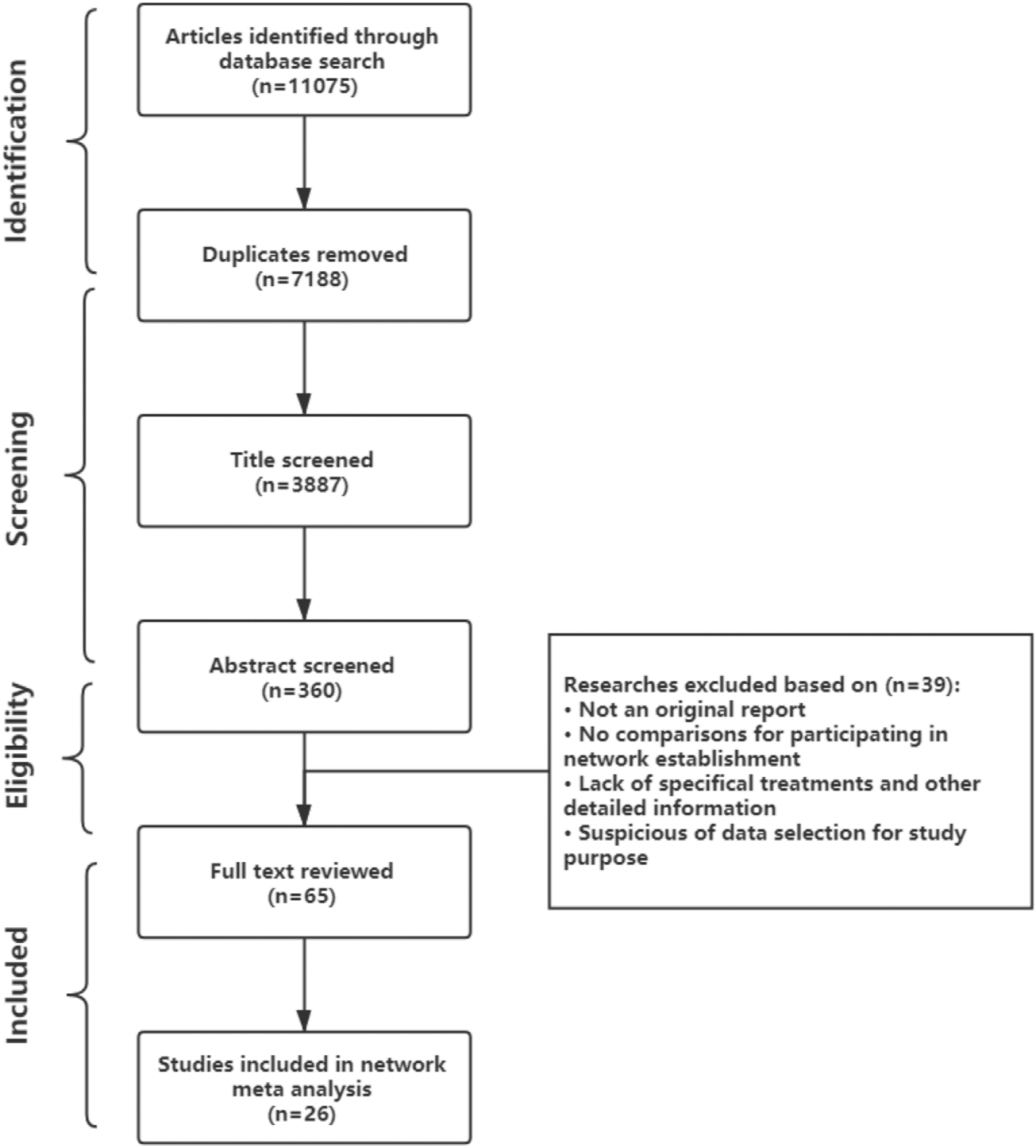
Preferred Reporting Items for Systematic Reviews and Meta-Analyses (PRISMA) flowchart describing study selection.

After the database search, 11,075 publications were identified, and 26 observational studies satisfied the established inclusion and exclusion criteria after screening and eligibility procedures were applied (shown in Figure 1). Any discrepancies were resolved between two reviewers by discussion until consensus was reached. Among the 26 included studies, one was a prospective study, and 25 were retrospective studies (van Engelen et al., 1994; Frohn-Mulder et al., 1995; Naumburg et al., 1997; Lisowski et al., 2000; Oudijk et al., 2000; Ebenroth et al., 2001; Jouannic et al., 2002; Krapp et al., 2002; Boldt et al., 2003; Jouannic et al., 2003; Oudijk et al., 2003; D'Alto et al., 2008; Pézard et al., 2008; Lulic Jurjevic et al., 2009; Hahurij et al., 2011; Shah et al., 2012; Uzun et al., 2012; van der Heijden et al., 2013; Ekman-Joelsson et al., 2015; Sridharan et al., 2016; Strizek et al., 2016; Ekiz et al., 2018; Karmegeraj et al., 2018; Miyoshi et al., 2019; O'Leary et al., 2020; Tunca Sahin et al., 2021). Only one randomized controlled trial (RCT; www.fasttherapytrial.com) (Edgar Jaeggi, MD, etc.) was retrieved during this search, but this RCT has not finished; therefore, it was treated as gray literature. Detailed reasons for the exclusion of identified articles are provided in Figure 1. The characteristics of the 26 included studies were summarized in Supplementary Table S1. 21 studies were conducted in Europe with 7 in Netherlands, and 3 studies were established in United States, 1 in Japan, 1 in India. Included gestational age at birth or when outcome recorded was ranged from 23 to 42 weeks but concentrated in 38 weeks. Quantitative data were organized into five groups according to treatment (D, DF, F, DS, and S). The maternal, neonatal and follow-up adverse events data were extracted regardless of the tachycardia type and specific regimens, because this information was not always reported in detail. The data set of extracted results that were used for statistical analyses are presented in the Supplementary Materials.

### Combination Therapy of Digoxin and Flecainide Revealed Superiority for Cardioversion

**FIGURE 2:**
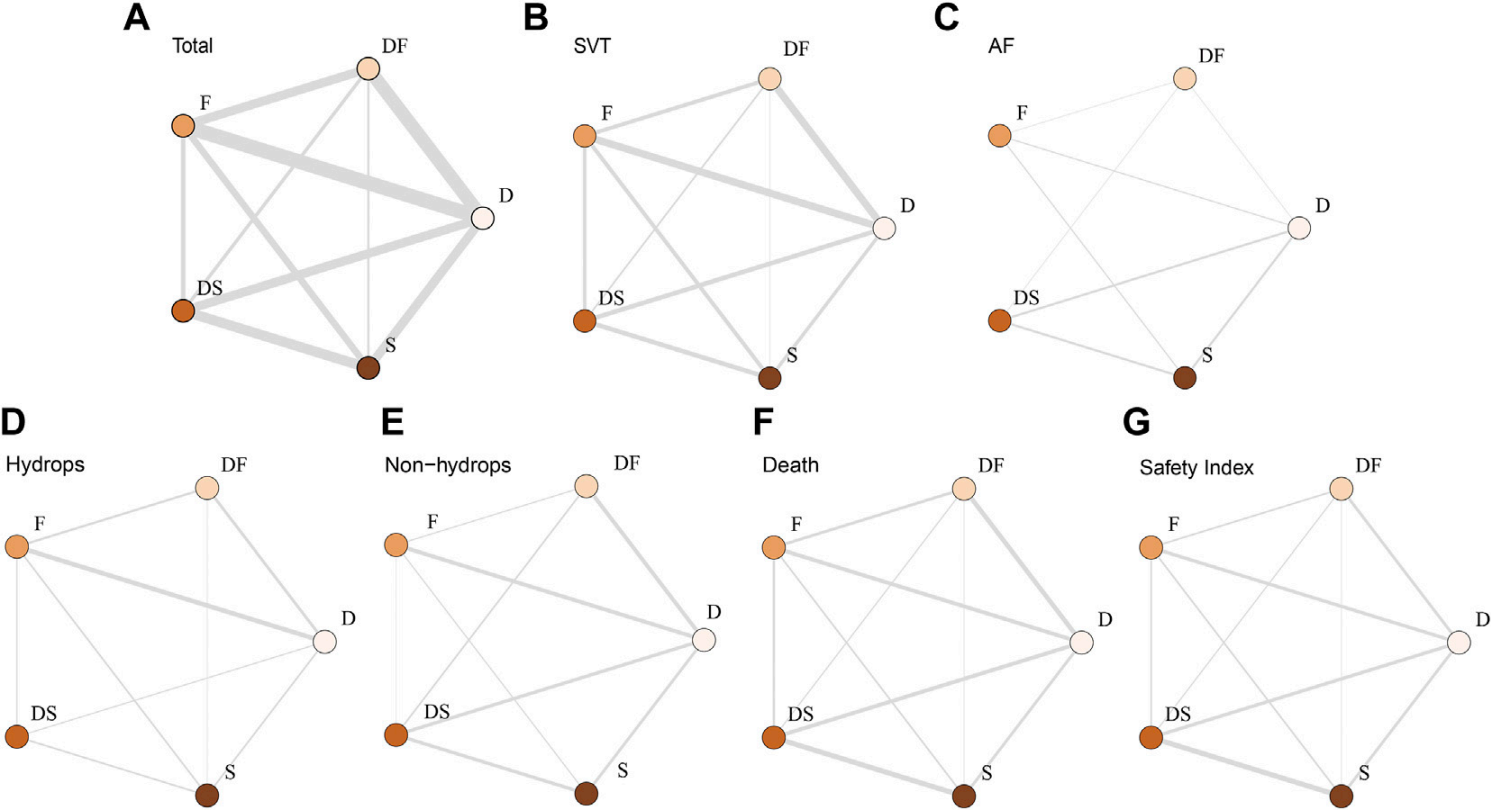
Network plot of the enrolled analyses. The network plots represent comparison sizes between two regimens, with edge thickness representing the number of related studies. **(A)** Total group. **(B)** SVT group. **(C)** AF group (lacking any direct comparisons between F and DS). **(D)** Hydrops group (lacking any direct comparisons between DF and DS). **(E)** Non-hydrops group (lacking any direct comparisons between DF and DS). **(F)** Death rate in the total group. **(G)** Safety index in the total group. D, digoxin monotherapy; DF, digoxin and flecainide combination therapy; DS, digoxin and sotalol combination therapy; F, flecainide monotherapy; S, sotalol monotherapy; SVT, supraventricular tachycardia; AF atrial flutter.

**FIGURE 3:**
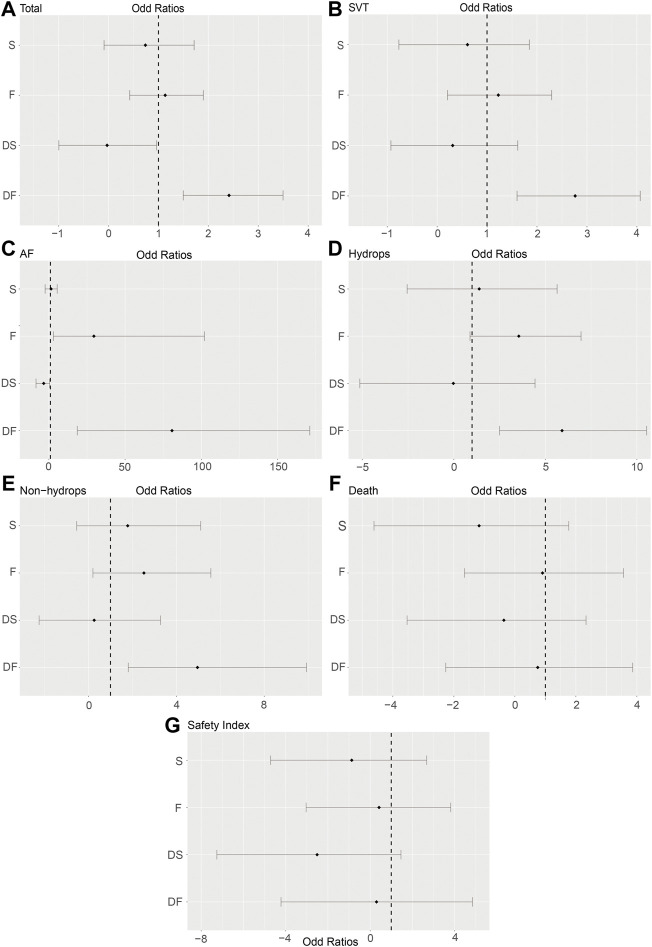
Forest plots of the enrolled analyses. The forest plots showed odds ratios (ORs) and 95% credible intervals (95% CrI) comparing the effectiveness and risks among five regimens, using D as the baseline reference (OR = 1). **(A)** Total group. **(B)** SVT group. **(C)** AF group. **(D)** Hydrops group. **(E)** Non-hydrops group. **(F)** Death rate in the total group. **(G)** Safety index in the total group. D, digoxin monotherapy; DF, digoxin and flecainide combination therapy; DS, digoxin and sotalol combination therapy; F, flecainide monotherapy; S, sotalol monotherapy; SVT, supraventricular tachycardia; AF atrial flutter.

**FIGURE 4:**
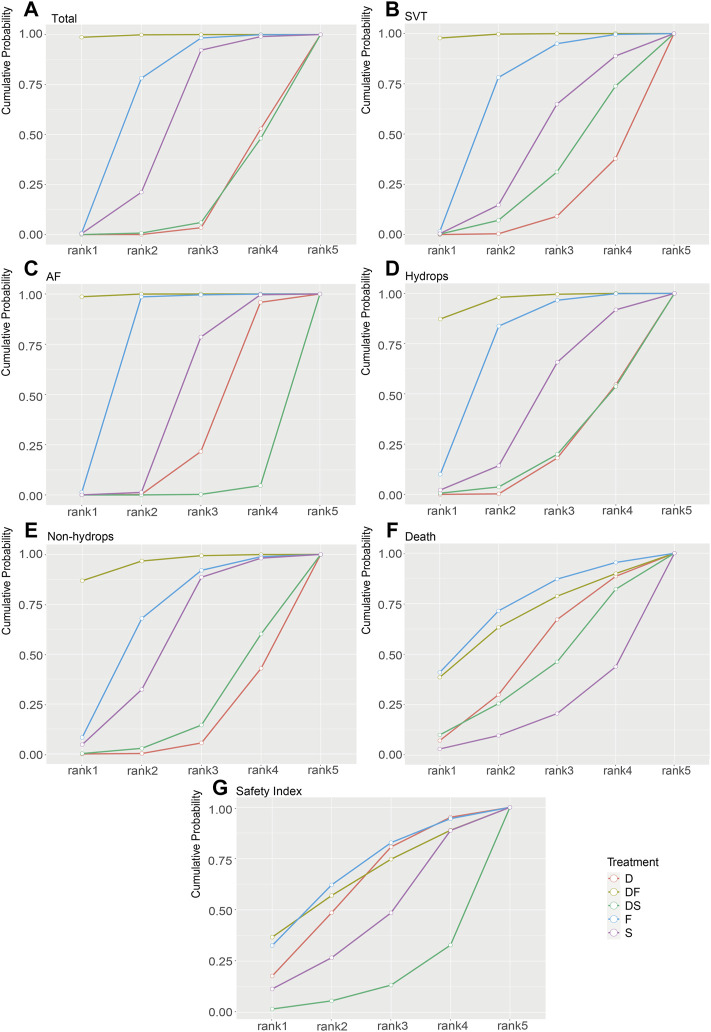
Rank-line plots of the enrolled analyses. The cumulative probability of superiority is shown as a line chart, and the area under the curve (AUC) represents the rankings, with a larger AUC indicating a higher rank. **(A)** Total group. **(B)** SVT group. **(C)** AF group. **(D)** Hydrops group. **(E)** Non-hydrops group. **(F)** Death rate in the total group. **(G)** Safety index in the total group. D, digoxin monotherapy; DF, digoxin and flecainide combination therapy; DS, digoxin and sotalol combination therapy; F, flecainide monotherapy; S, sotalol monotherapy; SVT, supraventricular tachycardia; AF atrial flutter.

For the primary outcomes, the data volumes and model stability were assessed for each of the five groups. The network plots represented the data size and the comparison size between two regimens, and the edge thickness corresponded to the numbers of studies included for comparisons. Total, SVT, and hydrop*s* groups were found to form a complete network structure, with relationships identified across all treatment regimens; by contrast, some direct and indirect comparisons were lacking for the AF and non-hydrops groups, including F vs. DS and DF vs. S, suggesting that more attention should be paid when interpreting the results for these two groups (Figures 2A–E). Gelman–Rubin plots indicated that the shrink factor was stably maintained below 1.05 for all five groups (Supplementary Figures S1–S5), indicating enough converged models being applied. To assess inconsistencies, the node-split forest plots (Supplementary Tables S2–S8) revealed the effects of various comparisons when using only direct, only indirect, or all available evidence. Inconsistency was identified in all five groups due to one or more comparisons with *p* < 0.05, which indicated that the studies included systematically different populations. As shown in Figures 3A–E, 4A–E, the top two best-ranked regimens were consistently identified as DF and F. The odds ratio (OR) and 95% credible interval (95% CrI) when compared with D as the reference therapy was significantly different across all groups for DF: Total, 2.44 (95% CrI: 1.59, 3.52); SVT, 2.77 (95% CrI: 1.59, 4.07); AF, 67.85 (95% CrI: 14.25, 168.68); hydrops, 6.03 (95% CrI: 2.54, 10.68); and non-hydrops, 5.06 (95% CrI: 1.87, 9.88). Other therapies showed significant effects for specific groups when compared with different regimens, details were shown in Table 1. The results indicated there wasn’t one regimen having all statistically significant differences when comparing to the other therapies. Relatively, DF and F administrations demonstrated better efficacy for controlling fetal heart rate, regardless of the tachycardia type or the presence of hydrops, indicating the superiority of the two treatment protocols. DF demonstrated superiority on D in all five subgroups. In total group and AF group, DF had better effectiveness than DS. In AF group, superiority of F regimen could also be observed. Majority of comparisons showed no significant differences.

**TABLE 1:** OR (95% Cl) data from the total, SVT, AF, hydrops, and non-hydrops groups.

OR (95% CrI)-column vs. row					
Total group					
	D	DF	F	DS	S
D		#	#	0.02 (−0.99–1.01)	#
DF	2.44 (1.51–3.52)		1.29 (0.23–2.48)	2.47 (1.19–3.86)	1.68 (0.41–2.97)
F	1.15 (0.42–1.93)	#		1.17 (0.04–2.32)	0.39 (−0.71–1.38)
DS	#	#	#		#
S	0.76 (−0.1–1.76)	#	#	0.79 (−0.19–1.84)	
**SVT group**					
D		#	#	#	#
DF	2.77 (1.59–4.07)		1.53 (0.13–3.04)	2.46 (0.8–4.15)	2.17 (0.56–4.01)
F	1.22 (0.21–2.31)	#		0.92 (−0.57–2.38)	0.63 (−0.73–2.14)
DS	0.31 (−0.96–1.64)	#	#		#
S	0.61 (−0.78–1.84)	#	#	0.29 (−1.24–1.63)	
**AF group**					
D		#	#	3.4 (−0.51–8.4)	#
DF	67.85 (14.25–168.68)		32.39 (0.95–130.4)	71.54 (17.57–172.23)	66.32 (12.66–167.25)
F	26.75 (2.05–96.94)	#		30.36 (5.19–100.28)	25.31 (0.5–95.34)
DS	#	#	#		#
S	1.44 (−2.51–5.49)	#	#	4.87 (1.06–9.78)	
**Hydrops group**					
D		#	#	#	#
DF	6.03 (2.54–10.68)		2.33 (−1.45–6.87)	6.03 (0.83–12.98)	4.56 (−0.25–10.31)
F	3.64 (0.9–7.11)	#		3.63 (−0.88–9.52)	2.19 (−2.18–7.11)
DS	0.01 (−5.17–4.52)	#	#		#
S	1.43 (−2.58–5.75)	#	#	1.43 (−2.43–6.12)	
**Non-hydrops group**					
D		#	#	#	#
DF	5.06 (1.87–9.88)		2.51 (−1.24–7.35)	4.8 (0.89–9.86)	3.24 (−1–8.21)
F	2.53 (0.24–5.5)	#		2.29 (−1.11–5.92)	0.73 (−2.84–4.08)
DS	0.26 (−2.23–3.22)	#	#		#
S	1.79 (−0.48–5.03)	#	#	1.55 (−0.86–4.38)	

*To clearly observe and interpretate the results, we use ‘#’ to replace the opposite and negative OR (95% CrI) in the cell that corresponds to another cell with a positive OR (95% CrI). For example, in Total group, (1, 2) or (D, DF) equal to 2.44 (1.51, 3.52) meant patients with DF (column) are 2.44 times more likely to obtain the reversion to a normal sinus rhythm than D (row), with a confidence interval at (1.51, 3.52). However, in network meta-analysis, (2, 1) or (DF, D) equal to -2.44 (−1.51, −3.52) exactly, so ‘#’ was left to replace and avoid confusion. A red background emphasized statistically significant result.

All network meta-analysis comparisons are represented in table but only Digoxin as baseline in forest plot (Figure 3). As shown in Table 1, DF demonstrated superiority on D in all five subgroups. In total group and AF group, DF had better effectiveness than DS. In AF group, superiority of F regimen could also be observed. Majority of comparisons showed no significant differences.

OR, odds ratio; CrI, credible interval; D, digoxin monotherapy; DF, digoxin and flecainide combination therapy; DS, digoxin and sotalol combination therapy; F, flecainide monotherapy; S, sotalol monotherapy; SVT, supraventricular tachycardia; AF, atrial flutter.

### Intrauterine Death

To assess the secondary outcome of death rates, a network plot (Figure 2F), Gelman–Rubin plot (Supplementary Figure S6), and inconsistency test (Supplementary Table S7) were used to examine intrauterine death. The results demonstrated no significant differences, indicating no differences in intrauterine death outcomes across the examined treatment regimens (Figure 3F and Table 2). As shown in Figure 4F, the two treatments found to be the most efficacious, DF and F, were also the top-ranked regimens for the death rate assessment. Therefore, we attempted to further assess the benefits and risks of each treatment regimen by analyzing the safety index.

**TABLE 2:** OR (95% CI) data from the total group for the death rate and safety index according to treatment group.

OR (95% CrI)-column vs. row					
Death rate group					
	D	DF	F	DS	S
D		#	#	0.35 (−2.34, 3.45)	1.15 (−1.84, 4.56)
DF	0.77 (−2.25, 3.89)		2.35 (−1.31, 7.44)	1.15 (−2.57, 5.27)	1.96 (−2, 6.27)
F	0.9 (−1.63, 3.61)	0.11 (−3.22, 3.62)		1.27 (−1.96, 4.96)	2.07 (−1.38, 6.01)
DS	#	#	#		0.79 (−1.88, 3.49)
S	#	#	#	#	
**Safety index group**					
	**D**	**DF**	**F**	**DS**	**S**
D		#	#	2.53 (−1.35, 7.39)	0.89 (−2.6, 4.79)
DF	0.37 (−4.17, 4.84)		#	2.92 (−2.5, 9.09)	1.26 (−3.97, 6.76)
F	0.47 (−2.97, 3.84)	0.02 (−4.44, 4.99)		3 (−1.55, 8.46)	1.35 (−2.9, 5.95)
DS	#	#	#		#
S	#	#	#	1.66 (−1.71, 5.44)	

All network meta-analysis comparisons are represented in table but only Digoxin as baseline in forest plot (Figure 3). As shown in Table 2, no significant difference existed in death rate group, but S appeared to be more safe though less efficacious. After considering both effectiveness and safety in the safety index group, no significant differences were observed in all five regimens.

OR, odds ratio; CrI, credible interval; D, digoxin monotherapy; DF, digoxin and flecainide combination therapy; DS, digoxin and sotalol combination therapy; F, flecainide monotherapy; S, sotalol monotherapy; SVT, supraventricular tachycardia; AF, atrial flutter.

### Safety Index

It was necessary to mention that only the data of cardioversion must be no zero to assess a safety index, studies with substandard data were removed, resulting in a loss of data volume. For example, data of Jouannic, J, M. et al. in Supplementary Materials as we provided online, the cardioversion for total were 0 in D and DS groups, so we had to remove both data for code running. Figure 2G and Supplementary Figure S7 show acceptable results, but the inconsistency assessment (Supplementary Table S8) indicated an increase in the number of inconsistent comparisons, reducing the credibility of the cumulative probability plot. Table 2 showed the absence of significant results, preventing the determination of absolute ranking among these five regimens (Figures 3G, 4G). To summarize, the DF and F regimens demonstrated relatively superior efficacy for terminating fetal tachycardias, regardless of the tachycardia type or the presence of hydrops, with no significant difference in the associated risks such as death rate.

### Quality Evaluation and Publication Bias

Supplementary Table S9 indicated that a low risk of bias was determined for all 26 studies included in this network meta-analysis (all studies obtained stars ≥7 (9 stars in total)) The publication bias of primary endpoints in the five groups is shown in Supplementary Figure S8, and no evident bias was observed for the total, SVT, hydrops, or safety index subgroups.

## Discussion

Oudijk et al. (2002) raised it very early that digoxin was the most common drug used but with effectiveness needing discussion, and flecainide was very effective in the control of fetal SVT based on their previous work and contribution. Jaeggi et al. (2011) established the response rate curve for digoxin monotherapy, sotalol monotherapy, and flecainide monotherapy over time, proposing associations between the fetal response to placental treatment and the fetal status, tachycardia type, and the type of anti-arrhythmia drug. Two meta-analyses reported by Hill et al. (2017) and Alsaied et al. (2017) both reached the same conclusion that flecainide monotherapy appears to be more effective as first-line therapy for fetal tachycardias than digoxin. In our results, after dividing the sample into four subgroups, the characteristics of the overall population were evaluated using network meta-analysis methods, which revealed that DF and F performed better than D, DS, and S in all five groups. In the AF group, significant differences were observed for multiple therapies. As secondary outcomes represented, no significance were observed among five regimens. Our results support the superiority of flecainide, particularly when combined with digoxin. As data showed, we suggested flecainide as the first-line regimen for balance between curative effect and side effects from monotherapy, digoxin should be promptly supplemented when flecainide monotherapy is ineffective to control fetal tachycardia as early as possible. Sotalol is considered for treatment alone or combined with digoxin when patients do not respond to above regimens. For AF group, DF and F come first, then S, finally DS and D.

We developed the safety index used in this study based on a pharmacology concept known as the therapeutic index, which is equal to the median lethal dose divided by the median effective dose. We borrowed the implication that a larger safety index value indicates reduced safety. Consequently, no differences were observed in results, which means there are similar amount of death events with the same amount of cardioversion events. However, due to the lack of detailed safety results provided by many of the studies included in this meta-analysis, inconsistencies were observed during the safety index analysis, suggesting that these outcomes should be considered with caution.

In addition, according to the Supplementary Materials on structural heart malformations we collected, in 26 inclusions, 3 studies didn’t mention whether they had included fetuses with malformations, 4 studies mentioned but lacked of data, 10 studies clarified exclusion of structurally impaired patients, and 9 studies provided data for malformations. Generally, there could been identified that 34 patients with malformations included in our network meta-analysis, accounting for 4.25% of total amount of data (799 patients). Because most of the literatures did not provide a detailed description of the treatment groups to which each structurally impaired patients belonged, we could not exclude in the raw data of the meta-analysis and therefore inform here. Existence of cases of cardiac malformations may affect the results and adverse events associated with these regimens in newborns.

Other extracted data included maternal, neonatal and follow-up adverse events. Reporting of the maternal adverse events were less than that of neonatal and follow-up (Supplementary Materials). We found that, all mothers’ safety were guaranteed with priority and most mothers could tolerate treatment. Apart from common side effects such as nausea, headache, or transient blurred visions, serious events like cardiac electrophysiological abnormalities could be resolved after dose reduction or change of treatment, however, which could have an influence on the continuity of treatment of the fetus. Also of interest, it seemed maternal side effects were less in flecainide than digoxin. As dosage data we collected in Supplementary Materials, most researchers were strict of the dosage, and were flexible after receiving feedback on the effect and adverse reactions of medication. Although the dosage is importantly affecting the effect of fetal treatment, due to consideration on the tolerance of the mothers, we could not obtain clear relationship between the dosage and the efficacy. The neonatal and follow-up adverse events have been also collected: relapses of tachycardia were the most common events in alive infants with or without successful cardioversion, such as Wolff-Parkinson-White syndrome, permanent junctional reciprocating tachycardias, ectopic atrial tachycardia, etc. They were receiving antiarrhythmic drugs either for recurrence and prophylaxis, and most recovered or had been stably controlled. But for patients companied with severe hydrops or other complex complications, though receiving therapies such as drug regimen (furosemide, isoprenaline, dopamine, digoxin, propafenone, propranolol, verapamil or adenosine, etc.), electric cardioversion, radiofrequency catheter ablation or even surgical cardiac repair according to the individual condition, part of infants could not survive of severe heart failure or may suffer from central nervous system complications for a life-long time, but our meta-analysis only took intrauterine death into account to avoid mixing fetal therapy and neonatal regimens. Based on the textual descriptions of the neonatal situation and follow-up records collected in various articles, we found that over time, the number of treatments available to children in the postnatal period increased, adverse events other than the nervous system were mostly controlled, and the age for all-cause death was increasingly concentrated in the neonatal period or related with premature birth which may due to incomplete development of the fetus, while neonatal death patients often had obvious comorbidities. Children who passed the neonatal period seemed to have better outcomes. This suggested that we are in need of more experiences, on the one hand, to ensure that pregnant fetuses can safely survive the developmental period *in utero*, and the other, to take precautions against and control neonatal comorbidities.

The etiological implications of SVT and AF are commonly thought to be associated with abnormalities of the sinus node, the atrioventricular junction, or the ventricles. Atrioventricular reentrant tachycardia (AVRT) accounts for 90% of feta tachycardias (Jaeggi et al., 2011), originating from an accessory pathway between the atria and ventricles that creates an extra electrical circuit. Typically, AVRT has a characteristic ventricular-atrial (VA) interval, with an atrial-ventricular ratio <1, indicating a short VA tachycardia. Most hydrops is associated with AVRT, which could affect treatment. Digoxin cannot pass completely through the placenta; therefore, even when the maternal dose approaches toxic levels, the fetal drug level may not be sufficient; therefore, flecainide and sotalol are recommended for the treatment of hydrops. AF, however, frequently develops during late pregnancy (Jaeggi et al., 2011) due to premature atrial contraction (Wacker-Gussmann et al., 2016). In AF patients, atrial rates are commonly much faster (300–500 bpm) and twice the ventricular rates because of a physiological block at the atrioventricular node, but the degree of blockage varies. Moreover, AF can also coexist with AVRT in the same fetus (Jaeggi et al., 2011). Although hydrops is a significant lethality factor, the effects of hydrops on drug transformation and distribution and the changes in the electrophysiological characteristics of drug responses require more in-depth study. The primary effect of flecainide is the significant inhibition of sodium ion influx. When used in large doses, flecainide acts as a β-receptor blocker that can block calcium ion channels. Digoxin can inhibit the sodium–potassium pump and increase the frequency of sodium–calcium exchange. By reducing the autonomy of the sinus node, digoxin slows the heart rate. When digoxin and flecainide are combined, they simultaneously reduce the heart rate through mutually beneficial antagonistic effects. Sotalol blocks β1 receptors and reduces sympathetic excitability, with little direct influence on cardiomyocytes, which may explain its relative safety and lack of strong curative effects. However, this article was unable to infer any clear relationships between the observed results and the pharmacological effects of these drugs from a deeper perspective.

In clinical practice, depending on the emergency level after assessing the tachycardia type, hemodynamic consequences, fetal development, and maternal choice, doctors can choose no intervention, drug therapy, or delivery. However, pharmacological therapy and delivery are not mutually exclusive options. Treatment is recommended until the delivery of a full-term baby, if permitted, with a strong correlation observed between postnatal SVT and later gestational age at fetal SVT diagnosis (Hinkle et al., 2017). In our data of neonatal and follow-up adverse events, premature infants often related with worse outcomes. However, the presence of significant polyhydramnios may have to give birth for safety or mother (Carvalho, 2019).

Currently, network meta-analyses are developing methods to result in higher accuracy. Some researchers indicated that the precise classification of scientific questions in network meta-analyses would help identify specific statistical methods and more customized model options for addressing medical questions. For example, in a network meta-analysis of drug efficacy, if the dose data is collected, a dose–response network meta-analysis can be performed to obtain a more reliable interpretation. However, we did not discuss this relationship in detail because dosage details are often difficult to collect.

Future RCTs could continue to apply Bayesian hierarchical models and verify the outcomes of this article. The existing model can also be optimized to facilitate the combine the two research designs. Future RCT studies that provide individual patient data can be used for meta-regression analyses that evaluate the intervention effects according to more individualized data rather than average values.

### Strengths and Limitations

The use of Bayesian model analysis can prevent the second-class errors that ordinary meta-analyses are prone to producing when assessing multiple comparisons. In addition, our model incorporates a larger samples size (799 patients in total group, subgroups data could be accessed in Supplementary Materials) than previous meta-analyses.

Our included samples were limited to cases of SVT or AF, and other rarer manifestations were not included. In addition, Inconsistency identified in all seven data set indicated that the studies included systematically different populations, which were likely due to various causes, including differences in the severity of tachycardia, the gestation period, drug doses, and administration routes other than placental administration, such as muscular injection, umbilical vein injection, or oral administration, which were difficult to unify or control across these 26 observational studies. It should be noted that most of the samples in this paper have normal heart structure, so the conclusions in this paper are more suitable for children with normal heart structure. Whether the included retrospective studies applied similar practical treatment conditions was difficult to determine, and retrospective observation data is unable to meet randomization requirements. Prospective studies and ongoing RCT studies would provide more methodological control. However, Efthimiou et al. (2017) proposed that the inclusion of evidence from non-random studies (observational studies) can improve the accuracy of the results obtained from RCT-based network meta-analyses; therefore, this article can provide supplemental data for any follow-up studies. Due to the complexity of the Bayesian model, which requires similar distributions by default, the results may be influenced by non-similar distributions.

## Conclusion

We conducted a network meta-analysis of first-line treatments used to manage fetal tachycardia using a Bayesian model. The superior effectiveness was remarkable for digoxin and flecainide combination therapy and flecainide monotherapy across all subgroup analyses (total, SVT, AF, hydrops, and non-hydrops). No significant differences in safety risks were identified among these therapies. Thus, the flecainide monotherapy and combination of digoxin and flecainide should be considered the most superior therapeutic strategies for fetal tachycardia. For details, it is suggested that flecainide acts as the first-line regimen for balance between curative effect and side effects from monotherapy, digoxin should be promptly supplemented when flecainide monotherapy is ineffective to control fetal tachycardia as early as possible. Sotalol is considered for treatment alone or combined with digoxin when patients do not respond to above regimens.

## Data Availability

The original contributions presented in the study are included in the article/Supplementary Material, further inquiries can be directed to the corresponding author.

## Supplementary Material

Supplementary Figure S1 Gelman Runbin plot of Total group. Gelman Runbin plots represented the model fitting results between each comparison, it indicated active results if the line’s shrink factor is stably below 1.05 as iterations increase. All Gelman Runbin plot (Supplementary Figures S1–S7) showed active results.

Supplementary Figure S2 Gelman Runbin plot of SVT group.

Supplementary Figure S3 Gelman Runbin plot of AF group.

Supplementary Figure S4 Gelman Runbin plot of Hydrops group.

Supplementary Figure S5 Gelman Runbin plot of Non-hydrops group.

Supplementary Figure S6 Gelman Runbin plot of Death group.

Supplementary Figure S7 Gelman Runbin plot of Safety Index group.

Supplementary Figure S8 Funnel plots of enrolled analyses. The funnel plots represented the publication bias of results in 7 groups, no significant bias were found in Total, SVT, AF, Death and Safety Index groups. Hydrops and Non-hydrops groups may have risks of publication bias. **(A)** Total group; **(B)** SVT group; **(C)** AF group; **(D)** Hydrops group; **(E)** Non-hydrops group; **(F)** Death rate in Total group; **(G)** Safety Index in Total group. D, Digoxin; DF, Digoxin and Flecainide; DS, Digoxin and Sotalol; F, Flecainide; S, Sotalol.
