# Supplementary material for: Efficacy and Safety of Various First-Line Therapeutic Strategies for Fetal Tachycardias: A Network Meta-Analysis and Systematic Review
Source: Front Pharmacol. 2022 Jun 13;13:935455. doi: 10.3389/fphar.2022.935455 (PMC9235149; doi:10.3389/fphar.2022.935455)
Supplement: Supplementary file 1 [file DataSheet2.PDF]

Table S1. Basic characteristics of included studies.

Author	Country	Year	Study design	Number of centers	Number Male/Female Fetus	Median or Mean GA at Diagnosis (wk)	Median or Mean GA at Birth	Administration Route	Morphological Abnormalities Number	Pre-term Birth Number in all	CS Delivery Number in all	Main Conclusion
van Engelen, A. D.	Netherlands	1994	Retrospective	3	NA	NA	NA	intravenous loading and oral maintenance	NA	NA	NA	Fetal tachycardia can be treated adequately in the majority of patients, even in the presence of hydrops, and therefore emergency delivery might not be indicated. **Digoxin and flecainide** were drugs of first choice and produced no serious adverse effects in this series of patients. The majority of patients do not require prolonged therapy.
Frohn-Mulder, I. M.	Netherlands	1995	Retrospective	1	NA	30 ± 5.8	NA	oral	NA	NA	NA	In our series, **flecainide** was shown to be more successful than digoxin in restoring sinus rhythm during fetal SVT. In the absence of fetal hydrops digoxin is a potent drug for the conversion of tachycardia into sinus rhythm. Where digoxin fails to restore sinus rhythm, flecainide may be successful. In hydropic fetuses there is a high risk of perinatal mortality, since transplacental drug transfer is hampered and digoxin, therefore, appears to be of limited value. Verapamil has serious side

												effects and should be avoided as a mode of treatment. Furthermore, in our experience, administration of flecainide is associated with a significant reduction in perinatal mortality, with no adverse side effects being observed.
Naumbu rg, E.	Sweden	1997	Retrospective	1	NA	34(23~37)	37.5(33~40)	oral or intravenous	NA	11	10	Although fetal tachycardia is a serious condition, antenatal treatment in combination with careful monitoring and induction of delivery in cases with deteriorating fetal condition result in a satisfactory outcome for the majority of infants. However, there is a risk of late recurrence.
Oudijk, M. A.	Netherlands	2000	Retrospective	2	NA	21~37	39 in non-hydrop group, 35 in hydrops group	oral	NA	NA	NA	**Sotalol** and the combination of **sotalol and digoxin** were very successful in the AF group, with a conversion rate as high as 80% and the advantage of a low recurrence rate. But the evidently present risks should be weighed against the limited benefits of sotalol therapy for fetal SVT. It is our belief that the use of sotalol should be restricted to cases with fetal AF and those cases of fetal SVT in which other treatment options have failed.
Lisowsk	Netherlands	2000	Retrospective	Multiple	NA	31.5(19~4	NA	oral or	1	NA	10	Digoxin failed in prevention of

i, L. A.						0)		intravenous				recurrence at time of delivery in a quarter of our patients, whereas with sotalol no recurrence of AF has been reported, suggesting that **class III agents** may be the future therapy. Once fetuses with AF survive without neurological pathology, their future is good and prophylaxis beyond the neonatal period is unnecessary.
Ebenroth, E. S.	USA	2001	Retrospective	1	NA	NA	NA	oral	NA	NA	NA	**Digoxin** remains an effective first-line therapy in the treatment of fetal SVT. Flecainide is an effective second-line therapy, especially in the face of fetal hydrops.
Krapp, M.	Germany	2002	Retrospective	1	NA	27(21~35)	38(32~41)	intravenous loading and oral maintenance	NA	2	8	**Flecainide** is safe and highly effective in the intrauterine treatment of hydropic fetuses with supraventricular tachycardia. Conversion into sinus rhythm can be expected 72 h after initiation of therapy but may take up to 14 days. Therefore, therapy should be continued beyond 72 h, especially when an initial decrease of fetal heart rate is observed which may represent an early therapeutic response.
Jouannic, J. M.	France	2002	Retrospective	1	NA	29(24~38)	29±4.9	intravenous loading and oral maintenance	1	NA	NA	The response to prenatal therapy may be poorer in the presence of tricuspid regurgitation and that

												screening for minor signs of ventricular dysfunction could become part of the initial evaluation of SVT without hydrops.
Jouannic, J. M.	France	2003	Retrospective	1	NA	29(19~37)	NA	oral	NA	NA	1	When first-line therapy fails to restore sinus rhythm in hydropic fetuses with supraventricular tachycardia, **amiodarone** therapy should be considered as it allows a substantial number of fetuses to be converted prenatally.
Boldt, T.	Finland	2003	Retrospective	1	164/128	31.3(15~41)	38.4(23~42)	oral mainly, also including fetal intravenous, direct fetal peritoneal if amiodarone was used	17	NA	NA	All fetal arrhythmias except atrial extrasystoles were associated with a moderately high risk for fetal distress. In cases of compromise, fetal and neonatal prognosis was poor and was an indication for perinatal medication. After the newborn period, the prognosis has been good. However, the risk for neurologic morbidity must be taken into consideration.
Oudijk, Martijn A.	Netherlands and Switzerland	2003	Retrospective	1	NA	NA	NA	oral	NA	NA	NA	**Sotalol** is a potent antiarrhythmic agent in the treatment of fetal tachycardia. The placental transfer is excellent. Sotalol accumulates in amniotic fluid but not in the fetus itself. Therefore, it seems that renal excretion in the fetus is efficient and greater than the oral absorption by fetal

												swallowing. The maternal blood level is not a reliable predictor of the chances of success of therapy. Sotalol is not associated with fetal growth restriction.
D'Alto, M.	Italy	2006	Retrospective	1	NA	24.3 ± 4.5	NA	intravenous loading and oral maintenance	8	NA	NA	Fetal echocardiography could clarify the electrophysiological mechanism of fetal cardiac dysrhythmias and guide the therapy.
Pezard, P. G.	France	2008	Retrospective	1	NA	30.1±4.6	NA	transplacental	0	NA	7	Maternal oral amiodarone seems to be effective and relatively safe, even in hydropic fetuses. We suggest that this treatment could be used earlier than is currently advised.
Lulic Jurjevic, R.	Slovenia	2009	Retrospective	1	12/14	34 (23~39)	38 (33~40)	transplacental	4	NA	4	A significant proportion of fetal tachycardias recognized before 36 weeks of gestation can be treated successfully by transplacental administration of antiarrhythmic drugs. Fetuses presenting after 36 weeks of gestation can be effectively managed postnatally. The long-term prognosis for fetuses diagnosed with tachycardia is excellent, with the abnormal rhythm resolving spontaneously during the first year of life in most of them.
Hahurij, N. D.	Netherlands	2011	Retrospective	1	NA	NA	NA	oral mainly, also including fetal	NA	NA	NA	Mortality rate is low in patients with fetal SVT and AF but high

								intravenous, direct fetal peritoneal if amiodarone was used				in patients with AVB. Related morbidity includes WPW-syndrome and congenital cardiac anomalies. Electrocardiographic screening is recommended in all fetal SVT cases before adolescence since WPW-syndrome may occur later in life.
Shah, A.	USA	2012	Retrospective	1	NA	25(17~35)	NA	oral	NA	NA	NA	**Sotalol** is a much more desirable medication to treat fetal tachyarrhythmias than either flecainide or amiodarone.
Uzun, O.	UK	2012	Retrospective	1	NA	29(18~38)	38(29~41)	oral	1	NA	9	**Digoxin and flecainide combination** offers a safe and effective treatment in fetal supraventricular tachycardia and atrial flutter. The beneficial effect of this combination has also been shown in hydropic fetuses. Both medications are well tolerated by fetuses as well as pregnant women, with no fetal demise or maternal pro-arrhythmia or serious side effects.
van der Heijden, L. B.	Netherlands	2013	Retrospective	1	NA	30.4±5.0	39.8±1.2	transplacental	1	NA	NA	Sotalol can be recommended as the drug of first choice for treatment of fetal AF and has been shown to be an effective and safe first-line treatment option for SVT, at least in the absence of hydrops. Postnatal maintenance therapy after successful prenatal therapy is not

												necessarily indicated, as the risk of recurrence is low beyond 72 hours of age.
Ekman-Joelsson, B. M.	Sweden	2015	Retrospective	2	Two-thirds of the fetus were boys	29.8 ± 3.4 for hydrops ,31.4 ± 4.8 for non-hydrops	29.9 ± 3.8 for cardioversion 33.3 ± 2.9 for non-cardioversion	oral or intravenous	NA	40	12	Transplacental treatment was frequently insufficient to obtain cardioversion in non-hydropic and hydropic fetus, but all non-hydropic cases had favorable outcomes. Larger prospective studies are needed to optimize the treatment of cases with hydrops.
Sridharan, S.	UK and Czech	2016	Retrospective	2	NA	30 (20~37) for F, 31 (19–41) for D	NA	oral and intravenous	NA	NA	NA	**Flecainide** was more effective than digoxin, especially when hydrops was present. No adverse fetal outcomes were attributed to flecainide.
Strizek, B.	Germany	2016	Retrospective	2	NA	29(22~37.4)	NA	oral and intravenous	4	NA	NA	**Flecainide** is highly effective in achieving SR in hydropic and non-hydropic fetuses with supraventricular tachycardia in a median time of 3 days. In our opinion, flecainide should be considered as first-line therapy in fetal supraventricular tachycardia with and without hydrops.
Ekiz, A.	Turkey	2018	Retrospective	1	NA	30.4±4.9	37(31~40)	intravenous loading and oral maintenance	2	NA	NA	In conclusion, the present study has demonstrated that **flecainide** is an effective first-line treatment for fetal SVT with high success rate (88.2%), low side effect profile and relatively easy utilization.

Karmegeraj, B.	India	2018	Retrospective	1	11/8	32.5±3.2 for SVT, 29.6±3.3	36.2±1.2 for SVT, 36.3±1.3 for AF	intravenous loading and oral maintenance, including intramuscular injection	0	NA	10 for SVT, 7 for AF	Aggressive transplacental therapy using combination of drugs achieves excellent pregnancy and postnatal outcomes in fetuses with tachyarrhythmia. Early diagnosis and prompt referral before hemodynamic decompensation is critical for ensuring optimal outcomes.
Miyoshi, T.	Japan	2019	Prospective	15	38/11	31(24~36)	38(29~40)	intravenous loading and oral maintenance	3	7	22 (only 3 cesarean sections were performed for fetal tachyarrhythmia.)	Protocol-defined transplacental treatment for fetal SVT and AFL was effective and tolerable in 90% of cases. Maternal AEs were observed in 78% of patients; however, most AEs were minor, and dose reduction allowed for continued protocol treatment.
Tunca Sahin, G.	UK	2021	Retrospective	1	NA	32.5(20~38)	38(28~41)	intravenous loading and oral maintenance	2	3	NA	Fetal AFL has a strong association with postnatal emergence of atrioventricular reciprocating tachycardia associated with ventricular pre-excitation. Therefore, a 12-lead ECG should be obtained and carefully reviewed in neonates who were diagnosed with AFL and required treatment as a fetus.
O'Leary, E. T.	USA	2021	Retrospective	1	31/26	30(14~37)	38(30~40)	transplacental	0	18	NA	Fetuses with structurally normal hearts and sustained SVT can be

effectively managed with transplacental drug therapy with minimal risk of intrauterine fetal demise. Treatment requires **multiple antiarrhythmic agents** in over half of cases. Later gestational age at fetal diagnosis and the presence of depressed fetal ventricular function, but not hydrops, predict postnatal arrhythmia burden.

All the studies were observational study designs; NA meant there were no clear description or precise statistical processing of data on the whole population; GA = Gestational age in completed weeks; CS = cesarean section;

Table S2. Nodesplit analysis of inconsistency of Total group

Comparison	OR(95% Cl)	Effect	P value
D vs DF	2.37 (1.45, 3.41)	direct	0.066975
	23.19 (2.09, 105.27)	indirect	
	2.44 (1.5, 3.52)	network	
D vs DS	-0.14 (-1.41, 1.11)	direct	0.878775
	-0.32 (-2.42, 1.86)	indirect	
	-0.03 (-0.96, 0.96)	network	
D vs F	1.25 (0.41, 2.24)	direct	0.50895
	0 (-3.79, 3.86)	indirect	
	1.14 (0.44, 1.91)	network	
D vs S	0.83 (-0.48, 2.37)	direct	0.859025
	0.58 (-2.33, 3.25)	indirect	
	0.75 (-0.08, 1.72)	network	
DF vs DS	-1.6 (-4.42, 0.99)	direct	0.39655
	-2.87 (-4.52, -1.4)	indirect	
	-2.46 (-3.81, -1.19)	network	
DF vs F	-2.15 (-3.99, -0.71)	direct	0.0466
	0.01 (-1.56, 1.82)	indirect	
	-1.29 (-2.47, -0.23)	network	
DF vs S	0.35 (-2.62, 3.35)	direct	0.131
	-2.16 (-3.64, -0.72)	indirect	
	-1.68 (-2.96, -0.45)	network	
DS vs S	0.84 (-0.53, 2.21)	direct	0.862775
	1.09 (-1.57, 4.08)	indirect	
	0.78 (-0.18, 1.82)	network	
F vs DS	-1.3 (-4.03, 1.05)	direct	0.974725
	-1.36 (-2.76, 0.13)	indirect	
	-1.17 (-2.27, -0.06)	network	
F vs S	-0.82 (-2.31, 0.59)	direct	0.29415
	0.39 (-1.45, 2.48)	indirect	
	-0.4 (-1.35, 0.67)	network	

D, Digoxin; DF, Digoxin and Flecainide; DS, Digoxin and Sotalol; F, Flecainide; S, Sotalol.

Table S3. Nodesplit analysis of inconsistency of SVT group

Comparison	OR(95% Cl)	Effect	P value
D vs DF	2.7 (1.46, 4.09)	direct	0.134025
	28.45 (-0.16, 70.32)	indirect	
	2.77 (1.58, 4.1)	network	
D vs DS	0.15 (-1.5, 1.82)	direct	0.839
	0.55 (-3.29, 4.57)	indirect	
	0.33 (-0.92, 1.63)	network	
D vs F	1.37 (0.12, 2.78)	direct	0.56685

	2.41 (-1.26, 6.09)	indirect	
	1.23 (0.21, 2.29)	network	
D vs S	1.3 (-1.02, 3.87)	direct	0.193475
	-1.14 (-5.36, 1.98)	indirect	
	0.61 (-0.78, 1.87)	network	
DF vs DS	-1.55 (-4.75, 1.49)	direct	0.439575
	-2.93 (-5.13, -1.01)	indirect	
	-2.45 (-4.13, -0.8)	network	
DF vs F	-2.42 (-4.68, -0.79)	direct	0.0477
	-0.03 (-1.69, 1.63)	indirect	
	-1.54 (-3.07, -0.12)	network	
DF vs S	1.45 (-3.96, 7.09)	direct	0.15005
	-2.58 (-4.7, -0.86)	indirect	
	-2.17 (-4.01, -0.55)	network	
DS vs S	0.34 (-2.04, 2.5)	direct	0.7909
	-0.22 (-4.47, 3.56)	indirect	
	0.29 (-1.25, 1.62)	network	
F vs DS	-2.12 (-5.74, 0.57)	direct	0.374575
	-0.54 (-2.77, 2.05)	indirect	
	-0.9 (-2.36, 0.57)	network	
F vs S	-2.71 (-6.41, -0.43)	direct	0.04955
	1.28 (-1.99, 5.41)	indirect	
	-0.62 (-2.14, 0.72)	network	

D, Digoxin; DF, Digoxin and Flecainide; DS, Digoxin and Sotalol; F, Flecainide; S, Sotalol.

Table S4. Nodesplit analysis of inconsistency of AF group

Comparison	OR(95% Cl)	Effect	P value
D vs DF	18.15 (-73.29, 173.42	direct	0.311275
	108.7 (24.74, 231.26	indirect	
	62.89 (14.75, 159.89	network	
D vs DS	-1.31 (-6.12, 2.57	direct	NA
	-88.5 (-179.03, -29.94	indirect	
	-3.35 (-8.35, 0.51	network	
D vs F	44.88 (2.96, 168.67	direct	0.366725
	-19.33 (-148.13, 120.75	indirect	
	25.01 (2.1, 93.35	network	
D vs S	0.56 (-3.05, 3.94	direct	0.281125
	45.29 (-50.56, 128.99	indirect	
	1.44 (-2.44, 5.47	network	
DF vs DS	13.75 (-70.68, 110.3	direct	0.057675
	-112.75 (-233.74, -27.79	indirect	
	-66.22 (-163.08, -17.77	network	
DF vs F	-43.4 (-137.35, -2.31	direct	0.407575

	14.75 (-112.01, 150.63	indirect	
	-30.07 (-123.39, -0.68	network	
DS vs S	5.28 (0.89, 10.54	direct	0.2415
	25.52 (-2.19, 87.35	indirect	
	4.83 (1.07, 9.66	network	
F vs S	-40.73 (-99.91, -6	direct	0.382875
	11.3 (-103.64, 126.93	indirect	
	-23.59 (-91.63, -0.47	network	

D, Digoxin; DF, Digoxin and Flecainide; DS, Digoxin and Sotalol; F, Flecainide; S, Sotalol.

Table S5. Nodesplit analysis of inconsistency of Hydrops group

Comparison	OR(95% Cl)	Effect	P value
D vs DF	8.21 (3.64, 14.87	direct	0.1579
	33.32 (4.71, 99.28	indirect	
	6.02 (2.52, 10.69	network	
D vs DS	0.01 (-5.17, 4.51	network	0.979025
	0.26 (-8.51, 8.38	indirect	
	0.4 (-6.86, 7.69	direct	
D vs F	3.65 (0.92, 6.99	network	0.012125
	4.63 (1.43, 8.62	direct	
	-39.78 (-116.33, 0.03	indirect	
D vs S	-10.65 (-39.99, 6.23	indirect	0.222825
	1.42 (-2.6, 5.75	network	
	1.51 (-3.37, 6.67	direct	
DF vs F	-2.72 (-9.39, 2.35	direct	0.93735
	-3.11 (-10.96, 3.72	indirect	
	-2.34 (-6.92, 1.42	network	
DF vs S	-4.57 (-10.33, 0.27	network	0.000325
	43.05 (2.41, 147.51	direct	
	-9.42 (-18.52, -3.55	indirect	
DS vs S	0.87 (-3.54, 5.73	direct	0.07115
	44.05 (-0.06, 142.01	indirect	
	1.44 (-2.41, 6.12	network	
F vs DS	-2.03 (-10.59, 5.88	indirect	0.017475
	-3.62 (-9.55, 0.92	network	
	-45.33 (-92.95, -7.01	direct	
F vs S	-2.19 (-7.04, 2.21	network	0.3767
	-4.93 (-13.06, 1.04	indirect	
	-16.81 (-82.59, 1.67	direct	

D, Digoxin; DF, Digoxin and Flecainide; DS, Digoxin and Sotalol; F, Flecainide; S, Sotalol.

Table S6. Nodesplit analysis of inconsistency of Non-hydrops group

Comparison	OR(95% Cl)	Effect	P value

D vs DS	0.22 (-3.01, 3.43)	direct	0.705725
	0.27 (-2.29, 3.2)	network	
	-0.93 (-6.5, 5.4)	indirect	
D vs S	1.34 (-1.87, 5.45)	direct	0.5267
	3.54 (-2.8, 10.52)	indirect	
	1.79 (-0.52, 5.07)	network	
DF vs DS	-4.74 (-9.9, -0.84)	network	0.001875
	-36.84 (-127.41, -7.66)	indirect	
	0.15 (-5.91, 6.35)	direct	
DF vs F	0.18 (-4.89, 5.3)	indirect	0.02165
	-26.49 (-76.27, -2.3)	direct	
	-2.43 (-7.38, 1.28)	network	
DS vs S	-0.53 (-8.94, 7.6)	indirect	0.531475
	2.04 (-1.14, 5.58)	direct	
	1.54 (-0.84, 4.47)	network	
F vs DS	-16.84 (-51.97, 1.56)	direct	0.187125
	-2.23 (-6.3, 2.29)	indirect	
	-2.26 (-6.01, 1.14)	network	
F vs S	-1.51 (-8.14, 3.49)	direct	0.5174
	-0.71 (-4.15, 2.86)	network	
	0.64 (-3.99, 6.53)	indirect	

D, Digoxin; DF, Digoxin and Flecainide; DS, Digoxin and Sotalol; F, Flecainide; S, Sotalol.

Table 7. Nodesplit analysis of inconsistency of Death of Total group

Comparison	OR(95% Cl)	Effect	P value
D vs DF	1.91 (-1.89, 6.57)	direct	0.16855
	-11.17 (-44.25, 4.82)	indirect	
	0.84 (-2.2, 3.95)	network	
D vs DS	-0.35 (-3.45, 2.37)	network	0.0317
	-25.46 (-62.29, -1.4)	indirect	
	0.69 (-3.5, 3.95)	direct	
D vs F	0.9 (-1.65, 3.62)	network	0.19635
	0.02 (-2.9, 3.02)	direct	
	10.77 (-3.73, 44.11)	indirect	
D vs S	-1.74 (-7.85, 3.92)	indirect	0.84645
	-1.14 (-4.52, 1.83)	network	
	-1 (-5.89, 2.85)	direct	
DF vs DS	-1.19 (-5.35, 2.51)	network	0.06385
	-32.62 (-93.77, -0.03)	direct	
	-0.15 (-4.96, 4.43)	indirect	
DF vs F	2.64 (-1.76, 8.47)	direct	0.068425
	-3.86 (-10.11, 1.54)	indirect	
	0.05 (-3.23, 3.55)	network	

DF vs S	-1.97 (-6.34, 1.95)	network	0.6483
	-2.07 (-6.77, 2.21)	indirect	
	-16.84 (-96.4, 47.81)	direct	
F vs DS	-1.33 (-5.74, 2.52)	direct	0.870975
	-0.82 (-7.35, 4.4)	indirect	
	-1.26 (-4.92, 1.89)	network	
F vs S	0.52 (-4.5, 4.52)	indirect	0.0205
	-2.04 (-6.03, 1.37)	network	
	-29.83 (-85.97, -2.2)	direct	

D, Digoxin; DF, Digoxin and Flecainide; DS, Digoxin and Sotalol; F, Flecainide; S, Sotalol.

Table S8. Nodesplit analysis of inconsistency of Safety Index group

Comparison	OR(95% Cl)	Effect	P value
D vs DF	10.22 (-0.44, 40.67)	direct	0.013875
	-24.36 (-71, 1.98)	indirect	
	0.38 (-4.08, 4.86)	network	
D vs DS	-2.54 (-7.32, 1.37)	network	0.084175
	-2.4 (-10.85, 5.83)	indirect	
	-31.7 (-105.97, -2.32)	direct	
D vs F	0.47 (-2.95, 3.88)	network	0.01725
	-1.05 (-5.03, 2.61)	direct	
	42.42 (3.26, 104.24)	indirect	
D vs S	-31.02 (-90.15, -3.3)	indirect	0.012675
	-0.88 (-4.74, 2.65)	network	
	0.97 (-3.34, 4.9)	direct	
DF vs DS	-2.96 (-9.07, 2.48)	network	0.109325
	-34.04 (-107.63, 0.6)	direct	
	-1.53 (-9.07, 6)	indirect	
DF vs F	33.27 (6.99, 72.55)	direct	NA
	-41.04 (-104.12, -9.8)	indirect	
	0.02 (-4.43, 4.87)	network	
DF vs S	-1.28 (-6.76, 4)	network	0.7809
	-1.79 (-8.01, 4.03)	indirect	
	-12.78 (-102.85, 58.19)	direct	
F vs DS	-63.16 (-156.81, -14.5)	direct	0.00055
	1.43 (-5.48, 6.57)	indirect	
	-3.02 (-8.37, 1.57)	network	
F vs S	-0.69 (-8.11, 5.8)	indirect	0.74505
	-1.35 (-5.91, 2.93)	network	
	-2.14 (-8.2, 3.5)	direct	

D, Digoxin; DF, Digoxin and Flecainide; DS, Digoxin and Sotalol; F, Flecainide; S, Sotalol.

Table S9. Quality Assessment by NOS

Author	Year	Representativeness of the exposed cohort	Selection of the non exposed cohort	Ascertainment of exposure	Demonstration that outcome of interest was not present at start of study	Comparability of cohorts on the basis of the design or analysis	Assessment of outcome	Was follow-up long enough for outcomes to occur	Adequacy of follow up of cohorts	Sum
van Engelen, A. D.	1994	1	1	1	1	2	1	1	1（−3y）	9
Frohn-Mulder, I. M.	1995	1	1	1	1	1	1	1	0	7
Naumburg, E.	1997	1	1	1	1	1	1	1	1（−7y）	8
Oudijk, M. A.	2000	1	1	1	1	2	1	1	1（−1y）	9
Lisowski, L. A.	2000	1	1	1	1	2	1	1	1（6m−10y）	9
Ebenroth, E. S.	2001	1	1	1	1	1	1	1	0 unclear	7
Krapp, M.	2002	1	1	1	1	2	1	1	1（−2y）	9

Jouannic, J. M.	2002	1	1	1	1	1	1	1	1（-1y）	8
Jouannic, J. M.	2003	1	1	1	1	1	1	1	1（7m-4y）	8
Boldt, T.	2003	1	1	1	1	2	1	1	1 (5y(median))	9
Oudijk, Martijn A.	2003	1	1	1	1	2	1	1	1（6m-3y）	9
D'Alto, M.	2006	1	1	1	1	1	1	1	1（2y-4y）	8
Pezard, P. G.	2008	1	1	1	1	1	1	1	0（2m-17y）	7
Rajka Lulic Jurjevic	2009	1	1	1	1	1	1	1	0 (0.05y-7y)	7
Hahurij, N. D.	2011	1	1	1	1	1	1	1	1（6m-15y（	8
Shah, A.	2012	1	1	1	1	2	1	1	0	8
Uzun, O.	2012	1	1	1	1	1	1	1	1（1y-9y）	8

van der Heijden, L. B.	2013	1	1	1	1	2	1	1	1（1.5y-9y）	9
Ekman-Joelsson, B. M.	2015	1	1	1	1	1	1	1	0（0.2y-22y）	7
Sridharan, S.	2016	1	1	1	1	1	1	1	0	7
Strizek, B.	2016	1	1	1	1	1	1	1	0 (unclear)	7
Ekiz, A.	2018	1	1	1	1	1	1	1	1（6m-4y）	8
Karmegeraj, B.	2018	1	1	1	1	1	1	1	0（1m-5y）	7
Miyoshi, T.	2019	1	1	1	1	2	1	1	0（1m）	8
Tunca Sahin, G.	2021	1	1	1	1	1	1	1	1（0.6y-16y）	8
O'Leary, E. T.	2021	1	1	1	1	1	1	1	1 (0.02m-16.6m)	8

Table S10. Data extraction of included studies in Total group:

Author	Year	Treatment for total	Total for total	Cardioversion for total	Death for total
van Engelen, A. D.	1994	D	24	11	1
		F	10	7	0
Frohn-Mulder, I. M.	1995	D	28	13	NA
		F	7	3	NA
		DF	4	4	NA
Naumburg, E.	1997	D	10	4	0
		DF	1	0	0
		DV	4	0	0
Lisowski, L. A.	2000	D	21	16	1
		DS	5	4	0
		S	9	8	1
Oudijk, M. A.	2000	DS	7	5	1
		S	12	9	3
Ebenroth, E. S.	2001	D	37	17	1
		DF	13	12	0
		FS	1	1	0
Jouannic, J. M.	2002	A	5	2	0
		D	32	26	1
		DA	1	1	0
		DS	1	1	0
		S	1	1	0
Krapp, M.	2002	D	13	4	0
		DF	7	7	0
Boldt, T.	2003	D	26	14	NA
		DF	3	2	NA
		DS	3	2	NA
Jouannic, J. M.	2003	A	4	2	1
		D	7	0	0
		DS	2	0	0
		F	12	7	1

Oudijk, Martijn A.	2003	DS	2	0	0
		S	7	7	1
D'Alto, M.	2008	D	6	5	0
		DF	6	5	1
		DS	2	2	0
Pezard, P. G.	2008	D	16	7	NA
		F	2	1	NA
Lulic Jurjevic, R.	2009	D	2	2	0
		DF	1	1	0
		DS	2	2	0
		F	6	3	3
Hahurij, N. D.	2010	D	8	5	0
		DF	2	1	0
		F	4	3	0
		S	5	4	0
Shah, A.	2012	DS	12	9	3
		S	9	8	0
van der Heijden, L. B.	2012	DS	3	1	0
		F	2	2	0
		S	22	22	0
		FS	3	3	0
Uzun, O.	2012	D	6	0	0
		DF	21	16	1
		F	1	0	0
Ekman-Joelsson, B. M.	2015	D	46	19	1
		DS	32	14	5
		F	2	2	0
		S	32	19	0
Sridharan, S.	2016	D	29	23	NA
		F	27	26	NA
Strizek, B.	2016	D	14	2	0
		DF	6	6	0

		F	28	21	1
Ekiz, A.	2017	DF	1	1	0
		DFS	1	0	1
		F	16	15	1
Karmegeraj, B.	2018	D	3	3	0
		DF	6	5	1
Miyoshi, T.	2019	D	42	25	0
		DS	3	1	0
		S	4	4	0
O'Leary, E. T.	2020	D	35	25	NA
		F	3	2	NA
		S	1	0	NA
Tunca Sahin, G.	2021	DF	12	9	0
		F	1	0	0

D, Digoxin; DF, Digoxin and Flecainide; DS, Digoxin and Sotalol; F, Flecainide; S, Sotalol; DV, Digoxin and Verapamil; DA, Digoxin and Amiodarone; A, Amiodarone; DFS, Digoxin, Flecainide and Sotalol; SF, Sotalol and Flecainide

Table S11. Data extraction of included studies in SVT group

Author	Year	Treatment for SVT	Total for SVT	Cardioversion for SVT
I. M. FROHN-MULDER	1995	D	22	12
		DF	4	4
E.Naumburg	1997	D	4	1
		DF	1	0
		DV	2	0
E.S. Ebenroth	2001	D	37	17
		DF	13	12
		SF	1	1
J.M. Jouannic	2002	A	5	2
		D	32	26
		DA	1	1

		DS	1	1
		S	1	1
M.Krapp	2002	D	13	4
		DF	7	7
		D	26	14
Boldt	2003	DS	3	2
		DF	3	2
		A	4	2
Jean-Marie Jouannic	2003	D	7	0
		DS	2	0
		F	12	7
Martjin A. Oudijk	2003	DS	2	2
		S	7	6
		D	4	4
Michele D’Alto	2008	DS	2	2
		FD	3	3
Pézard	2008	D	8	7
		F	1	0
		D	2	2
Raika LuRajka Lulic ´ Jurjevic	2009	DF	1	1
		DS	2	2
		F	6	3
		D	7	2
Nathan D. Hahurij	2010	F	3	3
		FD	2	1
		S	4	3
Amee Shah	2012	S	4	3
		SD	12	9
		F	2	2
L. B. VAN DER HEIJDEN	2012	S	14	14
		SD	1	1
		SF	3	3

Orhan Uzun	2012	D	6	0
		F	1	0
		FD	15	14
Ekman-Joelsson	2015	D	30	10
		DS	28	13
		F	2	2
		S	25	15
Shankar Sridharan	2016	D	50	31
		F	34	33
Ali Ekiz	2017	DF	1	1
		F	16	15
		FDS	1	0
Balaganesh Karmegeraj	2018	D	3	3
		DF	6	5
Takekazu Miyoshi	2019	D	15	7
		DS	2	1
		S	4	4
Edward T. O'Leary	2020	D	35	25
		F	3	2
		S	1	0

D, Digoxin; DF, Digoxin and Flecainide; DS, Digoxin and Sotalol; F, Flecainide; S, Sotalol; DV, Digoxin and Verapamil; DA, Digoxin and Amiodarone; A, Amiodarone; DFS, Digoxin, Flecainide and Sotalol; SF, Sotalol and Flecainide

Table S12. Data extraction of included studies in AF group

Author	Year	Treatment for AF	Total for AF	Cardioversion for AF
E.Naumburg	1997	D	6	3
		DV	2	0
Lukas A. Lisowski	2000	D	21	16
		DS	5	4
		S	9	8
Martjin A. Oudijk	2003	DS	2	0
		S	7	7

Nathan D. Hahurij	2010	D	1	1
		F	1	1
		S	1	0
L. B. VAN DER HEIJDEN	2012	S	8	8
		SD	2	0
Ekman-Joelsson	2015	D	16	9
		DS	4	1
		S	7	4
Balaganesh Karmegeraj	2018	D	2	2
		DF	3	3
		DS	3	3
Takekazu Miyoshi	2019	D	27	16
		D	11	8
		D	2	1
		DS	1	0
Edward T. O'Leary	2020	D	6	2
		F	3	3
		S	2	2
Gulhan Tunca Sahin	2021	DF	12	9
		F	1	0

D, Digoxin; DF, Digoxin and Flecainide; DS, Digoxin and Sotalol; F, Flecainide; S, Sotalol; DV, Digoxin and Verapamil; DA, Digoxin and Amiodarone; A, Amiodarone; DFS, Digoxin, Flecainide and Sotalol; SF, Sotalol and Flecainide

Table S13. Data extraction of included studies in Hydrops group

Author	Year	Treatment for hydrops	Total for hydrops	Cardioversion for hydrops
van Engelen	1994	D	10	1
		F	5	2
I. M. FROHN-MULDER	1995	D	6	1
		F	7	3
Lukas A. Lisowski	2000	D	16	11
		S	1	0
Martjin A. Oudijk	2000	DS	4	3

		S	4	2
M.Krapp	2002	D	10	1
		DF	6	6
Jean-Marie Jouannic	2003	A	4	2
		D	7	0
		DS	2	0
		F	12	7
Michele D’Alto	2008	D	2	1
		DF	3	2
Pézard	2008	D	4	1
		F	1	0
Nathan D. Hahurij	2010	D	2	0
		DF	2	1
		F	2	2
		S	3	3
Boldt	2003	D	6	0
		DF	1	1
L. B. VAN DER HEIJDEN	2012	F	2	2
		S	4	4
		SF	2	2
Ekman-Joelsson	2015	D	17	4
		DS	19	6
		F	1	1
		S	14	6
Sridharan	2016	D	21	9
		F	7	7
Strizek	2016	D	3	0
		DF	3	3
		F	18	12
Ali Ekiz	2017	DF	1	1
		DFS	1	0
		F	14	13

Takekazu Miyoshi	2019	DS	3	1
		S	1	1
Gulhan Tunca Sahin	2021	DF	5	4
		F	1	0

D, Digoxin; DF, Digoxin and Flecainide; DS, Digoxin and Sotalol; F, Flecainide; S, Sotalol; DV, Digoxin and Verapamil; DA, Digoxin and Amiodarone; A, Amiodarone; DFS, Digoxin, Flecainide and Sotalol; SF, Sotalol and Flecainide

Table S14. Data extraction of included studies in Non-hydrops group

Author	Year	Treatment for non-hydrops	Total for non-hydrops	Cardioversion for non-hydrops
van Engelen	1994	D	14	10
		F	5	5
I. M. FROHN-MULDER	1995	D	22	12
		DF	4	4
Lukas A. Lisowski	2000	D	5	5
		DS	5	4
		S	8	8
Martjin A. Oudijk	2000	DS	3	2
		S	8	7
J.M. Jouannic	2002	A	5	2
		D	32	26
		DA	1	1
		DS	1	1
		S	1	1
M.Krapp	2002	D	3	3
		DF	1	1
Boldt	2003	D	26	14
		DF	3	2
		DS	3	2
Michele D’Alto	2008	D	4	4
		DF	3	3
		DS	2	2
Pézard	2008	D	12	6

		F	1	1
Raika LuRajka Lulic ´ Jurjevic	2009	D	2	2
		DF	1	1
		DS	2	2
Nathan D. Hahurij	2010	D	6	5
		F	2	1
		S	2	1
Amee Shah	2012	DS	4	4
		S	9	8
L. B. VAN DER HEIJDEN	2012	DS	3	1
		S	18	18
		SF	1	1
Orhan Uzun	2012	D	6	0
		DF	13	11
		F	1	0
Ekman-Joelsson	2015	D	29	15
		DS	13	8
		F	1	1
		S	18	13
Shankar Sridharan	2016	D	29	23
		F	27	26
Strizek	2016	D	11	2
		DF	3	3
		F	10	9
Takekazu Miyoshi	2019	D	42	25
		S	3	3

D, Digoxin; DF, Digoxin and Flecainide; DS, Digoxin and Sotalol; F, Flecainide; S, Sotalol; DV, Digoxin and Verapamil; DA, Digoxin and Amiodarone; A, Amiodarone; DFS, Digoxin, Flecainide and Sotalol; SF, Sotalol and Flecainide

Table S15. Safety data extraction of included studies:

Author	Year	Dosage	SNeonatal and follow-up adverse events	Maternal side effects	Structural heart disease
van Engelen , A. D.	1994	Initially treated by maternally administered **digoxin** (intravenous loading dosage of 1.5 mg/day, given in three parts, followed by an oral maintenance of 0.5 to 0.75 mg/day) (n=14) or **flecainide** (twice a day 100 to 150 mg orally) (n=5).	➢ In the group of patients with SVT, a **reentry mechanism** could be seen on the electrocardiogram in **8** (**4 Wolff-Parkinson-White syndrome**, of whom **2 had no more tachycardia after birth**; **4 permanent junctional reciprocating tachycardias**).<br>➢ **At age of 1 month, 78%** of the patients with a history of fetal **SVT or AF** were **receiving antiarrhythmic drugs**, either for **recurrent tachycardia** or as **antiarrhythmic prophylaxis.**<br>➢ **At 3 years**, **14%** were **taking antiarrhythmic drugs.** The 2 patients with **permanent junctional reciprocating tachycardia** in this group of 22 were still receiving medication at the age of 3 years.	**2 of the 15** mothers receiving **flecainide** had **blurred vision and dizziness** that resolved after the dose decreased.	Unmentioned
Frohn-Mulder, I. M.	1995	**Digoxin** was the first drug of choice. After maternal administration of a loading dose of 1 mg, a maintenance dose of 0 2 5 mg, three times a day, was given. Serum drug concentrations were measured and the dosage adjusted to maintain concentrations around 2 ng/ml.<br>**Flecainide** was administeredorally 3 times 100 mg a day; the dosage was adjusted if necessary to keep serum drug concentrations between 0.5-1 microgram/milliliter (mcg/ml).	➢ Postnatal treatment was necessary in 19 infants. In **17 infants digoxin alone was sufficient to control normal rhythm**.<br>➢ In 2 infants with SVT **flecainide was given after digoxin failure**. These 2 infants had also not reacted to digoxin prenatally. Postnatal treatment was discontinued after 1 week in 1 case because of **bradycardia**.<br>➢ In all other cases, therapy was discontinued after 1 year, without recurrence of SVT. Of the **7 newborn which presented with hydrops, all but one needed treatment.** 2 infants remained in sinus rate by digoxin alone, in one case propranolol was added. In 2 infants, flecainide was continued. In these infants therapy could be withdrawn after one year without recurrence of SVT. In another infant, which applied to have an **ectopic atrial tachycardia** and was difficult to manage prenatally, control of heart rate was finally achieved by **sotacor**. **When this medication was withdrawn after 2 years there was a recurrence of SVT within 6 months, necessitating further sotacor medication**. **1 infant died on the second day after delivery because of complications of ongoing tachycardia and severe hydrops at 31 weeks of gestation**.	Unmentioned	An atrial septal aneurysm was established in 3 fetuses.

Naumburg, E.	1997	0.5-1.25 mg/day, oral or intravenously, b.i.d. for **Digoxin**	➢ One infant with WPW syndrome was treated with verapamil as monotherapy due to insufficient effect of digoxin.<br>➢ One infant with an intrauterine tachycardia diagnosed as FSVT, which resolved spontaneously, developed tachycardia with an atrial flutter at 6 months of age. She needed an **electric cardioversion** and was then treated with digoxin for 6 months.<br>➢ Another child with **antenatal atrial flutter/fibrillation**, who received digoxin for 7 months after birth, developed **paroxysmal tachycardia** at 2 years of age. The tachycardia resolved by a vagal maneuver.<br>➢ A 3rd child with antenatal FSVT treated with digoxin during his 1st year of life due to a WPW syndrome developed tachycardia at 7 years of age. The tachycardia resolved spontaneously.	Nothing serious apart from nausea	Excluded
Lisowski, L. A.	2000	Detailed protocols were described from case to case.	➢ Of the original 45 patients, 2 patients died in utero, and 12 of the 43 live-born infants were in **AF at birth**.<br>➢ 3 infants were in serious trouble with **poor Apgar scores** at birth. In four of the 45 patients, including the one described above, **neurological morbidity was documented immediately postnatally**, suggesting an association with the prenatally existing arrhythmia. The neurological damage ranged from **severe cerebral hypoxicischemic lesions to intraventricular hemorrhage, resulting in a hydrocephalus**.<br>➢ One patient had a small periventricular infarction that is resolving.	Unmentioned	Excluded
Oudijk, M. A.	2000	The starting dosage used was 80 to 160 mg of **sotalol**, given orally 2 times a day. The dosage was occasionally increased to a maximum of 160 mg 3 times per day if tachycardia persisted. **Digoxin** was added to the treatment in patients in whom adequate control could not be achieved with sotalol as a single therapy.	➢ 4 intrauterine deaths occurred<br>➢ No rhythm disturbances were seen in 11 of the 17 surviving patients with fetal tachyarrhythmias (65%). Prophylactic drug therapy was administered for 9 months to 1 year in 5 of these 11 patients; 2 patients received sotalol, and the other 3 patients received digoxin. A relapse of tachycardia was seen in 6 of the 17 cases (35%). Two patients had AF and 3 had SVT. The child with VT had recurrent VT after birth. Two patients were successfully treated with sotalol, 1 patient was treated with digoxin, and 1 patient received a combination of sotalol and digoxin. The fifth patient had AF and was electrically cardioverted to restore sinus rhythm,; sotalol was also administered.<br>➢ All newborns were treated until the age of 1 year. 2 patients with fetal hydrops had	In 2 cases, maternal adverse effects were encountered. They were only temporary.	Excluded

			significant **neurological morbidity** immediately after birth. One had SVT and was treated with sotalol, the second had AF and was treated with sotalol and digoxin. Before conversion to persistent sinus rhythm was achieved, these patients experienced intermittent episodes of tachycardia with long-lasting periods of normal sinus rhythm. These episodes lasted 10 and 21 days, respectively, at the gestational ages of 29 and 25 weeks, respectively. Although control of the tachycardia was achieved and these babies were born with good Apgar scores, their post- natal evaluation showed neurologic pathology; this was due to **intracranial hemorrhage** in one and **cerebral hypoxic ischemia** in the other.		

Ebenroth, E. S.	2001	**Digoxin** at 1-1.5 mg, either i.v. or PO based on physician preference, over 24 hours and they were started on a maintenance dose of 0.25 to 1 mg/day. Doses were adjusted to attain high therapeutic levels of 1.4-2.0 ng/ml whenever possible. All patients since 1990 have received flecainide. **Flecainide** was administered to the mother in an oral dose of 100-500 mg/day divided b.i.d. to t.i.d. and titrated to obtain therapeutic maternal drug levels. Flecainide levels were obtained when doses greater than 300 mg/day were necessary.	➢ The only patient with twins in our study developed **HELLP syndrome** at 34 weeks gestation, and the infants were delivered. Infant A was born with a **stable wide complex tachycardia**, whereas infant B, the one being treated for fetal tachycardia, had markedly **prolonged QRS duration**. Flecainide levels sent from cord blood revealed high therapeutic values of 1.0 and 0.78, respectively. Both infants did well, and after 2 days off flecainide, their electrocardiograms had normalized. Twin B then developed recurrent SVT.<br>➢ There were three postnatal deaths in this population of patients. The first was a patient with **diaphragmatic hernia and hydrops at presentation**. The child was converted with digoxin alone and born at term in sinus rhythm. The second was the other patient with **diaphragmatic hernia**, who was also converted on digoxin alone, and who remained in sinus rhythm until birth at term. Both of these infants died from complications of their **diaphragmatic hernias** within the first 2 hours of life. The third was the patient mentioned previously, who presented at 19 weeks gestation in SVT with **hydrops and oligohydramnios**. He was converted with digoxin and flecainide and was delivered at 29 weeks gestation in sinus rhythm. He subsequently died at 18 days of age following an episode of **necrotizing enterocolitis, sepsis, and multisystem organ failure**. In all three cases, hydrops had resolved, and sinus rhythm had been restored prenatally.	In the digoxin group, one woman suffered from **transient Mobitz type II second-degree atrioventricular block** on a therapeutic dose of digoxin. Women in the flecainide group demonstrated mild to **moderate prolongation of QRS durations**, but none developed any significant dysrrhythmia nor experienced any adverse effects. There was only one delivery prior to 34 weeks gestational age. This fetus presented at 19 weeks gestational age in SVT with **hydrops and oligohydramnios**. Following conversion with digoxin and flecainide, she was discharged. She returned one week later with **vaginal bleeding** and had persistent vaginal bleeding for the next 8 weeks until preterm labor and delivery occurred.	38/40 fetuses possessed structurally normal hearts. 1 suffered from tuberous sclerosis with multiple rhabdomyomas ; the other had a dilated cardiomyopathy that was postnatally diagnosed as mild Ebstein's anomaly. Two of the fetuses were known to have left-sided diaphragmatic hernias prior to the diagnosis of tachycardia.
Jouannic, J. M.	2002	**Digoxin** was started at a dose of 0.25 mg three times daily and the dose was adjusted to achieve a maternal serum concentration within the therapeutic range (1.5–2 ng/ml). Flecainide was given at a	➢ In group A (no tricuspid regurgitation, n = 30), One of the 3 fetuses in whom digoxin failed died in utero at 28 weeks, 8 days after starting treatment. In another case, digoxin was replaced by flecainide after 10 days, because **ascites and pericardial effusion** appeared. Although conversion to sinus rhythm was not achieved, the fetal heart rate decreased under flecainide, and **hydrops** improved before birth. In the last case, digoxin was continued despite persistence of the arrhythmia. A Caesarean section was performed	Unmentioned	The cardiac structure was normal in all but 1 case in which a **ventricular**

		dose of 100 mg three times daily. Sotalol was given at a dose ranging from 160 to 240 mg/day.	at 36 weeks for prolonged rupture of membranes. This child was converted to sinus rhythm by postnatal amiodarone therapy. The first-line treatment of the remaining 5 children in group A was digoxin and amiodarone in 1, digoxin and sotalol in 1, sotalol in 1 and amiodarone in 2. All of them were converted prenatally.<br>➢ In group B (tricuspid regurgitation, n = 10), in 1 case, hydrops resolved 15 days after digoxin had been added to amiodarone but sinus rhythm could not be achieved before birth. In the other case, hydrops increased and the baby was delivered by Caesarean section at 36 weeks. No further follow-up were described.		**septal defect** was diagnosed prenatally and repaired at 8 months of age
Krapp, M.	2002	Drug therapy was continued until delivery. **β-methyldigoxin** was used as first line treatment in all cases (Table 1). After 3 days, the intravenous loading dose of 800–1000 µg per day was followed by an oral maintenance dose of 500–600 µg per day. Serum digoxin levels were maintained between 1.5 and 2.5 ng /mL. When supraventricular tachycardia persisted longer than 48–72 h after the initiation of drug therapy, in the first 16 months of this study, **verapamil** was added at an oral dose of 80 mg four to six times daily. **Flecainide** was always combined with β-methyldigoxin and used as first line treatment, as second line treatment, or as third line treatment replacing verapamil when fetal supraventricular tachycardia persisted over 72 h. The dose was 100 mg three to four times daily orally, while serum	➢ Two newborns with sinus rhythm were prophylactically kept on digoxin, which was arbitrarily terminated after 4 and 8 weeks, respectively.<br>➢ Six newborns had recurrence of SVT and were treated with antiarrhythmic drugs. One newborn was treated with digoxin alone. In two cases, propafenone alone and, in one case, propafenone in combination with digoxin was used. Another two cases were treated with propranolol alone and in combination with digoxin, respectively.<br>➢ Nineteen of 20 infants showed normal development after 2 years. However, one infant (Case 11), who was delivered at 33 + 1 weeks of gestation after conversion to sinus rhythm in hydropic condition because of preterm premature rupture of membranes, had multiple **disseminated skin lesions** (blueberry muffin eruptions) at birth suspicious for extramedullary erythropoiesis. The Apgar scores were 5, 7, and 8 after 1, 5 and 10 min, respectively. Arterial blood pH (7.31) and hemoglobin (114 g / L) were within normal limits. At the age of 2 years, there was mild periventricular leukomalacia verified by magnetic resonance imaging. A short episode of tachycardia was controlled by propafenone during the neonatal period. The infant showed normal motoricity, but bilateral dysacousia and disturbed development of speech.	There were no maternal side-effects during flecainide treatment.	Excluded

		flecainide levels were maintained between 500 and 1000 µg / L.			
Boldt, T.	2003	**Digoxin** treatment was provided orally at a dosage of 0.25 mg two or three times daily, with the dosage adjusted to achieve a maternal serum concentration in the therapeutic range (1.5 to 2.0 ng/mL). **Flecainide** was provided orally at a dosage of 100 mg two or	Mentioned but lack of data for drug therapy group was just a part of its analysis.	Mentioned but lack of data for drug therapy group was just a part of its analysis.	Mentioned but lack of specific data for drug therapy group was just a part of its analysis.

		three times a day and amiodarone 150 to 300 mg daily. Direct fetal **amiodarone** therapy (triple-route administration by use of combined direct fetal intravenous, direct fetal peritoneal, and maternal oral amiodarone) consisted of amiodarone on the basis of estimated fetal weight (7.5 mg/kg).			

Jouannic, J. M.	2003	**Digoxin** was started at a dose of 0.25 mg three times daily and the dose was adjusted to achieve a maternal serum concentration within the therapeutic range (0.8 to 2 ng/mL). **Flecainide** was given at a dose of 100 mg three times daily. **Sotalol** was given at a dose range from 160 to 240 mg per day. Following the report byAllan et al. (1991), our policy changed andflecainide was used asfirst-line therapy. Before that report, either digoxin or digoxin + sotalol were used asfirst-line therapy.	➢ In one case, amiodarone therapy was started at 24 weeks in a fetus with major hydrops and poor ventricular systolic function with tricuspid regurgitation. A **TOP** was decided at 26 weeks because of increasing hydrops and no spontaneous fetal movement. In another case, amiodarone was started at 37 weeks leading to a decrease in the fetal heart rate that remained below 220 bpm, but with no conversion to sinus rhythm. This fetus was delivered vaginally 2 weeks later and was converted to sinus rhythm with intravenous digoxin therapy.<br>➢ All live newborns with persistent SVT were converted in the first week of life using either digoxin or amiodarone intravenous therapy. One infant who developed **postnatal hypotonia** associated with growth retardation and inherited mitochondrial cytopathy was diagnosed at the age of four months. Of the 11 live neonates who were treated with amiodarone in the prenatal period, the **thyroid stimulating hormone** (TSH) level was elevated at day 3– 4 in two cases (60.3 and 51.8 mU/L, normal<20 mU/L). Those two neonates received **thyroid hormone** substitution therapy for a period of two and three months, respectively.	Maternal tolerance of anti-arrhythmic drugs was good in all cases but one. Digoxin intoxication occurred in one case revealed by **abdominal pain and vomiting** on day 5. The patient had received amiodarone for five days at a loading dose of 2000 mg per day in association with digoxin 0.5 mg twice daily. Maternal serum digoxin concentration was 3.4 ng/mL. The maternal electrocardiogram showed an **increased PR interval.**	Excluded

Oudijk, Martijn A.	2003	**Sotalol** therapy was initiated at either 80 mg twice daily or 160 mg twice daily, increased to a maximum of 160 mg thrice daily, and the addition of digoxin in the event of conversion to sinus rhythm did not occur (18,22). Patients were regularly scheduled (at least once a week) for control visits to evaluate the fetal heart rhythm and possible signs of congestive heart failure.	➢ Eight infants (5 had AF and 3 had SVT) had no rhythm disturbances during the newborn period, and no medication was initiated.<br>➢ Three patients showed AF at birth (two therapy-resistant cases and one relapse), and all required electrical cardioversion to reach sustained sinus rhythm. These three patients are currently doing well and require no medication.<br>➢ Five patients showed SVT postnatally, of whom two had Wolff-Parkinson-White syndrome, two had persistent junctional reciprocating tachycardia, and one patient showed intermittent periods of SVT of unknown origin. All are doing well on antiarrhythmic therapy, consisting of digoxin in two patients, propranolol in one patient, and a combination of these two agents in two patients.<br>➢ All surviving infants are in good neurologic condition.	Unmentioned	Unmentioned
D'Alto, M.	2008	The starting **digoxin** dosage was 1–2 mg i.v. over 36 h (1/2 dose þ 1/4 dose after 12 h and 1/4 dose after 24 h); maintenance dose: 0.250 mg, 2– 4 times a day orally (mean of 0.012 ± 0.004 mg/kg/day), monitoring serum digoxin level. The starting dosage of **sotalol** was 80– 160 mg, given orally twice a day. This dosage was occasionally increased o a maximum of 160 mg, 3 times a day if tachycardia persisted (mean of 5 ± 2 mg/kg/day). The starting dosage of **flecainide** was 50– 100 mg given orally twice a day, occasionally increased to a maximum of 200 mg twice a day (mean of 3 ± 2 mg/kg/day).	➢ Follow-up was possible in 33 of 36 cases (one intrauterine and two neonatal deaths occurred). Four fetuses with tachycardia had congenital heart disease: one Ebstein anomaly, one aortic coarctation (both prenatally diagnosed), and two atrial septal defects.<br>➢ Twenty out of the 33 surviving patients, (19 with 1 : 1 AV tachycardia and one with prevalent 2 : 1 AV tachycardia), underwent transesophageal electrophysiological (EP) study in washout therapy soon after birth (mean age of 3.5 ± 2.2 days). In one patient with incessant AVRT during fetal life, no tachycardia was induced at postnatal EP study. Prophylactic drug therapy was postnatally administered in 16 patients with spontaneous or EP study-induced tachycardia .<br>➢ None of these patients have shown recurrent signs of tachycardia, and they are currently doing well. The child with LQTS had recurrent VT after birth until he was 3 months old. At 2.8-year follow-up, he is taking propranolol and mexiletine and is asymptomatic and event free. Thirteen patients repeated the EP study in washout at 1 year of life: in four of 13, no tachycardia was induced, and medical therapy was discontinued. At a 3 ± 1.1-year follow-up, 33 out of 35 live-born children of our study were alive and well and none of them suffered from late neurological disease.	No maternal adverse effects were encountered.	2 out of 36 fetuses prenatally showed a congenital heart disease: one **Ebstein anomaly** and one **aortic coarctation**

Pezard, P. G.	2008	**digoxin**, loading dose 1—1.5 mg/day twice daily, maintenance dose 0.5 mg/day.	Of the 21 neonates for whom we have follow-up information (ranging from two months to 17 years), nine infants had recurrence of tachycardia: eight SVT required postnatal maintenance therapy (38% of follow-up), including all four cases that had evidence of **pre-excitation on neonatal electrocardiogram**. The fetus with VT had some runs of VT after birth, with spontaneous recovery at one month of life.	Two patients given the amiodarone—digoxin combination showed elevated levels of serum digoxin (up to 5 ng/ml) and clinical signs of **digitalis intolerance,** leading to the discontinuation of the digoxin treatment and the administration of amiodarone alone.	Unmentioned
Lulic Jurjevic , R.	2009	The protocol for treatment was described in original article in form of flow chart	➢ Long-term antiarrhythmic prophylaxis was administered in 17 patients, 14 of those born in tachycardia, 2 patients in whom tachycardia recurred after birth, and 1 patient with WolffParkinson-White syndrome.<br>➢ The median period of treatment was 12 months, with a range from 0.13 to 52 months, and the median period of follow-up was 3.5 years, with a range from 0.05 to 7 years. The baby born with ventricular tachycardia having trisomy 18 died at the age of 3 weeks. 25 of the cohort have survived over the long-term (86.2%). Excluding the patient with trisomy 18, only patients with fetal hydrops suffered mortality, with 37.5% of this group dying. The difference from the group of non-hydropic fetuses was significant, the value for p equal to 0.03. Among the 5 long-term survivors of those presenting with fetal hydrops, 1 patient has a severe neurological and cognitive handicap. There was no evidence of neurological impairment in any of the long-term survivors from the group presenting with preserved cardiac function.	Unmentioned	small muscular ventricular septal defects in 2, a nonrestrictive perimembranous ventricular septal defect in 1, and Ebstein's malformation in the other.

Hahurij, N. D.	2010	**Digoxin** was administered to the mother in adjusted oral doses to maintain a maternal serum therapeutic level of 1–2 ng/mL (loading dose 2 × 0.75 mg, maintenance 0.25–0.5 mg, maximum 0.75 mg/daily). **Flecainide** (oral dose 200–400 mg daily) and **sotalol** (oral dose 2 × 80–160 mg daily) were used as secondary agents.	➢ The overall incidence of cardiac anomalies in the study population was 18% (8/44).<br>➢ In the SVT group 1 infant had a **ventricular septal defect** and 1 infant had **cardiomyopathy, poly valvular disease and pulmonary stenosis**.<br>➢ In the AF group, 1 infant was found to have **coarctation** of the aorta.<br>➢ In the AVB group, 5 of 9 infants had complex CHD **(congenitally corrected transposition of the great arteries (cc-TGA), n = 2; left atrial isomerism, n = 1; ventricular septal defect, pulmonary stenosis, cardiomyopathy, n = 1; endocardial fibroelastosis, n = 1)**.<br>➢ Postnatally, AVB block remained present in all survivors (n = 6) and 5 patients received pacemaker therapy immediately after birth. In 67% of AF-fetuses and 78% of SVT-fetuses episodes of tachycardia or incessant tachycardia remained present after birth.<br>➢ Nineteen of the 28 children in the SVT group were treated with medication after birth. SVT was self-limiting in 74% (14/19), and treatment could be stopped within thefirst year of life. Five of 28 fetal SVT (AVRT) cases had **WPW-syndrome**, as demonstrated by the presence of ventricular preexcitation on the ECG at birth. In 2 cases, radiofrequency catheter ablation of an accessory pathway was performed in the first months of life due to drug-refractory tachycardias.<br>➢ AF was treated with anti-arrhythmic therapy (n = 4) or cardioversion (n = 2). After initial conversion to sinus rhythm, AF did not recur in all 6 cases. Interestingly, in two AF cases the presence of an accessory pathway was demonstrated. One AF case showed **WPW-syndrome** on ECG after cardioversion, another AF case developed recurrent AVRT requiring anti-arrhythmic therapy.	Unmentioned	8 had complex congenital heart malformations

Shah, A.	2012	**Digoxin**: confused reported<br><br>The maximum **sotalol** dose among the treated pregnant women ranged from 80 mg 2 times/day to 240 mg 3 times/day.	➢ 8 treated fetuses had documented arrhythmias postnatally. Three of these infants were rapid responders in utero. The SVT mechanism was correctly predicted by the VA and AV interval assessment, with 1 infant with permanent junctional reciprocating tachycardia (long VA in utero), 1 with ectopic atrial tachycardia (long VA in utero), and 1 with AV node reentry tachycardia (short VA in utero). All 5 live-born fetuses with more difficult to treat SVT in utero (slow or partial responders) have had SVT that has been a challenge to treat after birth: 4 had AV reentry tachycardia (1 with Wolff-Parkinson-White syndrome and the others with concealed pathways), and 1 had permanent junctional reciprocating tachycardia. No fetus with AF had tachycardia postnatally.	The maternal symptoms during sotalol therapy included nausea, dizziness, and fatigue in 4 mothers who were concomitantly taking digoxin. No mothers developed torsades during sotalol therapy. In no cases was sotalol discontinued because of maternal side effects.	Mentioned but lack of data.
van der Heijden, L. B.	2012	The initial dosage of **sotalol** was 160– 320 mg daily in two to three doses. The dosage of **digoxin** was 0.375– 1.000 mg daily in two to four doses. The initial dosage offlecainide was 100– 150 mg twice daily. The maximum dosage of **flecainide** was 400 mg daily.	➢ In the two patients without SR directly after birth (7%), rate control was achieved antenatally and the postpartum ECG showed AF. Electrical cardioversion established normal SR in one of these patients, and a maintenance dose of sotalol was discontinued after 3 months. In the other patient, several attempts to convert electrically failed, and a maintenance therapy of digoxin and sotalol was initiated. The patient converted within a few days. Digoxin was discontinued 2 months after birth and sotalol 8 months after birth. No recurrent tachycardias were detected in either case.<br>➢ In eight of the 26 infants with SR directly after birth (31%), SVT was observed 5 hours to 3 weeks postnatally. In the remaining 18 patients (69%; 11 with fetal SVT, seven with fetal AF) no rhythm disturbances were detected after birth and there were no events during follow-up (median, 54 (range, 19– 102) months). Five of the eight patients with recurrent atrial tachycardia showed SVT within 3 days after birth (63%). The antenatal rhythm diagnosis was concordant with the postnatal rhythm diagnosis in four of these patients (SVT) and discordant in one (with antenatal rhythm diagnosis of AF and postnatally diagnosis of SVT). One of these five children (antenatal diagnosis concordant with postnatal diagnosis) was diagnosed with atrioventricular re-entry tachycardia based on Wolff−Parkinson−White syndrome. This child had one relapse at the age of 3 months. A maintenance dose of sotalol therapy was stopped 15 months after birth without recurrence of tachycardia (follow-up at 31 months). Four patients received sotalol in a maintenance dose for 8– 12 months, after which treatment was discontinued successfully without recurrent signs of tachycardia. Three of the eight patients developed atrial tachycardia 2– 3 weeks after birth (37%). One was diagnosed with ectopic atrial tachycardia and at the time of writing is doing well on antiarrhythmic therapy with sotalol (last follow-up at 46 weeks). In the other two patients, a maintenance dose of sotalol was	Maternal adverse effects of sotalol therapy were encountered in 15 cases and consisted of **dizziness (n = 11), fatigue (n = 4), nausea and vomiting (n = 3)**, **headache (n = 3) and dyspnea (n = 1)**.	Excluded

			discontinued after 12 months. No relapses occurred. Significant neurological morbidity was present after birth in one patient, presenting at 36 + 5 weeks with SVT at 280 bpm with fetal hydrops (ascites, skin edema, pericardial effusion). Antenatal treatment with sotalol achieved SR within 2 days. The infant was born at 37 + 1 weeks' gestation with normal Apgar scores and blood gases after a spontaneous vaginal delivery. SVT occurred 5 hours after birth. Cardioversion with adenosine was successful and maintenance therapy with sotalol was initiated. During postnatal evaluation, magnetic resonance imaging depicted a large infarct of the left medial cerebral artery with hemorrhagic components due to venous thrombosis of the transverse sinus.		
Uzun, O.	2012	D: 19.6±0.92 nmol/L<br>1.94±0.90 nmol/L for D, 0.51±0.19 ±0.19 mg/L for F<br>F: 0.70±0.40 mg/L	➢ A severe vein of Galen aneurysm complicated one case and the patient opted for termination of her pregnancy. There was one neonatal death due to severe pulmonary hypoplasia and gross foetal ascites. In re-entry tachycardia, 1 foetus who did not respond to any treatment antenatally showed sinus rhythm at birth. However, tachycardia recurred a few hours after birth, and adenosine effectively terminated the tachycardia. Sinus rhythm was maintained on amiodarone infusion, but the newborn died due to severe respiratory distress. In all, seven newborns developed atrioventricular re-entry tachycardia beyond 24 hours of age and needed anti-arrhythmic medication.<br>➢ Out of the seven cases of atrial flutter, four neonates were noted to be in atrial flutter at birth, and therefore they were treated with direct current cardioversion to restore the sinus rhythm. Anti-arrhythmic medication was needed in two newborns because of the emergence of atrioventricular re-entry tachycardia– in one patient a few hours after restoration of sinus rhythm by direct current cardioversion, and in the other a week after delivery. A total of nine children– seven with re-entry supraventricular tachycardia and two with atrial flutter– required postnatal anti-arrhythmic treatment. In seven children, anti-arrhythmic medication was discontinued within a year. Only two children remained on anti-arrhythmic medication in the follow-up beyond the first year of age. After birth, three newborns exhibited pre-excitation – Wolff–Parkinson–White Syndrome– two of whom had recurrence of atrioventricular re-entry tachycardia and required treatment for a few months. Both patients remained arrhythmia free after discontinuation of treatment. A third patient has continued to show asymptomatic pre-excitation on a 12-lead electrocardiogram, but never developed arrhythmia following termination of atrial flutter with direct current cardioversion. There is only one child with learning difficulties who also has a ventriculoperitoneal shunt for congenital hydrocephalus. It would	1 maternal **hypothyroidism** but adequately treated; 5 has **nausea, headache, tiredness, and loss of appetit**e. 1 patient complained of **sickness and visual symptoms** related to digoxin despite the dose being in therapeutic range– 1.32 nmol/L. **Deranged liver function test** in 1 patient was caused by obstetric cholestasis. Upon reduction of the offending drug dose, all of the above-mentioned side effects resolved rapidly.	1 has Ebstein's anomoly of the tricuspid valve; Postnatally, 3 were found **small muscular ventricular septal defects** but closed spontaneously in the follow-up; 2 **atrial septal defects** postnatally; 1 **hydrocephalus with aqueduct stenosis and hypoplastic cerebellum**

			be more likely that the neurological abnormalities were related to his hydrocephalus rather than foetal tachycardia.		
Ekman-Joelsson, B. M.	2015	Transplacental digoxin treatment usually started with an oral loading dose of 1.5–2.0 mg given on a 24- to 36-hour basis, followed by a daily maintenance dose of approximately 0.5 mg to obtain a maternal drug level in the upper therapeutic range. Cases with severe hydrops frequently received an intravenous loading dose of 1.0 mg over a 24-hour period, followed by injections of 0.25–0.5 mg on days two and three. Sotalol treatment routinely started with an oral dose of 80 mg twice daily and increased to 160 mg twice daily within three days, depending on the degree of foetal hydrops. In rare cases, it was increased to a maximum dosage of 160 mg three times a day. Flecainide treatment started with a dose of 100 mg two to three times a day and increased to a maximum dosage of 400 mg per day.	➢ Termination of arrhythmia was associated with not having a Caesarean section, a longer time delay from diagnosis to delivery and a later gestational age at delivery. Preterm delivery was observed in 28% of the cases and three-quarters of the 12% delivered before 35 weeks of gestation had a hydropic foetus at presentation. Two-thirds of our foetuses were boys, and the girls had a higher rate of intrauterine conversion (86 versus 63%, $p < 0.05$) and a lower degree of postnatal arrhythmia (24 versus 55%, $p < 0.01$). Neonatal neurological morbidity was observed in three cases. In one case, this was due to a postnatal cerebral haemorrhage, and in the other two cases, this was due to prenatal cerebral infarction, probably secondary to thromboembolism. Postnatal arrhythmia was seen in cases that did not respond to intrauterine treatment and was also seen in 25% of those with cardioversion (Table 4). Electroconversion was used in two cases with AF and one with AVRT. There was a small difference in the time from diagnosis to delivery between patients with AF and AVRT (5.1 ± 4.4 versus 7.5 ± 5.4 weeks, $p < 0.05$), but they were delivered at the same gestational age and all other neonatal outcome measures were similar. Postnatal arrhythmias were observed within two weeks of delivery in 19/38 (50%) cases with AF, 31/85 (36%) with AVRT, 1/3 with AET, 2/4 with PJRT, 1/2 with JET and none of the four cases with CAT or VT. In addition to the two severe cardiac malformations diagnosed prenatally, two small ventricular septal defects and one atrial septal defect were found in another three cases with AF. Postnatal antiarrhythmic treatment was started in 94 (70%) patients and treatment was more frequently provided in cases with foetal hydrops than those who were nonhydropic (87 versus 68%, $p < 0.05$), but there was no difference in frequency between AF and AVRT cases. The duration of treatment was typically six (1–12) months, 11 (8%) still had recurrence of arrhythmia after one year of age and six were treated with radiofrequency ablation. One case with PJRT and poor ventricular function at diagnosis had cardioversion and functional normalisation after birth, but arrhythmia recurred, accompanied by ventricular deterioration, and a cardiac transplant was carried out at eight years of age. Neurological sequelae were present infive cases. Two of the three with neurological symptoms at birth had hemiparesis, but no sequela was documented in the third. Another two children had a mild neurodevelopmental delay and afifth had an unclear neurological disease, with paraparesis, and died atfive months of age. Tumours were diagnosed in two infants and	Side effects were reported by 21 of the 99 women who received antiarrhythmic drugs: ten who were prescribed digoxin, three who used sotalol and eight who used a combination of both. In two cases, preeclampsia with decreased renal function and severe digoxin intoxication developed within 24 hours of treatment and they were delivered by Caesarean section at 32 and 33 weeks of gestation. One woman needed intensive care treatment, but both the women and their babies survived without sequelae. Other less dramatic side effects were nausea, vomiting, tiredness, visual impairment, dizziness and loss of sensation.	Excluded

			one had a lethal metabolic disease. There werefive postnatal deaths, including one at two months of age, which was thought to be due to a cardiac issue due to an arrhythmia, as the child had AVRT and was on amiodarone prophylaxis.		
Sridharan, S.	2016	At center 1, 34 outpatient mothers received oralflecainide, usually at an initial dose of 300 mg daily in 3 separate doses. A maternal electrocardiogram was recorded prior to the initiation of treatment. Follow-up was arranged in 1–7 days from treatment onset according to physician judgment. Flecainide dose was decreased to 100 mg twice daily or less following conversion to fetal sinus rhythm (SR). If tachycardia persisted atfirst review, or if the dose was increased subsequently, maternal plasma level (trough) was requested. Addition of amiodarone or digoxin was considered if reversion to SR was not achieved. At center 2, 50 hospital inpatient mothers received intravenous digoxin in a protocol proposed by the Fetal Working Group of the Association of European Paediatric Cardiology.8 The initial digoxin dose was 1.5 mg/24 hours in 3 divided doses, increasing up to 2.0 mg/24 hours in 2 divided doses if required. Digoxin was administered intravenously in short continuous	➢ At center 1, a hydropic fetus with short VA tachycardia, successfully treated withflecainide, required neonatal repair of esophageal atresia and tracheoesophageal fistula and subsequent cardiac surgical repair of tetralogy of Fallot. The infant died aged 7 months, probably because of aspiration, having not receivedflecainide since the neonatal period without observed recurrence of AVRT. This death was not attributed toflecainide exposure. A fetus with variable, but predominantly long, VA time intervals achieved intermittent SR and rate control prenatally. However, multifocal atrial tachycardia persisted for several months postnatally before sustained SR was achieved. There was intrauterine growth retardation, microcephaly, and intracranial calcification. Postnatal IgG and IgM antibodies to cytomegalovirus were detected. Another fetus with long VA tachycardia achieved rate control, but hydrops persisted, necessitating delivery at 38 weeks' gestation. Neonatal ventilatory, inotropic, and antiarrhythmic support was required, but the long-term outcome was good, with sustained SR off treatment. A third fetus with long VA tachycardia achieved rate control within 10 days offlecainide treatment onset, with reversion to SR occurring as late as 80 days later. SR was sustained off treatment postnatally. At center 2, no deaths were observed in nonhydropic fetuses. Intrauterine or neonatal death occurred in 7 of 9 (78%) of those with hydrops not responding to therapy. Interestingly, intrauterine death also occurred in 2 of 12 hydropic fetuses (17%) that had responded to therapy but presented with severely impaired myocardial function.	At center 2, 1 mother whose fetus was found to have a short VA SVT at 34 weeks' gestation did not tolerate digoxin. Sustained tachycardia persisted until 35 weeks' gestation when the fetus was delivered. Digoxin was otherwise well tolerated, despite the relatively high doses used. In another case of short VA SVT and hydrops, the mother did not tolerate treatment. She suffered from psychiatric illness and pregnancy was terminated at 22 weeks of gestation. At center 1, symptoms that might have been related to flecainide occurred in 8 of 34 mothers (24%): in 7, lightheadedness, nausea, headache, or transient blurred vision was reported, and 1 mother described a sensation of heightened alertness. None of these symptoms required cessation of flecainide treatment. Only 1 mother had measured plasma flecainide level greater than 700 μg/L. This was a mother in whom	Mentioned but lack of data.

		infusions until a maternal plasma level of 2.0–3.0 ng/mL was achieved. Maternal plasma levels and electrocardiogram were checked daily at the initial phase of treatment. Once the therapeutic plasma level was obtained, digoxin was administered orally if treatment had been successful, or a second-line drug (usually sotalol) was introduced.		flecainide dose was increased from 100 mg 3 times daily (plasma level 310 μg/L) to 100 mg 6 hourly (plasma level 910 μg/L), which was effective in restoring SR in a hydropic fetus.	
Strizek, B.	2016	β-methyldigoxin was administered as a loading dosage of 800–1000 mg/d (in 4 doses) for 3 days and continued by a maintenance dosage of 500–600 mg/d. If no treatment response was noted after the loading dose phase, serum levels were evaluated and treatment adjusted to achieve levels of 2.0–2.5 ng/mL. Flecainide was administered 100 mg 4 times daily as a loading dosage in hydropic fetuses for 2–3 days and continued with 300 mg/d. In the absence of hydrops, the initial dosage was 300 mg/d. In 1 patient, amiodarone (Cordarex) was added up to 2000 mg/d orally (loading dosage for 4–5 days followed by a maintenance dosage of 400–800 mg/d). Maternalflecainide and amiodarone serum levels were not evaluated.	No follow-up were described	For flecainide group:<br>In 1 asymptomatic mother with tuberous sclerosis, there was a Brugada pattern on the ECG under flecainide therapy, which disappeared after cessation of flecainide. The fetus, which had several cardiac rhabdomyomas, had converted to SR after 6 days and remained in SR even without further treatment. Because of the molecular genetic diagnosis of fetal tuberous sclerosis, the pregnancy was eventually terminated. No other maternal side effects or ECG abnormalities were noted.<br>For digoxin group:<br>One mother had visual symptoms that required reduction of dosage of digoxin,	As additional cardiac findings, 2 fetuses had rhabdomyomas in the context of tuberous sclerosis, 1 fetus had a muscular ventricular septal defect (VSD), and 1 had pulmonary atresia with VSD. 1 fetus showed agenesis of the ductus venosus.

				and 2 patients showed first-degree AV block that was reversible after reduction of digoxin	
Ekiz, A.	2017	Flecainide was initially administered to the mother in an oral dose of 300 mg daily which was given 100 mg three times a day. The maximum dose of flecainide was 400 mg daily. If sinus rhythm was not obtained via flecainide monotherapy within 7 days, the treatment was combined with digoxin. Digoxin therapy was started with a loading dose of 1.5 to 2 mg over 2 days. Digoxin was continued with the dosages between 0.375 and 1mg/day, aiming for between 1.5 and 2.5 ng/mL of digoxin levels.	➢ The newborns were followed up with a mean time of 22.8 months (range 6-48). Although, SR was achieved with antiarrhythmic treatment one fetus was delivered at 32 weeks of gestation, because of having severe signs of hydrops and non-reassuring fetal status. This fetus was the only neonatal death of our series. Five newborns required antiarrhythmic therapy. Only 2 of these were treated prenatally and remaining 3 fetuses did not receive treatment with flecainide because one fetus with AF required prompt delivery due to non-reassuring fetal status and 2 had rhabdomyomas along with intermittent SVT.	One mother developed atrial fibrillation one week after flecainide treatment had started; fortunately, spontaneous resolution of atrial fibrillation was observed after flecainide treatment had been given up. Also of interest: the fetus did not need antiarrhythmic treatment any longer. Additionally, one mother complained of dizziness, but there was no need to cancel or change the therapy. We have not experienced any other adverse reaction to flecainide treatment, including visual disturbances, nausea and palpitation.	2 fetuses had cardiac rhabdomyomas ; hence, they had the diagnosis of tuberous sclerosis after birth.
Karmegeraj, B.	2018		➢ In the SVT group, one infant had a recurrence while on propranolol triggered by salbutamol nebulization. This responded to adenosine and the beta-blocker was to bisoprolol. The median duration of follow-up was 10.5 (3–64) months in the SVT group and the median duration of maintenance therapy was 5 weeks (3–16). In the AF group, were no complications or recurrence on follow-up. The median duration of follow-up was 4 (1–19) months and duration of maintenance antiarrhythmic therapy was 3 (1–19) weeks. Details of comparison between SVT and AF groups were presented in original article in form of table.		

Miyoshi, T.	2019	Rapid initial saturation was performed with a 0.5mg intravenous injection. Intravenous injections of 0.25 mg were administered at 8 and 16 h after the initial dose. If intravenous injections were not feasible, oral digoxin (1.5 mg/day) was given in 3 divided doses. Subsequently, oral digoxin at 0.75 mg/day was given in 3 divided doses, with adjustment to maintain maternal serum concentrations from 1.5 to 2.0 ng/ml.<br>Sotalol at 160 mg/day in 3 divided doses was added. If a regimen of 3 days of treatment at 160 mg/day of sotalol was ineffective, the patient then proceeded to 240 mg/day in 2 divided doses. If 240 mg/day was ineffective after 3 days of treatment, the patient proceeded to 320 mg/day in 2 divided doses.	Fetal AEs related to transplacental treatment were observed in 12 fetuses (24.0%). Serious AEs resulting in discontinuation of the protocol-treatment occurred in 4 fetuses. Fetal death occurred in 2 of 49 fetuses overall (4.1%; 95% CI: 0.5% to 14.0%). In 1 fetus heavy for gestational age (>5.0 SD), AFL developed with ascites, cardiac effusion, and polyhydramnios at 26 weeks of gestation. After digoxin and sotalol combination therapy, the frequency and ventricular rate of fetal AFL decreased, but hydrops progressed, resulting in fetal death at 27 weeks of gestation. Postmortem examination showed hypoplastic lungs, small ears, and flexion of the long finger in both hands, findings suggesting Costello syndrome. Another fetus of 34 weeks of gestation with a diagnosis of short VA SVT once achieved sinus rhythm and resolved pleural effusion and ascites after digoxin and sotalol, but the tachyarrhythmia recurred at 36 weeks of gestation. SVT was sustained even after increased dosage of sotalol to 240 mg/day, and the fetus developed pleural effusion and ascites. The fetus died at 37 weeks of gestation during preparations for cesarean section. These fetal deaths were mainly caused by progression of fetal heart failure secondary to AFL and SVT. In 1 fetus with AFL, 7:1 AV block was observed after starting the combination of digoxin and sotalol. Because the ventricular rate decreased to 50 beats/min for 5 min at 36 weeks of gestation, cesarean section was performed, and the newborn was treated by electrical cardioversion. In another fetus with AFL, 1:1 AV conduction at 275 beats/min was observed 5 days after starting the combination of digoxin and flecainide. The fetus progressed to ascites and pleural effusion and was delivered by cesarean section at 32 weeks of gestation. AFL resolved spontaneously just after birth.	Although maternal AEs related to transplacental treatment were observed in 39 patients (78.0%), there was only 1 serious event: Mobitz type II AV block was observed but resolved immediately after temporary discontinuation of digoxin and sotalol. Nausea or vomiting, the most common maternal adverse symptom, was observed in 27 patients (54.0%). Electrocardiographic abnormalities were detected in 19 patients (38.0%). Elevated brain natriuretic peptide concentrations were found in 25 patients (50.0%). Despite a relatively high incidence of maternal AEs, dose reduction allowed for continuation of transplacental treatment.	Excluded
O'Leary, E. T.	2020	Digoxin is administered as a 1-g oral load over 24 hours in three divided doses (eg, 0.5, 0.25, and 0.25 mg). Maintenance dosing is titrated to target a maternal serum level of 1 to 2 ng/mL. Initial flecainide dosing is 100 mg by mouth, three times per day, with	Neonatal records were available for review in 61 of 65 (94%) cases of fetal SVT, with 31 of these infants having a documented episode of clinical SVT at a median age of 0 days (0-62). Manifest pre-excitation was present in eight neonates. Patients had between zero and five episodes of documented SVT with 30 (49%) having no episodes and six (10%) having two or more episodes. The latest presentation of accessory pathway-mediated SVT (ie, AVRT or PJRT) was 39 days of life. All of the seven infants with postnatal AFL presented on the first day of life, underwent successful esophageal pace termination or direct current cardioversion, and had no additional episodes beyond this. One infant with a history of fetal EAT had a first	Unmentioned	Excluded

		further dose adjustments dictated by fetal response and maternal serum flecainide levels (>250 mcg/L but <1000 mcg/L). Initial sotalol dosing is 80 mg by mouth, three times per day; sotalol levels are not routinely checked.	postnatal episode of EAT at 62 days of life. Two neonatal deaths occurred during the study period. One female neonate was diagnosed with fetal AVRT at 21 weeks gestation and remained in incessant SVT for 2 weeks on digoxin monotherapy before transfer to Brigham and Women's Hospital. Flecainide and sotalol monotherapy, as well as intraumbilical adenosine administration, were all tried without successful conversion. Amiodarone and flecainide were started in combination at 26 weeks gestation which ultimately resulted in adequate ventricular rate control (~170 bpm).		
Tunca Sahin, G.	2021	Loading doses of **digoxin** were given on the first day of treatment with an initial dose of 500 µg followed by 250 µg, and 250 ug doses every 8 h. **Flecainide** was initiated concomitantly at the dose of 100 mg three times a day.	➢ Of these sixteen cases, ten babies (62.5%) were born in sinus rhythm. Six babies (37.5%) were in AF at birth (with variable atrioventricular block) and were treated with DC cardioversion to restore sinus rhythm. Two patients remained in sinus rhythm, but four patients had AVRT immediately after DC cardioversion development, and AVRT was observed in one patient on a Holter recording a week after delivery. These five patients (31.3%) required postnatal antiarrhythmic treatment for up to 2 years. All patients remained arrhythmia-free after discontinuation of treatment. There was no recurrence of atrial flutter in the long term. The ECGs of four patients with AVRT exhibited pre-excitation, which disappeared in two patients during follow-up . One patient continued to show asymptomatic pre-excitation on ECG but did not develop arrhythmia following termination of atrial flutter with DC cardioversion. RFA was successfully performed at the age of 6 years. Intriguingly, during the RFA procedure this patient developed multiple episodes of preexcited atrial fibrillation with rapid ventricular response requiring repeat DC cardioversion. One asymptomatic patient was referred for RFA after stopping medication owing to persistent pre-excitation at 8 years of age (Fig. 2). The median duration of follow-up was 4.5 years (range 0.6–16). No neurological morbidity was documented in the surviving neonates.	Unmentioned	Excluded
