# Supplementary material for: Efficacy and Safety of Various First-Line Therapeutic Strategies for Fetal Tachycardias: A Network Meta-Analysis and Systematic Review
Source: Front Pharmacol. 2022 Jun 13;13:935455. doi: 10.3389/fphar.2022.935455 (PMC9235149; doi:10.3389/fphar.2022.935455)
Supplement: Supplementary file 2 [file DataSheet4.PDF]

Data extraction of included studies in Total group:

Author	Year	Treatment for total	Total for total	Cardioversion for total	Death for total
van Engelen, A. D.	1994	D	24	11	1
		F	10	7	0
Frohn-Mulder, I. M.	1995	D	28	13	NA
		F	7	3	NA
		DF	4	4	NA
Naumburg, E.	1997	D	10	4	0
		DF	1	0	0
		DV	4	0	0
Lisowski, L. A.	2000	D	21	16	1
		DS	5	4	0
		S	9	8	1
Oudijk, M. A.	2000	DS	7	5	1
		S	12	9	3
Ebenroth, E. S.	2001	D	37	17	1
		DF	13	12	0
		FS	1	1	0
Jouannic, J. M.	2002	A	5	2	0
		D	32	26	1
		DA	1	1	0
		DS	1	1	0
		S	1	1	0
Krapp, M.	2002	D	13	4	0
		DF	7	7	0
Boldt, T.	2003	D	26	14	NA
		DF	3	2	NA
		DS	3	2	NA
Jouannic, J. M.	2003	A	4	2	1
		D	7	0	0
		DS	2	0	0
		F	12	7	1

Oudijk, Martijn A.	2003	DS	2	0	0
		S	7	7	1
D'Alto, M.	2008	D	6	5	0
		DF	6	5	1
		DS	2	2	0
Pezard, P. G.	2008	D	16	7	NA
		F	2	1	NA
Lulic Jurjevic, R.	2009	D	2	2	0
		DF	1	1	0
		DS	2	2	0
		F	6	3	3
Hahurij, N. D.	2010	D	8	5	0
		DF	2	1	0
		F	4	3	0
		S	5	4	0
Shah, A.	2012	DS	12	9	3
		S	9	8	0
van der Heijden, L. B.	2012	DS	3	1	0
		F	2	2	0
		S	22	22	0
		FS	3	3	0
Uzun, O.	2012	D	6	0	0
		DF	21	16	1
		F	1	0	0
Ekman-Joelsson, B. M.	2015	D	46	19	1
		DS	32	14	5
		F	2	2	0
		S	32	19	0
Sridharan, S.	2016	D	29	23	NA
		F	27	26	NA
Strizek, B.	2016	D	14	2	0
		DF	6	6	0

		F	28	21	1
Ekiz, A.	2017	DF	1	1	0
		DFS	1	0	1
		F	16	15	1
Karmegeraj, B.	2018	D	3	3	0
		DF	6	5	1
Miyoshi, T.	2019	D	42	25	0
		DS	3	1	0
		S	4	4	0
O'Leary, E. T.	2020	D	35	25	NA
		F	3	2	NA
		S	1	0	NA
Tunca Sahin, G.	2021	DF	12	9	0
		F	1	0	0

D, Digoxin; DF, Digoxin and Flecainide; DS, Digoxin and Sotalol; F, Flecainide; S, Sotalol; DV, Digoxin and Verapamil; DA, Digoxin and Amiodarone; A, Amiodarone; DFS, Digoxin, Flecainide and Sotalol; SF, Sotalol and Flecainide

Safety data extraction of included studies in Total group:

Author	Year	Dosage	Neonatal and follow-up adverse events	Maternal side effects	Structural heart disease
van Engelen , A. D.	1994	Initially treated by maternally administered digoxin (intravenous loading dosage of 1.5 mg/day, given in three parts, followed by an oral maintenance of 0.5 to 0.75 mg/day) (n=14) or flecainide (twice a day 100 to 150 mg orally) (n=5).	In the group of patients with supraventricular tachycardia, a reentry mechanism could be seen on the electrocardiogram in 8 (4 Wolff-Parkinson-White syndrome, of whom 2 had no more tachycardia after birth; 4 permanent junctional reciprocating tachycardias). At age of 1 month, 78% of the patients with a history of fetal supraventricular tachycardia or atrial flutter were receiving antiarrhythmic drugs (digoxin, sometimes in combination with propranolol, verapamil or flecainide), either for recurrent tachycardia or as antiarrhythmic prophylaxis, and at 3 years, 14% were taking antiarrhythmic drugs. The 2 patients with permanent junctional reciprocating tachycardia in this group of 22 were still receiving medication at the age of 3 years.	In the four cases in which procainamide was used, three mothers complained of nausea and vomiting. Two of the 15 mothers receiving flecainide had blurred vision and dizziness that resolved after the dos decreased.	Unmentioned

Frohn-Mulder, I. M.	1995	To restore fetal sinus rhythm, digoxin was the first drug of choice. After maternal administration of a loading dose of 1 mg, a maintenance dose of 0 2 5 mg, three times a day, was given. Serum drug concentrations were measured and the dosage adjusted to maintain concentrations around 2 nanograms/milliliter (ng/ml). When sinus rhythm could not be achieved other drugs were administered to the pregnant woman such as verapamil; or occasionally propranolol, procainamide or amiodarone.As from January 1991, flecainide was selected as the first drug of choice in cases of fetal hydrops, and was also administered if treatment with digoxin failed to restore sinus rhythm in the non-hydropic fetus. Flecainide was administeredorally 3 times 100 mg a day; the dosage was adjusted if necessary to keep serum drug concentrations between 0.5-1 microgram/milliliter (mcg/ml).	In 22 fetuses there was no hydrops, Postnatal treatment was necessary in 19 infants. In 17 infants digoxin alone was sufficient to control normal rhythm. In two infants with SVT flecainide was given after digoxin failure. These two infants had also not reacted to digoxin prenatally. Postnatal treatment was discontinued after one week in one case because of bradycardia. In all other cases, therapy was discontinued after one year, without recurrence of SVT. Of the seven newborn which presented with hydrops, all but one needed treatment. Two infants remained in sinus rate by digoxin alone, in one case propranolol was added. In two infants, flecainide was continued. In these infants therapy could be withdrawn after one year without recurrence of SVT. In another infant, which applied to have an ectopic atrial tachycardia and was difficult to manage prenatally, control of heart rate was finally achieved by sotacor. When this medication was withdrawn after two years there was a recurrence of SVT within six months, necessitating further sotacor medication. One infant died on the second day after delivery because of complications of ongoing tachycardia and severe hydrops at 31 weeks of gestation.	Unmentioned	An atrial septal aneurysm was established in 3 fetuses. No other structural abnormalities were found and no viral infections were documented.

Naumbu rg, E.	1997	0.5-1.25 mg/day, oral or intravenously, b.i.d. for Digoxin	During the neonatal period, 13 infants were started on digoxin therapy, and in 5 this was combined with drugs such as furosemide, isoprenaline or dopamine to treat the heart failure. The mean duration of digoxin treatment was 7.5 months (range 6-12 months). One infant with WPW syndrome was treated with verapamil as monotherapy due to insufficient effect of digoxin. Another infant needed electric conversion as well as digoxin at birth to convert the tachycardia. One infant with an intrauterine tachycardia diagnosed as FSVT, which resolved spontaneously, developed tachycardia with an atrial flutter at 6 months of age. She needed an electric cardioversion and was then treated with digoxin for 6 months. Another child with antenatal atrial flutter/fibrillation, who received digoxin for 7 months after birth, developed paroxysmal tachycardia at 2 years of age. The tachycardia resolved by a vagal maneuver. A 3rd child with antenatal FSVT treated with digoxin during his 1st year of life due to a WPW syndrome developed tachycardia at 7 years of age. The tachycardia resolved spontaneously.	Nothing serious apart from nausea	Excluded
Lisowsk i, L. A.	2000	Detailed protocols were described from case to case.	Of the original 45 patients, 2 patients died in utero, and 12 of the 43 live-born infants were in AF at birth. Twelve-lead ECG confirmed AF with monomorphic undulating negativeflutter waves in leads II, III, and avF (common type). Flutter waves were commonly seen in lead V1. No sign of aberrant conduction was noted on these ECGs. Three infants were in serious trouble with poor Apgar scores at birth. In four of the 45 patients, including the one described above, neurological morbidity was documented immediately postnatally, suggesting an association with the prenatally existing arrhythmia. The neurological damage ranged from severe cerebral hypoxicischemic lesions to intraventricular hemorrhage, resulting in a hydrocephalus. One patient had a small periventricular infarction that is resolving.	Unmentioned	Excluded
Oudijk, M. A.	2000	The starting dosage used was 80 to 160 mg of sotalol, given orally 2 times a day. The dosage was occasionally increased to a maximum of 160 mg 3	Follow-up was possible in 17 cases (4 intrauterine deaths occurred). No rhythm disturbances were seen in 11 of the 17 surviving patients with fetal tachyarrhythmias (65%). Prophylactic	In 2 cases, maternal adverse effects were encountered. They were only temporary.	Excluded

times per day if tachycardia persisted. Digoxin was added to the treatment in patients in whom adequate control could not be achieved with sotalol as a single therapy.

drug therapy was administered for 9 months to 1 year in 5 of these 11 patients; 2 patients received sotalol, and the other 3 patients received digoxin. None of these patients have shown recurrent signs of tachycardia, and they are currently doing well. A relapse of tachycardia was seen in 6 of the 17 cases (35%). Two patients had AF and 3 had SVT. The child with VT had recurrent VT after birth. Two patients were successfully treated with sotalol, 1 patient was treated with digoxin, and 1 patient received a combination of sotalol and digoxin. The fifth patient had AF and was electrically cardioverted to restore sinus rhythm,; sotalol was also administered. All newborns were treated until the age of 1 year. Two patients with fetal hydrops had significant neurological morbidity immediately after birth. One had SVT and was treated with sotalol, the second had AF and was treated with sotalol and digoxin. Before conversion to persistent sinus rhythm was achieved, these patients experienced intermittent episodes of tachycardia with long-lasting periods of normal sinus rhythm. These episodes lasted 10 and 21 days, respectively, at the gestational ages of 29 and 25 weeks, respectively. Although control of the tachycardia was achieved and these babies were born with good Apgar scores, their post- natal evaluation showed neurologic pathology; this was due to intracranial hemorrhage in one and cerebral hypoxic ischemia in the other.

Ebenroth, E. S.	2001	Digoxin at 1-1.5 mg, either i.v. or PO based on physician preference, over 24 hours and they were started on a maintenance dose of 0.25 to 1 mg/day. Doses were adjusted to attain high therapeutic levels of 1.4-2.0 ng/ml whenever possible. All patients since 1990 have received flecainide. Flecainide was administered to the mother in an oral dose of 100-500 mg/day divided b.i.d. to t.i.d. and titrated to obtain therapeutic maternal drug levels. Flecainide levels were obtained when doses greater than 300 mg/day were necessary.	The only patient with twins in our study developed HELLP syndrome (a severe form of preeclampsia characterized by Hemolysis, Elevated Liver function tests, and Low Platelets) at 34 weeks gestation, and the infants were delivered. Infant A was born with a stable wide complex tachycardia, whereas infant B, the one being treated for fetal tachycardia, had markedly prolonged QRS duration. Flecainide levels sent from cord blood revealed high therapeutic values of 1.0 and 0.78, respectively. Both infants did well, and after 2 days off flecainide, their electrocardiograms had normalized. Twin B then developed recurrent SVT. There were three postnatal deaths in this population of patients. The first was a patient with diaphragmatic hernia and hydrops at presentation. The child was converted with digoxin alone and born at term in sinus rhythm. The second was the other patient with diaphragmatic hernia, who was also converted on digoxin alone, and who remained in sinus rhythm until birth at term. Both of these infants died from complications of their diaphragmatic hernias within the first 2 hours of life. The third was the patient mentioned previously, who presented at 19 weeks gestation in SVT with hydrops and oligohydramnios. He was converted with digoxin and flecainide and was delivered at 29 weeks gestation in sinus rhythm. He subsequently died at 18 days of age following an episode of necrotizing enterocolitis, sepsis, and multisystem organ failure. In all three cases, hydrops had resolved, and sinus rhythm had been restored prenatally. They were continuously monitored in the Neonatal Intensive Care Unit, and all maintained sinus rhythm until their deaths.	In the digoxin group, one woman suffered from transient Mobitz type II second-degree atrioventricular block on a therapeutic dose of digoxin. Her digoxin dose was weaned, she was started on flecainide with conversion of the fetus, and the heart block resolved. Women in the flecainide group demonstrated mild to moderate prolongation of QRS durations, but none developed any significant dysrrhythmia nor experienced any adverse effects. There was only one delivery prior to 34 weeks gestational age. This fetus presented at 19 weeks gestational age in SVT with hydrops and oligohydramnios. Following conversion with digoxin and flecainide, she was discharged. She returned one week later with vaginal bleeding and had persistent vaginal bleeding for the next 8 weeks until preterm labor and delivery occurred. It is not clear that this was related to the anti-arrhythmic therapy, but this possibility cannot be completely ruled out.	38/40 fetuses possessed structurally normal hearts. 1 suffered from tuberous sclerosis with multiple rhabdomyomas; the other had a dilated cardiomyopathy that was postnatally diagnosed as mild Ebstein's anomaly. Two of the fetuses were known to have left-sided diaphragmatic hernias prior to the diagnosis of tachycardia.
Jouannic, J. M.	2002	Digoxin was started at a dose of 0.25 mg three times daily and the dose was adjusted to achieve a maternal serum concentration within the therapeutic range (1.5–2 ng/ml). Maternal amiodarone treatment was given at a loading dose of 1,600–2,000 mg/day during 2 days and then reduced to 400–600 mg/day. Flecainide was given	In group A (no tricuspid regurgitation, n = 30), One of the 3 fetuses in whom digoxin failed died in utero at 28 weeks, 8 days after starting treatment. In another case, digoxin was replaced by flecainide after 10 days, because ascites and pericardial effusion appeared. Although conversion to sinus rhythm was not achieved, the fetal heart rate decreased under flecainide, and hydrops	Unmentioned	The cardiac structure was normal in all but 1 case in which a ventricular

at a dose of 100 mg three times daily. Sotalol was given at a dose ranging from 160 to 240 mg/day.

improved before birth. In the last case, digoxin was continued despite persistence of the arrhythmia. A Caesarean section was performed at 36 weeks for prolonged rupture of membranes. This child was converted to sinus rhythm by postnatal amiodarone therapy. The first-line treatment of the remaining 5 children in group A was digoxin and amiodarone in 1, digoxin and sotalol in 1, sotalol in 1 and amiodarone in 2. All of them were converted prenatally. In group B (tricuspid regurgitation, n = 10), prenatal restoration of sinus rhythm was achieved prenatally in 5 cases (50%) within a mean time interval of 24 days. Seven fetuses were initially treated with digoxin alone, and 3 fetuses received amiodarone. After using digoxin as firstline treatment, 5/7 cases converted (71%). After using amiodarone, none of the 3 cases converted. Two cases were complicated by hydrops 7–10 days after starting fetal therapy. In 1 case, hydrops resolved 15 days after digoxin had been added to amiodarone but sinus rhythm could not be achieved before birth. In the other case, hydrops increased and the baby was delivered by Caesarean section at 36 weeks. No further follow-up were described.

septal defect was diagnosed prenatally and repaired at 8 months of age

Krapp, M.	2002	All patients were treated as inpatients before cardioversion of the fetus. When fetal sinus rhythm was obtained, women were monitored as outpatients twice weekly. Drug therapy was continued until delivery.β-methyldigoxin, which is rapidly metabolized to digoxin in the maternal liver, was used as first line treatment in all cases (Table 1). After 3 days, the intravenous loading dose of 800–1000 µg per day was followed by an oral maintenance dose of 500– 600 µg per day. Maternal cardiac conduction was monitored by assessing for first degree heart block on daily electrocardiograms (ECG). Serum digoxin levels were maintained between 1.5 and 2.5 ng /mL. Maternal serum potassium, sodium, magnesium, creatinine and liver enzymes were monitored weekly. When supraventricular tachycardia persisted longer than 48–72 h after the initiation of drug therapy, in the first 16 months of this study, verapamil was added at an oral dose of 80 mg four to six times daily. Flecainide was always combined withβ-methyldigoxin and used as first line treatment, as second line treatment, or as third line treatment replacing verapamil when fetal supraventricular tachycardia persisted over 72 h. The dose was 100 mg three to four times daily orally, while serum flecainide levels were maintained between 500 and 1000 µg / L. Daily maternal ECGs were monitored by assessing for prolongation of QRS duration. In all cases, antiarrhythmic therapy was continued until delivery.	Two newborns with sinus rhythm were prophylactically kept on digoxin, which was arbitrarily terminated after 4 and 8 weeks, respectively. Six newborns had recurrence of SVT and were treated with antiarrhythmic drugs. One newborn was treated with digoxin alone. In two cases, propafenone alone and, in one case, propafenone in combination with digoxin was used. Another two cases were treated with propranolol alone and in combination with digoxin, respectively. Nineteen of 20 infants showed normal development after 2 years. However, one infant (Case 11), who was delivered at 33 + 1 weeks of gestation after conversion to sinus rhythm in hydropic condition because of preterm premature rupture of membranes, had multiple disseminated skin lesions (blueberry muffin eruptions) at birth suspicious for extramedullary erythropoiesis. The Apgar scores were 5, 7, and 8 after 1, 5 and 10 min, respectively. Arterial blood pH (7.31) and hemoglobin (114 g / L) were within normal limits. At the age of 2 years, there was mild periventricular leukomalacia verified by magnetic resonance imaging. A short episode of tachycardia was controlled by propafenone during the neonatal period. The infant showed normal motoricity, but bilateral dysacousia and disturbed development of speech.	There were no maternal side-effects during flecainide treatment.	Excluded
Boldt, T.	2003				

	The drugs used for oral treatment of the mother included digoxin, propranolol, sotalol,flecainide, or amiodarone. Digoxin treatment was provided orally at a dosage of 0.25 mg two or three times daily, with the dosage adjusted to achieve a maternal serum concentration in the therapeutic range (1.5 to 2.0 ng/mL). Propranolol was provided orally at a dosage of 40 mg three times a day and sotalol from 80 to 120 mg twice a day. Flecainide was provided orally at a dosage of 100 mg two or three times a day and amiodarone 150 to 300 mg daily. Direct fetal amiodarone therapy (triple-route administration by use of combined direct fetal intravenous, direct fetal peritoneal, and maternal oral amiodarone) consisted of amiodarone on the basis of estimated fetal weight (7.5 mg/kg).	Mentioned but lack of data for drug therapy group was just a part of its analysis.	Mentioned but lack of data for drug therapy group was just a part of its analysis.	Mentioned but lack of specific data for drug therapy group was just a part of its analysis.

Jouannic, J. M.	2003	Anti-arrhythmic drugs used for oral treatment included digoxin, amiodarone,flecainide and sotalol. Digoxin was started at a dose of 0.25 mg three times daily and the dose was adjusted to achieve a maternal serum concentration within the therapeutic range (0.8 to 2 ng/mL). Maternal amiodarone treatment was given at a loading dose of 1600 to 2000 mg per day for two days and then reduced to 400 to 600 mg per day until conversion to sinus rhythm, as previously reported (Cuneo et al., 2000; Arnoux et al., 1987). Flecainide was given at a dose of 100 mg three times daily. Sotalol was given at a dose range from 160 to 240 mg per day. Following the report byAllan et al. (1991), our policy changed andflecainide was used asfirst-line therapy. Before that report, either digoxin or digoxin + sotalol were used asfirst-line therapy. Amiodarone was used in cases in which fetal cardiac examination revealed poor ventricular function. Whenfirst-line therapy failed to restore sinus rhythm, amiodarone alone or in association with digoxin was used as second-line therapy.	In one case, amiodarone therapy was started at 24 weeks in a fetus with major hydrops and poor ventricular systolic function with tricuspid regurgitation. A TOP was decided at 26 weeks because of increasing hydrops and no spontaneous fetal movement. In another case, amiodarone was started at 37 weeks leading to a decrease in the fetal heart rate that remained below 220 bpm, but with no conversion to sinus rhythm. This fetus was delivered vaginally 2 weeks later and was converted to sinus rhythm with intravenous digoxin therapy.<br>Two perinatal deaths occurred within 24 h of starting flecainide treatment. In the first case, flecainide was started at 34 weeks in a fetus with minor ascites, normal ventricular contraction and no atrioventricular regurgitation. Ten hours after the first dose, a systematic sonographic examination revealed severe permanent bradycardia (30 bpm). The fetus was delivered by emergency cesarean section but died despite resuscitation. In the second case, the fetus died in utero 8 h after commencing flecainide. The fetus had mild ascites, decreased ventricular contraction and mild tricuspid regurgitation. In one fetus, the fetal heart rate decreased on flecainide but remained above 210 bpm, and there was no conversion to sinus rhythm. The hemodynamic condition deteriorated with major tricuspid regurgitation and poor ventricular contraction. A termination of pregnancy (TOP) was performed at 27 weeks because hydrops worsened with no fetal movements. The parents declined autopsy.<br>All live newborns with persistent SVT were converted in thefirst week of life using either digoxin or amiodarone intravenous therapy. One infant who developed postnatal hypotonia associated with growth retardation and inherited mitochondrial cytopathy was diagnosed at the age of four months. Of the 11 live neonates who were treated with amiodarone in the prenatal period, the thyroid stimulating hormone (TSH) level was elevated at day 3– 4 in two cases (60.3 and 51.8 mU/L, normal<20 mU/L). Those two neonates	Maternal tolerance of anti-arrhythmic drugs was good in all cases but one. Digoxin intoxication occurred in one case revealed by abdominal pain and vomiting on day 5. The patient had received amiodarone forfive days at a loading dose of 2000 mg per day in association with digoxin 0.5 mg twice daily. Maternal serum digoxin concentration was 3.4 ng/mL. The maternal electrocardiogram showed an increased PR interval. Digoxin was stopped and amiodarone was reduced to 1200 mg per day. Signs of digoxin intoxication were resolved in 2 days.	Excluded

received thyroid hormone substitution therapy for a period of two and three months, respectively. The postnatal course, including neurological development, was normal for all infants in the prenatal amiodarone therapy group with a follow-up ranging from seven months to four years.

Oudijk, Martijn A.	2003	Sotalol therapy was initiated at either 80 mg twice daily or 160 mg twice daily, increased to a maximum of 160 mg thrice daily, and the addition of digoxin in the event of conversion to sinus rhythm did not occur (18,22). Patients were regularly scheduled (at least once a week) for control visits to evaluate the fetal heart rhythm and possible signs of congestive heart failure.	Follow-up is available for the other 16 infants, varying from 6 months to 36 months after birth. Eight infants (5 had AF and 3 had SVT) had no rhythm disturbances during the newborn period, and no medication was initiated. None of these patients have developed episodes of tachycardia, and they are currently doing well. Three patients showed AF at birth (two therapy-resistant cases and one relapse), and all required electrical cardioversion to reach sustained sinus rhythm. These three patients are currently doing well and require no medication. Five patients showed SVT postnatally, of whom two had Wolff-Parkinson-White syndrome, two had persistent junctional reciprocating tachycardia, and one patient showed intermittent periods of SVT of unknown origin. All are doing well on antiarrhythmic therapy, consisting of digoxin in two patients, propranolol in one patient, and a combination of these two agents in two patients. All surviving infants are in good neurologic condition.	Unmentioned	Unmentioned
D'Alto, M.	2008	The starting digoxin dosage was 1– 2 mg i.v. over 36 h (1/2 dose þ 1/4 dose after 12 h and 1/4 dose after 24 h); maintenance dose: 0.250 mg, 2– 4 times a day orally (mean of 0.012 ± 0.004 mg/kg/day), monitoring serum digoxin level. The starting dosage of sotalol was 80–160 mg, given orally twice a day. This dosage was occasionally increased o a maximum of 160 mg, 3 times a day if tachycardia persisted (mean of 5 ± 2 mg/kg/day). The starting dosage offlecainide was 50–100 mg given orally twice a day, occasionally increased to a maximum of 200 mg twice a day (mean of 3 ± 2 mg/kg/day).	Follow-up was possible in 33 of 36 cases (one intrauterine and two neonatal deaths occurred). Four fetuses with tachycardia had congenital heart disease: one Ebstein anomaly, one aortic coarctation (both prenatally diagnosed), and two atrial septal defects. Twenty out of the 33 surviving patients, (19 with 1 : 1 AV tachycardia and one with prevalent 2 : 1 AV tachycardia), underwent transesophageal electrophysiological (EP) study in washout therapy soon after birth (mean age of 3.5 ± 2.2 days). In one patient with incessant AVRT during fetal life, no tachycardia was induced at postnatal EP study. Prophylactic drug therapy was postnatally administered in 16 patients with spontaneous or EP study-induced tachycardia (Table 1). None of these patients have shown recurrent signs of tachycardia, and they are currently doing well. The child with LQTS had recurrent VT after birth until he was 3 months old. At 2.8-year follow-up, he is taking propranolol and mexiletine and is asymptomatic and event free. Thirteen patients repeated the EP study in washout at 1 year of life: in four	A close maternal evaluation with physical examination, ECG, echocardiogram and serum digoxin level (when this drug was used) was performed before starting therapy and during the gestation. No maternal adverse effects were encountered.	2 out of 36 fetuses prenatally showed a congenital heart disease: one Ebstein anomaly and one aortic coarctation (both confirmed at birth). Seven fetuses were hydropic at the prenatal

			of 13, no tachycardia was induced, and medical therapy was discontinued. At a 3 ± 1.1-year follow-up, 33 out of 35 live-born children of our study were alive and well and none of them suffered from late neurological disease.		echocardiographic evaluation.
Pezard, P. G.	2008	digoxin, loading dose 1—1.5 mg/day twice daily, maintenance dose 0.5 mg/day. 300 mg/day	Of the 21 neonates for whom we have follow-up information (ranging from two months to 17 years), nine infants had recurrence of tachycardia: eight SVT required postnatal maintenance therapy (38% of follow-up), including all four cases that had evidence of pre-excitation on neonatal electrocardiogram. The fetus with VT had some runs of VT after birth, with spontaneous recovery at one month of life.	In our case series, maternal tolerance to amiodarone was good, apart from transient prolongation of the PR interval in three mothers. No maternal thyroid complications were observed. Two patients given the amiodarone—digoxin combination showed elevated levels of serum digoxin (up to 5 ng/ml) and clinical signs of digitalis intolerance, leading to the discontinuation of the digoxin treatment and the administration of amiodarone alone. Amiodarone is known to raise the plasma levels of digoxin, which therefore necessitates careful dosage adjustments	Unmentioned
Lulic Jurjevic, R.	2009	The protocol for treatment was described in original article in form of flow chart	Adenosine was used as a first line drug in 11 newborns, 6 with supraventricular tachycardia, 4 with atrial ectopic tachycardia, and 1 with permanent junctional reciprocating tachycardia. The drug proved successful in terminating the tachycardia in all patients with supraventricular tachycardia, and in a single patient with permanent junctional reciprocating tachycardia, but failed to sustain a regular sinus rhythm in all of them for more than a few seconds. Adenosine failed to convert any of 4 patients with atrial ectopic tachycardia. Following adenosine, amiodarone was introduced in 9 patients and propafenone in 2 patients. In an additional 2 patients, one with atrial flutter and one with ventricular tachycardia respectively, amiodarone was introduced as a first line drug. Of 13 patients receiving amiodarone or propafenone, 8 converted on amiodarone or propafenone alone, and the remaining 5 after propranolol was	Unmentioned	A concomitant congenital cardiac defect was identified in 4 fetuses (13.8%), including small muscular ventricular septal defects in 2, a nonrestrictive perimembranous ventricular

added to amiodarone. In 1 patient with atrial flutter, conversion occurred during insertion of a catheter into the umbilical vein. The median time to conversion was much longer in patients with atrial ectopic tachycardia and permanent junctional reciprocating tachycardia than in those with supraventricular tachycardia, atrial flutter, or ventricular tachycardia. Long-term antiarrhythmic prophylaxis was administered in 17 patients, 14 of those born in tachycardia, 2 patients in whom tachycardia recurred after birth, and 1 patient with WolffParkinson-White syndrome. Amiodarone was prescribed for 7 patients in isolation, together with propranolol in 6, while 2 received propafenone and 2 methyl– digoxin. The median period of treatment was 12 months, with a range from 0.13 to 52 months, and the median period of follow-up was 3.5 years, with a range from 0.05 to 7 years. The baby born with ventricular tachycardia having trisomy 18 died at the age of 3 weeks. 25 of the cohort have survived over the long-term (86.2%). Excluding the patient with trisomy 18, only patients with fetal hydrops suffered mortality, with 37.5% of this group dying. The difference from the group of non-hydropic fetuses was significant, the value for p equal to 0.03. Among the 5 long-term survivors of those presenting with fetal hydrops, 1 patient has a severe neurological and cognitive handicap. There was no evidence of neurological impairment in any of the long-term survivors from the group presenting with preserved cardiac function.

septal defect in 1, and Ebstein's malformation in the other.

Hahurij, N. D.	2010	During the study period, the following drugs were used: digoxin, sotalol,flecainide, amiodarone and adenosine. Digoxin was administered to the mother in adjusted oral doses to maintain a maternal serum therapeutic level of 1–2 ng/mL (loading dose 2 × 0.75 mg, maintenance 0.25–0.5 mg, maximum 0.75 mg/daily). Flecainide (oral dose 200–400 mg daily) and sotalol (oral dose 2 × 80–160 mg daily) were used as secondary agents. Amiodarone was administered by combined direct fetal intravenous and maternal oral and intravenous route. Direct fetal amiodarone therapy consisted of amiodarone on the basis of estimated fetal weight (10 mg/kg).	The overall incidence of cardiac anomalies in the study population was 18% (8/44). In the SVT group 1 infant had a ventricular septal defect and 1 infant had cardiomyopathy, poly valvular disease and pulmonary stenosis. In the AF group, 1 infant was found to have coarctation of the aorta. In the AVB group, 5 of 9 infants had complex CHD (congenitally corrected transposition of the great arteries (cc-TGA), n = 2; left atrial isomerism, n = 1; ventricular septal defect, pulmonary stenosis, cardiomyopathy, n = 1; endocardial fibroelastosis, n = 1). Postnatally, AVB block remained present in all survivors (n = 6) and 5 patients received pacemaker therapy immediately after birth. In 67% of AF-fetuses and 78% of SVT-fetuses episodes of tachycardia or incessant tachycardia remained present after birth. Nineteen of the 28 children in the SVT group were treated with medication after birth. SVT was self-limiting in 74% (14/19), and treatment could be stopped within thefirst year of life. Five of 28 fetal SVT (AVRT) cases had WPW-syndrome, as demonstrated by the presence of ventricular preexcitation on the ECG at birth. In 2 cases, radiofrequency catheter ablation of an accessory pathway was performed in the first months of life due to drug-refractory tachycardias. AF was treated with anti-arrhythmic therapy (n = 4) or cardioversion (n = 2). After initial conversion to sinus rhythm, AF did not recur in all 6 cases. Interestingly, in two AF cases the presence of an accessory pathway was demonstrated. One AF case showed WPW-syndrome on ECG after cardioversion, another AF case developed recurrent AVRT requiring anti-arrhythmic therapy. Twenty-three children were examined in the SVT group. No cases still required drug therapy, and no cases were treated with catheter ablation after thefirst year of life. Twenty-two of 23 patients were still asymptomatic and only one child (six year old) with self-limiting SVTs after birth complained of short episodes of palpitations. Three of 5 cases of postnatal WPW-syndrome showed normalization of ECG on follow-up with disappearance of	Unmentioned	8 had complex congenital heart malformations

ventricular preexcitation. Interestingly, a new case of asymptomatic WPW-syndrome was found in a 9 year old child who had a normal ECG and self-limiting SVTs at birth. In cases of WPW-syndrome follow-up ECGs were recommended and instructions were given on how to act when symptoms occur. In the AF group, 5 of 6 cases remained free of arrhythmia symptoms. Four of 6 underwent neurological and cardiac examination. The ECG was normal in 3 of 4. One 2 year old AF case with postnatal WPW syndrome had remained asymptomatic but the ECG still showed ventricular preexcitation. A 9 year old AF case had developed drug refractory AVRT after birth and underwent successful catheter ablation at the age of 4 years. One 4-year old child with AF had coarctation of the aorta and self-limiting AF. The patient underwent coarctectomy and remained asymptomatic after surgery.

		Digoxin: confused reported			
Shah, A.	2012	The maximum sotalol dose among the treated pregnant women ranged from 80 mg 2 times/day to 240 mg 3 times/day.	8 treated fetuses had documented arrhythmias postnatally. Three of these infants were rapid responders in utero. The SVT mechanism was correctly predicted by the VA and AV interval assessment, with 1 infant with permanent junctional reciprocating tachycardia (long VA in utero), 1 with ectopic atrial tachycardia (long VA in utero), and 1 with AV node reentry tachycardia (short VA in utero). All 5 live-born fetuses with more difficult to treat SVT in utero (slow or partial responders) have had SVT that has been a challenge to treat after birth: 4 had AV reentry tachycardia (1 with Wolff-Parkinson-White syndrome and the others with concealed pathways), and 1 had permanent junctional reciprocating tachycardia. No fetus with AF had tachycardia postnatally.	The maternal symptoms during sotalol therapy included nausea, dizziness, and fatigue in 4 mothers who were concomitantly taking digoxin. No mothers developed torsades during sotalol therapy. In no cases was sotalol discontinued because of maternal side effects.	Mentioned but lack of data.
van der Heijden, L. B.	2012	The initial dosage of sotalol was 160– 320 mg daily in two to three doses. The initial dosage depended on body weight of the mother and presence or absence of fetal hydrops. In cases in which maternal body weight was < 100 kg, an initial dose of 80 mg twice daily was used in non-hydropic fetuses, and 80 mg three times daily was used in hydropic fetuses. The initial dosage in patients > 100 kg was 80 mg three times daily in non-hydropic fetuses and 160 mg twice daily in hydropic fetuses. In one case presenting with AF with a ventricular heart rate of 145 bpm, a lower initial dose of 40 mg twice daily was used to achieve cardioversion. In cases of persistence of tachycardia, the dosage of sotalol was increased to a maximum of 480 mg daily. The dosage of digoxin was 0.375– 1.000 mg daily in two to four doses. The initial dosage offlecainide was 100– 150 mg twice daily. The maximum dosage offlecainide was 400 mg daily. On at least every weekly visit, participants were interviewed to reveal potential adverse effects of the drug therapy.	Directly after birth SR was confirmed in all but two patients (93%). Antiarrhythmic drug therapy was not administered to these 26 patients. In the two patients without SR directly after birth (7%), rate control was achieved antenatally and the postpartum ECG showed AF. Electrical cardioversion established normal SR in one of these patients, and a maintenance dose of sotalol was discontinued after 3 months. In the other patient, several attempts to convert electrically failed, and a maintenance therapy of digoxin and sotalol was initiated. The patient converted within a few days. Digoxin was discontinued 2 months after birth and sotalol 8 months after birth. No recurrent tachycardias were detected in either case. In eight of the 26 infants with SR directly after birth (31%), SVT was observed 5 hours to 3 weeks postnatally. In the remaining 18 patients (69%; 11 with fetal SVT, seven with fetal AF) no rhythm disturbances were detected after birth and there were no events during follow-up (median, 54 (range, 19– 102) months). Five of the eight patients with recurrent atrial tachycardia showed SVT within 3 days after birth (63%). The antenatal rhythm diagnosis was concordant with the postnatal rhythm diagnosis in four of these patients (SVT) and discordant in one (with antenatal rhythm diagnosis of AF and postnatally diagnosis of SVT). One of	Maternal adverse effects of sotalol therapy were encountered in 15 cases and consisted of dizziness (n = 11), fatigue (n = 4), nausea and vomiting (n = 3), headache (n = 3) and dyspnea (n = 1). In most cases, symptoms were minor. In two patients, one using sotalol as a single drug therapy and the other using a combination of sotalol and flecainide (as outlined above), the reported adverse effects diminished with lowering of the sotalol dosage from 80 mg twice daily to 40 mg twice daily after SR was achieved. Drug changes were not required. There were no serious side effects such as fainting, syncope or arrhythmia. Adverse effects occurred independent of drug dosage and gestational age and were not associated with addition of a second agent. In two cases, delivery was induced at term, partly because of maternal adverse	Excluded

			these five children (antenatal diagnosis concordant with postnatal diagnosis) was diagnosed with atrioventricular re-entry tachycardia based on Wolff−Parkinson−White syndrome. This child had one relapse at the age of 3 months. A maintenance dose of sotalol therapy was stopped 15 months after birth without recurrence of tachycardia (follow-up at 31 months). Four patients received sotalol in a maintenance dose for 8– 12 months, after which treatment was discontinued successfully without recurrent signs of tachycardia. Three of the eight patients developed atrial tachycardia 2– 3 weeks after birth (37%). One was diagnosed with ectopic atrial tachycardia and at the time of writing is doing well on antiarrhythmic therapy with sotalol (last follow-up at 46 weeks). In the other two patients, a maintenance dose of sotalol was discontinued after 12 months. No relapses occurred. Significant neurological morbidity was present after birth in one patient, presenting at 36 + 5 weeks with SVT at 280 bpm with fetal hydrops (ascites, skin edema, pericardial effusion). Antenatal treatment with sotalol achieved SR within 2 days. The infant was born at 37 + 1 weeks' gestation with normal Apgar scores and blood gases after a spontaneous vaginal delivery. SVT occurred 5 hours after birth. Cardioversion with adenosine was successful and maintenance therapy with sotalol was initiated. During postnatal evaluation, magnetic resonance imaging depicted a large infarct of the left medial cerebral artery with hemorrhagic components due to venous thrombosis of the transverse sinus. Based on imaging, these abnormalities most likely originated from at least 6 weeks before delivery and may have resulted from low cardiac output. There was no fetal or postnatal mortality.	effects. Both patients, respectively reporting dizziness and dizziness combined with nausea and vomiting, converted antenatally to SR with sotalol as a single therapy.	
Uzun, O.	2012	D: 19.6±0.92 nmol/L<br>1.94±0.90 nmol/L for D, 0.51±0.19±0.19 mg/L for F<br>F: 0.70±0.40 mg/L	A severe vein of Galen aneurysm complicated one case and the patient opted for termination of her pregnancy. There was one neonatal death due to severe pulmonary hypoplasia and gross foetal ascites. In re-entry tachycardia, 1 foetus who did not respond to any treatment antenatally showed sinus rhythm at birth. However,	1 maternal hypothyroidism but adequately treated; 5 has nausea, headache, tiredness, and loss of appetite. 1 patient complained of sickness and visual symptoms related to digoxin	1 has Ebstein's anomoly of the tricuspid valve; Postnatally, 3 were found

			tachycardia recurred a few hours after birth, and adenosine effectively terminated the tachycardia. Sinus rhythm was maintained on amiodarone infusion, but the newborn died due to severe respiratory distress. In all, seven newborns developed atrioventricular re-entry tachycardia beyond 24 hours of age and needed anti-arrhythmic medication. Out of the seven cases of atrial flutter, four neonates were noted to be in atrial flutter at birth, and therefore they were treated with direct current cardioversion to restore the sinus rhythm. Anti-arrhythmic medication was needed in two newborns because of the emergence of atrioventricular re-entry tachycardia– in one patient a few hours after restoration of sinus rhythm by direct current cardioversion, and in the other a week after delivery. A total of nine children– seven with re-entry supraventricular tachycardia and two with atrial flutter– required postnatal anti-arrhythmic treatment. In seven children, anti-arrhythmic medication was discontinued within a year. Only two children remained on anti-arrhythmic medication in the follow-up beyond the first year of age. After birth, three newborns exhibited pre-excitation – Wolff–Parkinson–White Syndrome– two of whom had recurrence of atrioventricular re-entry tachycardia and required treatment for a few months. Both patients remained arrhythmia free after discontinuation of treatment. A third patient has continued to show asymptomatic pre-excitation on a 12-lead electrocardiogram, but never developed arrhythmia following termination of atrial flutter with direct current cardioversion. There is only one child with learning difficulties who also has a ventriculoperitoneal shunt for congenital hydrocephalus. It would be more likely that the neurological abnormalities were related to his hydrocephalus rather than foetal tachycardia.	despite the dose being in therapeutic range– 1.32 nmol/L. Deranged liver function test in 1 patient was caused by obstetric cholestasis. Upon reduction of the offending drug dose, all of the above-mentioned side effects resolved rapidly.	small muscular ventricular septal defects but closed spontaneously in the follow-up; 2 atrial septal defects postnatally; 1 hydrocephalus with aqueduct stenosis and hypoplastic cerebellum
Ekman-Joelsson, B. M.	2015	Transplacental digoxin treatment usually started with an oral loading dose of 1.5–2.0 mg given on a 24- to 36-hour basis, followed by a daily maintenance dose of	Termination of arrhythmia was associated with not having a Caesarean section, a longer time delay from diagnosis to delivery and a later gestational age at delivery. Preterm delivery was	Side effects were reported by 21 of the 99 women who received antiarrhythmic drugs: ten who were prescribed digoxin,	Excluded

approximately 0.5 mg to obtain a maternal drug level in the upper therapeutic range. Cases with severe hydrops frequently received an intravenous loading dose of 1.0 mg over a 24-hour period, followed by injections of 0.25–0.5 mg on days two and three. Sotalol treatment routinely started with an oral dose of 80 mg twice daily and increased to 160 mg twice daily within three days, depending on the degree of foetal hydrops. In rare cases, it was increased to a maximum dosage of 160 mg three times a day. Flecainide treatment started with a dose of 100 mg two to three times a day and increased to a maximum dosage of 400 mg per day.

observed in 28% of the cases and three-quarters of the 12% delivered before 35 weeks of gestation had a hydropic foetus at presentation. Two-thirds of our foetuses were boys, and the girls had a higher rate of intrauterine conversion (86 versus 63%, $p < 0.05$) and a lower degree of postnatal arrhythmia (24 versus 55%, $p < 0.01$). Neonatal neurological morbidity was observed in three cases. In one case, this was due to a postnatal cerebral haemorrhage, and in the other two cases, this was due to prenatal cerebral infarction, probably secondary to thromboembolism. Postnatal arrhythmia was seen in cases that did not respond to intrauterine treatment and was also seen in 25% of those with cardioversion (Table 4). Electroconversion was used in two cases with AF and one with AVRT. There was a small difference in the time from diagnosis to delivery between patients with AF and AVRT (5.1 ± 4.4 versus 7.5 ± 5.4 weeks, $p < 0.05$), but they were delivered at the same gestational age and all other neonatal outcome measures were similar. Postnatal arrhythmias were observed within two weeks of delivery in 19/38 (50%) cases with AF, 31/85 (36%) with AVRT, 1/3 with AET, 2/4 with PJRT, 1/2 with JET and none of the four cases with CAT or VT. In addition to the two severe cardiac malformations diagnosed prenatally, two small ventricular septal defects and one atrial septal defect were found in another three cases with AF. Postnatal antiarrhythmic treatment was started in 94 (70%) patients and treatment was more frequently provided in cases with foetal hydrops than those who were nonhydropic (87 versus 68%, $p < 0.05$), but there was no difference in frequency between AF and AVRT cases. The duration of treatment was typically six (1–12) months, 11 (8%) still had recurrence of arrhythmia after one year of age and six were treated with radiofrequency ablation. One case with PJRT and poor ventricular function at diagnosis had cardioversion and functional normalisation after birth, but arrhythmia recurred, accompanied by ventricular deterioration, and a cardiac transplant was carried out at

three who used sotalol and eight who used a combination of both. In two cases, preeclampsia with decreased renal function and severe digoxin intoxication developed within 24 hours of treatment and they were delivered by Caesarean section at 32 and 33 weeks of gestation. One woman needed intensive care treatment, but both the women and their babies survived without sequelae. Other less dramatic side effects were nausea, vomiting, tiredness, visual impairment, dizziness and loss of sensation.

			eight years of age. Neurological sequelae were present infive cases. Two of the three with neurological symptoms at birth had hemiparesis, but no sequela was documented in the third. Another two children had a mild neurodevelopmental delay and afifth had an unclear neurological disease, with paraparesis, and died atfive months of age. Tumours were diagnosed in two infants and one had a lethal metabolic disease. There werefive postnatal deaths, including one at two months of age, which was thought to be due to a cardiac issue due to an arrhythmia, as the child had AVRT and was on amiodarone prophylaxis.		
Sridharan, S.	2016	At center 1, 34 outpatient mothers received oralflecainide, usually at an initial dose of 300 mg daily in 3 separate doses. A maternal electrocardiogram was recorded prior to the initiation of treatment. Follow-up was arranged in 1–7 days from treatment onset according to physician judgment. Flecainide dose was decreased to 100 mg twice daily or less following conversion to fetal sinus rhythm (SR). If tachycardia persisted atfirst review, or if the dose was increased subsequently, maternal plasma level (trough) was requested. Addition of amiodarone or digoxin was considered if reversion to SR was not achieved. At center 2, 50 hospital inpatient mothers received intravenous digoxin in a protocol proposed by the Fetal Working Group of the Association of European Paediatric Cardiology.8 The initial digoxin dose was 1.5 mg/24 hours in 3 divided doses, increasing up to 2.0 mg/24 hours in 2 divided doses if required. Digoxin was administered intravenously in short continuous infusions until a maternal plasma level of 2.0–3.0 ng/mL was achieved. Maternal plasma levels and electrocardiogram were checked daily at the initial phase of treatment. Once the therapeutic plasma level	At center 1, a hydropic fetus with short VA tachycardia, successfully treated withflecainide, required neonatal repair of esophageal atresia and tracheoesophageal fistula and subsequent cardiac surgical repair of tetralogy of Fallot. The infant died aged 7 months, probably because of aspiration, having not receivedflecainide since the neonatal period without observed recurrence of AVRT. This death was not attributed toflecainide exposure. A fetus with variable, but predominantly long, VA time intervals achieved intermittent SR and rate control prenatally. However, multifocal atrial tachycardia persisted for several months postnatally before sustained SR was achieved. There was intrauterine growth retardation, microcephaly, and intracranial calcification. Postnatal IgG and IgM antibodies to cytomegalovirus were detected. Another fetus with long VA tachycardia achieved rate control, but hydrops persisted, necessitating delivery at 38 weeks' gestation. Neonatal ventilatory, inotropic, and antiarrhythmic support was required, but the long-term outcome was good, with sustained SR off treatment. A third fetus with long VA tachycardia achieved rate control within 10 days offlecainide treatment onset, with reversion to SR occurring as late as 80 days later. SR was sustained off treatment postnatally. At center 2, no deaths were observed in nonhydropic fetuses. Intrauterine or neonatal death occurred in 7 of 9 (78%) of those with hydrops not	At center 2, 1 mother whose fetus was found to have a short VA SVT at 34 weeks' gestation did not tolerate digoxin. Sustained tachycardia persisted until 35 weeks' gestation when the fetus was delivered. Digoxin was otherwise well tolerated, despite the relatively high doses used. In another case of short VA SVT and hydrops, the mother did not tolerate treatment. She suffered from psychiatric illness and pregnancy was terminated at 22 weeks of gestation. At center 1, symptoms that might have been related to flecainide occurred in 8 of 34 mothers (24%): in 7, lightheadedness, nausea, headache, or transient blurred vision was reported, and 1 mother described a sensation of heightened alertness. None of these symptoms required cessation of flecainide treatment. Only 1 mother had measured plasma flecainide level greater than 700 µg/L. This was a mother in whom flecainide dose was increased from	Mentioned but lack of data.

		was obtained, digoxin was administered orally if treatment had been successful, or a second-line drug (usually sotalol) was introduced.	responding to therapy. Interestingly, intrauterine death also occurred in 2 of 12 hydropic fetuses (17%) that had responded to therapy but presented with severely impaired myocardial function.	100 mg 3 times daily (plasma level 310 µg/L) to 100 mg 6 hourly (plasma level 910 µg/L), which was effective in restoring SR in a hydropic fetus.	
Strizek, B.	2016	β-methyldigoxin was administered as a loading dosage of 800–1000 mg/d (in 4 doses) for 3 days and continued by a maintenance dosage of 500–600 mg/d. If no treatment response was noted after the loading dose phase, serum levels were evaluated and treatment adjusted to achieve levels of 2.0–2.5 ng/mL. Flecainide was administered 100 mg 4 times daily as a loading dosage in hydropic fetuses for 2–3 days and continued with 300 mg/d. In the absence of hydrops, the initial dosage was 300 mg/d. In 1 patient, amiodarone (Cordarex) was added up to 2000 mg/d orally (loading dosage for 4–5 days followed by a maintenance dosage of 400–800 mg/d). Maternalflecainide and amiodarone serum levels were not evaluated.	No follow-up were described	For flecainide group:<br>In 1 asymptomatic mother with tuberous sclerosis, there was a Brugada pattern on the ECG under flecainide therapy, which disappeared after cessation of flecainide. The fetus, which had several cardiac rhabdomyomas, had converted to SR after 6 days and remained in SR even without further treatment. Because of the molecular genetic diagnosis of fetal tuberous sclerosis, the pregnancy was eventually terminated. No other maternal side effects or ECG abnormalities were noted.<br>For digoxin group:<br>One mother had visual symptoms that required reduction of dosage of digoxin, and 2 patients showed first-degree AV block that was reversible after reduction of digoxin	As additional cardiac findings, 2 fetuses had rhabdomyomas in the context of tuberous sclerosis, 1 fetus had a muscular ventricular septal defect (VSD), and 1 had pulmonary atresia with VSD. 1 fetus showed agenesis of the ductus venosus.

Ekiz, A.	2017	Flecainide was initially administered to the mother in an oral dose of 300 mg daily which was given 100 mg three times a day. The maximum dose of flecainide was 400 mg daily. If sinus rhythm was not obtained via flecainide monotherapy within 7 days, the treatment was combined with digoxin. Digoxin therapy was started with a loading dose of 1.5 to 2 mg over 2 days. Digoxin was continued with the dosages between 0.375 and 1mg/day, aiming for between 1.5 and 2.5 ng/mL of digoxin levels.	The newborns were followed up with a mean time of 22.8 months (range 6-48). Although, SR was achieved with antiarrhythmic treatment one fetus was delivered at 32 weeks of gestation, because of having severe signs of hydrops and non-reassuring fetal status. This fetus was the only neonatal death of our series. Five newborns required antiarrhythmic therapy. Only 2 of these were treated prenatally and remaining 3 fetuses did not receive treatment with flecainide because one fetus with AF required prompt delivery due to non-reassuring fetal status and 2 had rhabdomyomas along with intermittent SVT.	One mother developed atrial fibrillation one week after flecainide treatment had started; fortunately, spontaneous resolution of atrial fibrillation was observed after flecainide treatment had been given up. Also of interest: the fetus did not need antiarrhythmic treatment any longer. Additionally, one mother complained of dizziness, but there was no need to cancel or change the therapy. We have not experienced any other adverse reaction to flecainide treatment, including visual disturbances, nausea and palpitation.	2 fetuses had cardiac rhabdomyomas ; hence, they had the diagnosis of tuberous sclerosis after birth.
Karmegeraj, B.	2018		In the SVT group, one infant had a recurrence while on propranolol triggered by salbutamol nebulization. This responded to adenosine and the beta-blocker was to bisoprolol. The median duration of follow-up was 10.5 (3–64) months in the SVT group and the median duration of maintenance therapy was 5 weeks (3–16). In the AF group, were no complications or recurrence on follow-up. The median duration of follow-up was 4 (1–19) months and duration of maintenance antiarrhythmic therapy was 3 (1–19) weeks. Details of comparison between SVT and AF groups were presented in original article in form of table.		

Miyoshi, T.	2019	Rapid initial saturation was performed with a 0.5mg intravenous injection. Intravenous injections of 0.25 mg were administered at 8 and 16 h after the initial dose. If intravenous injections were not feasible, oral digoxin (1.5 mg/day) was given in 3 divided doses. Subsequently, oral digoxin at 0.75 mg/day was given in 3 divided doses, with adjustment to maintain maternal serum concentrations from 1.5 to 2.0 ng/ml. Sotalol at 160 mg/day in 3 divided doses was added. If a regimen of 3 days of treatment at 160 mg/day of sotalol was ineffective, the patient then proceeded to 240 mg/day in 2 divided doses. If 240 mg/day was ineffective after 3 days of treatment, the patient proceeded to 320 mg/day in 2 divided doses.	Fetal AEs related to transplacental treatment were observed in 12 fetuses (24.0%). Serious AEs resulting in discontinuation of the protocol-treatment occurred in 4 fetuses. Fetal death occurred in 2 of 49 fetuses overall (4.1%; 95% CI: 0.5% to 14.0%). In 1 fetus heavy for gestational age (>5.0 SD), AFL developed with ascites, cardiac effusion, and polyhydramnios at 26 weeks of gestation. After digoxin and sotalol combination therapy, the frequency and ventricular rate of fetal AFL decreased, but hydrops progressed, resulting in fetal death at 27 weeks of gestation. Postmortem examination showed hypoplastic lungs, small ears, and flexion of the long finger in both hands, findings suggesting Costello syndrome. Another fetus of 34 weeks of gestation with a diagnosis of short VA SVT once achieved sinus rhythm and resolved pleural effusion and ascites after digoxin and sotalol, but the tachyarrhythmia recurred at 36 weeks of gestation. SVT was sustained even after increased dosage of sotalol to 240 mg/day, and the fetus developed pleural effusion and ascites. The fetus died at 37 weeks of gestation during preparations for cesarean section. These fetal deaths were mainly caused by progression of fetal heart failure secondary to AFL and SVT. In 1 fetus with AFL, 7:1 AV block was observed after starting the combination of digoxin and sotalol. Because the ventricular rate decreased to 50 beats/min for 5 min at 36 weeks of gestation, cesarean section was performed, and the newborn was treated by electrical cardioversion. In another fetus with AFL, 1:1 AV conduction at 275 beats/min was observed 5 days after starting the combination of digoxin and flecainide. The fetus progressed to ascites and pleural effusion and was delivered by cesarean section at 32 weeks of gestation. AFL resolved spontaneously just after birth.	Although maternal AEs related to transplacental treatment were observed in 39 patients (78.0%), there was only 1 serious event: Mobitz type II AV block was observed but resolved immediately after temporary discontinuation of digoxin and sotalol. Nausea or vomiting, the most common maternal adverse symptom, was observed in 27 patients (54.0%). Electrocardiographic abnormalities were detected in 19 patients (38.0%). Elevated brain natriuretic peptide concentrations were found in 25 patients (50.0%). Despite a relatively high incidence of maternal AEs, dose reduction allowed for continuation of transplacental treatment.	Excluded

O'Leary , E. T.	2020	Digoxin is administered as a 1-g oral load over 24 hours in three divided doses (eg, 0.5, 0.25, and 0.25 mg). Maintenance dosing is titrated to target a maternal serum level of 1 to 2 ng/mL. Initial flecainide dosing is 100 mg by mouth, three times per day, with further dose adjustments dictated by fetal response and maternal serum flecainide levels (>250 mcg/L but <1000 mcg/L). Initial sotalol dosing is 80 mg by mouth, three times per day; sotalol levels are not routinely checked.	Neonatal records were available for review in 61 of 65 (94%) cases of fetal SVT, with 31 of these infants having a documented episode of clinical SVT at a median age of 0 days (0-62). Manifest pre-excitation was present in eight neonates. Patients had between zero and five episodes of documented SVT with 30 (49%) having no episodes and six (10%) having two or more episodes. The latest presentation of accessory pathway-mediated SVT (ie, AVRT or PJRT) was 39 days of life. All of the seven infants with postnatal AFL presented on the first day of life, underwent successful esophageal pace termination or direct current cardioversion, and had no additional episodes beyond this. One infant with a history of fetal EAT had a first postnatal episode of EAT at 62 days of life. Two neonatal deaths occurred during the study period. One female neonate was diagnosed with fetal AVRT at 21 weeks gestation and remained in incessant SVT for 2 weeks on digoxin monotherapy before transfer to Brigham and Women's Hospital. Flecainide and sotalol monotherapy, as well as intraumbilical adenosine administration, were all tried without successful conversion. Amiodarone and flecainide were started in combination at 26 weeks gestation which ultimately resulted in adequate ventricular rate control (~170 bpm). Unfortunately, progressive hydrops with severe ventricular dysfunction developed and an emergent Cesarean delivery was performed at 33 weeks gestation following a fetal abdominal paracentesis. She expired on the third day of life from multiorgan failure and the cardiac exam on autopsy demonstrated histologic evidence of a left-sided accessory pathway. The second death occurred in a male neonate diagnosed with fetal AVRT at 20 weeks gestation. After failing to respond to flecainide monotherapy, conversion to sinus rhythm was achieved with amiodarone and flecainide in combination at 23 weeks gestation. He was delivered at 30 weeks gestation and 1.2 kg via elective Cesarean delivery due to the unremitting hydrops and severe ventricular dysfunction. Recurrent postnatal SVT and	Unmentioned	Excluded

cardiorespiratory failure ensued, thus an intracardiac EPS was performed on day 1 of life where a left posterior accessory pathway was ablated with acute success. Unfortunately, SVT recurred on the 6th day of life (with minimal hemodynamic impact) and support was ultimately withdrawn due to significant multiorgan dysfunction at 4 weeks of life Follow-up beyond the neonatal hospitalization was available in 56 of 61 (92%) patients with a median follow-up time of 1.6 years (range, 0.02-16.6) and of similar duration among the SVT subtypes. Twenty-nine infants were discharged from the neonatal hospitalization on maintenance AAD therapy. Drug therapy was discontinued in 20 of 29 infants at a median age of 1.0 years (range, 0.3-7.0). Three patients with AVRT experienced recurrent SVT following AAD discontinuation and all have since undergone successful radiofrequency ablation. Seven infants remained on AAD therapy beyond 1 year of life, with six presently on AAD therapy and one having undergone successful radiofrequency ablation for PJRT. Two infants under 1 year of age remain on AAD therapy at the time of publication. Intracardiac EPS and ablation were performed in a total of six patients (four AVRT, two PJRT) at a median age and weight of 4.5 years (range, 0.002-14.0) and 19.9 kg (range, 1.2-61.1), respectively. Overall, 44 (79%) of the 56 patients with postnatal follow-up were arrhythmia-free, off maintenance AAD therapy, and without the need for ablation by 1 year of life at the time of most recent follow-up.

Tunca Sahin, G.	2021	Except for one case, high doses of flecainide and digoxin combination treatment was given to all patients. Loading doses of digoxin were given on the first day of treatment with an initial dose of 500 µg followed by 250 µg, and 250 ug doses every 8 h. Flecainide was initiated concomitantly at the dose of 100 mg three times a day. The antiarrhythmic doses of each medication were adjusted according to maternal tolerance, drug serum levels, electrocardiogram, and fetal clinical response to tachycardia.	Of these sixteen cases, ten babies (62.5%) were born in sinus rhythm. Six babies (37.5%) were in atrial flutter at birth (with variable atrioventricular block) and were treated with DC cardioversion to restore sinus rhythm. Two patients remained in sinus rhythm, but four patients had AVRT immediately after DC cardioversion development, and AVRT was observed in one patient on a Holter recording a week after delivery. These five patients (31.3%) required postnatal antiarrhythmic treatment for up to 2 years. All patients remained arrhythmia-free after discontinuation of treatment. There was no recurrence of atrial flutter in the long term. The ECGs of four patients with AVRT exhibited pre-excitation, which disappeared in two patients during follow-up (Fig. 1). One patient continued to show asymptomatic pre-excitation on ECG but did not develop arrhythmia following termination of atrial flutter with DC cardioversion. RFA was successfully performed at the age of 6 years. Intriguingly, during the RFA procedure this patient developed multiple episodes of preexcited atrial fibrillation with rapid ventricular response requiring repeat DC cardioversion. One asymptomatic patient was referred for RFA after stopping medication owing to persistent pre-excitation at 8 years of age (Fig. 2). The median duration of follow-up was 4.5 years (range 0.6–16). No neurological morbidity was documented in the surviving neonates.	Unmentioned	Excluded

Data extraction of included studies in SVT group

Author	Year	Treatment for SVT	Total for SVT	Cardioversion for SVT
I. M. FROHN-MULDER	1995	D	22	12
		DF	4	4
E.Naumburg	1997	D	4	1
		DF	1	0
		DV	2	0
E.S. Ebenroth	2001	D	37	17

		DF	13	12
		SF	1	1
J.M. Jouannic	2002	A	5	2
		D	32	26
		DA	1	1
		DS	1	1
		S	1	1
M.Krapp	2002	D	13	4
		DF	7	7
Boldt	2003	D	26	14
		DS	3	2
		DF	3	2
Jean-Marie Jouannic	2003	A	4	2
		D	7	0
		DS	2	0
		F	12	7
Martjin A. Oudijk	2003	DS	2	2
		S	7	6
Michele D’Alto	2008	D	4	4
		DS	2	2
		FD	3	3
Pézard	2008	D	8	7
		F	1	0
Raika LuRajka Lulic ´ Jurjevic	2009	D	2	2
		DF	1	1
		DS	2	2
		F	6	3
Nathan D. Hahurij	2010	D	7	2
		F	3	3
		FD	2	1
		S	4	3
Amee Shah	2012	S	4	3

		SD	12	9
L. B. VAN DER HEIJDEN	2012	F	2	2
		S	14	14
		SD	1	1
		SF	3	3
Orhan Uzun	2012	D	6	0
		F	1	0
		FD	15	14
Ekman-Joelsson	2015	D	30	10
		DS	28	13
		F	2	2
		S	25	15
Shankar Sridharan	2016	D	50	31
		F	34	33
Ali Ekiz	2017	DF	1	1
		F	16	15
		FDS	1	0
Balaganesh Karmegeraj	2018	D	3	3
		DF	6	5
Takekazu Miyoshi	2019	D	15	7
		DS	2	1
		S	4	4
Edward T. O'Leary	2020	D	35	25
		F	3	2
		S	1	0

D, Digoxin; DF, Digoxin and Flecainide; DS, Digoxin and Sotalol; F, Flecainide; S, Sotalol; DV, Digoxin and Verapamil; DA, Digoxin and Amiodarone; A, Amiodarone; DFS, Digoxin, Flecainide and Sotalol; SF, Sotalol and Flecainide

Data extraction of included studies in AF group

Author	Year	Treatment for AF	Total for AF	Cardioversion for AF
E.Naumburg	1997	D	6	3
		DV	2	0

Lukas A. Lisowski	2000	D	21	16
		DS	5	4
		S	9	8
Martjin A. Oudijk	2003	DS	2	0
		S	7	7
Nathan D. Hahurij	2010	D	1	1
		F	1	1
		S	1	0
L. B. VAN DER HEIJDEN	2012	S	8	8
		SD	2	0
Ekman-Joelsson	2015	D	16	9
		DS	4	1
		S	7	4
Balaganesh Karmegeraj	2018	D	2	2
		DF	3	3
		DS	3	3
Takekazu Miyoshi	2019	D	27	16
		D	11	8
		D	2	1
		DS	1	0
Edward T. O'Leary	2020	D	6	2
		F	3	3
		S	2	2
Gulhan Tunca Sahin	2021	DF	12	9
		F	1	0

D, Digoxin; DF, Digoxin and Flecainide; DS, Digoxin and Sotalol; F, Flecainide; S, Sotalol; DV, Digoxin and Verapamil; DA, Digoxin and Amiodarone; A, Amiodarone; DFS, Digoxin, Flecainide and Sotalol; SF, Sotalol and Flecainide

Data extraction of included studies in Hydrops group

Author	Year	Treatment for hydrops	Total for hydrops	Cardioversion for hydrops
van Engelen	1994	D	10	1
		F	5	2

I. M. FROHN-MULDER	1995	D	6	1
		F	7	3
Lukas A. Lisowski	2000	D	16	11
		S	1	0
Martjin A. Oudijk	2000	DS	4	3
		S	4	2
M.Krapp	2002	D	10	1
		DF	6	6
Jean-Marie Jouannic	2003	A	4	2
		D	7	0
		DS	2	0
		F	12	7
Michele D’Alto	2008	D	2	1
		DF	3	2
Pézard	2008	D	4	1
		F	1	0
Nathan D. Hahurij	2010	D	2	0
		DF	2	1
		F	2	2
		S	3	3
Boldt	2003	D	6	0
		DF	1	1
L. B. VAN DER HEIJDEN	2012	F	2	2
		S	4	4
		SF	2	2
Ekman-Joelsson	2015	D	17	4
		DS	19	6
		F	1	1
		S	14	6
Sridharan	2016	D	21	9
		F	7	7
Strizek	2016	D	3	0

		DF	3	3
		F	18	12
Ali Ekiz	2017	DF	1	1
		DFS	1	0
		F	14	13
Takekazu Miyoshi	2019	DS	3	1
		S	1	1
Gulhan Tunca Sahin	2021	DF	5	4
		F	1	0

D, Digoxin; DF, Digoxin and Flecainide; DS, Digoxin and Sotalol; F, Flecainide; S, Sotalol; DV, Digoxin and Verapamil; DA, Digoxin and Amiodarone; A, Amiodarone; DFS, Digoxin, Flecainide and Sotalol; SF, Sotalol and Flecainide

Data extraction of included studies in Non-hydrops group

Author	Year	Treatment for non-hydrops	Total for non-hydrops	Cardioversion for non-hydrops
van Engelen	1994	D	14	10
		F	5	5
I. M. FROHN-MULDER	1995	D	22	12
		DF	4	4
Lukas A. Lisowski	2000	D	5	5
		DS	5	4
		S	8	8
Martjin A. Oudijk	2000	DS	3	2
		S	8	7
J.M. Jouannic	2002	A	5	2
		D	32	26
		DA	1	1
		DS	1	1
		S	1	1
M.Krapp	2002	D	3	3
		DF	1	1
Boldt	2003	D	26	14
		DF	3	2

		DS	3	2
Michele D'Alto	2008	D	4	4
		DF	3	3
		DS	2	2
Pézard	2008	D	12	6
		F	1	1
Raika LuRajka Lulic ´ Jurjevic	2009	D	2	2
		DF	1	1
		DS	2	2
Nathan D. Hahurij	2010	D	6	5
		F	2	1
		S	2	1
Amee Shah	2012	DS	4	4
		S	9	8
L. B. VAN DER HEIJDEN	2012	DS	3	1
		S	18	18
		SF	1	1
Orhan Uzun	2012	D	6	0
		DF	13	11
		F	1	0
Ekman-Joelsson	2015	D	29	15
		DS	13	8
		F	1	1
		S	18	13
Shankar Sridharan	2016	D	29	23
		F	27	26
Strizek	2016	D	11	2
		DF	3	3
		F	10	9
Takekazu Miyoshi	2019	D	42	25
		S	3	3

D, Digoxin; DF, Digoxin and Flecainide; DS, Digoxin and Sotalol; F, Flecainide; S, Sotalol; DV, Digoxin and Verapamil; DA, Digoxin and Amiodarone; A, Amiodarone; DFS, Digoxin, Flecainide and Sotalol; SF,

Sotalol and Flecainide

Code

```
library("gemtc")
library("rjags")
library('ggplot2')
library('igraph')

###
data <- read.csv("safety.csv", sep=",", header=T)
id <- read.csv("id.csv", sep=",",header=T)
```

num	study	treatment	sampleSize(cardioversions in total group)	Responders(Intrauterine death in total group)
1	2	S	8	0
2	2	DS	9	3
3	4	DF	7	0
4	4	D	4	0
5	5	DS	1	0
6	5	S	4	0
7	5	D	25	0
8	6	DS	5	1
9	6	S	9	3
10	7	D	5	0
45	21	D	13	1

Data could be accessed in above tables, the order of data may be adjusted.

id	description
D	Digoxin
DF	Digoxin+Flecainide
F	Flecainide

DS	Digoxin+Sotalol

```
###network
network <- mtc.network(data, description="Example", treatments=id)
###networkplot
plot(network,vertex.color=c('#FFF5EE','#FFDAB9','#F4A460','#D2691E','#8B4513'),
        vertex.label.color='black',
        vertex.label.dist=2.5,
        edge.color='#DCDCDC')
###forest
model <-mtc.model(network, type = "consistency", factor = 2.5, n.chain = 4,linearModel="random")
results <- mtc.run(model, sampler = NA, n.adapt = 5000, n.iter = 20000, thin = 1)
###forestplot
summary(results)
forest<-summary(relative.effect(results, "D"))
summary(forest)
###forestggplot
forest_data<-data.frame(forest$summaries$quantiles)
forest_data<-forest_data[-5,]
forest_data$name<-c('DF','F','DS','S')
ggplot(data=forest_data)+
  aes(x=X50.,y=name)+
  geom_errorbarh(aes(xmax=X97.5.,xmin=X2.5.),color='black',height=0.2,size=0.8,alpha=0.3)+
  geom_point(size=4,shape=18)+
  geom_vline(xintercept = 1,linetype='dashed',size=1.2)+
  coord_trans(xlim=c(-8,5))+
  scale_x_continuous(breaks=c(-8,-6,-4,-2,0,2,4,6))+
  labs(x='Odd Ratios',y='Treatment', title = 'Forest Plot (Compared with Digoxin)', subtitle = 'Safety Index')
###assessing model convergence
plot(results)
gelman.plot(results)
gelman.diag(results)
###rank
```

```
ranks<- rank.probability(results,preferredDirection = 1)
print(ranks)
###rankplot
plot(ranks, beside=TRUE)
###rankggplot
ranks_data<-data.frame(name=c('D','DF','DS','F','S'),rank1=ranks[,1],rank2=ranks[,2],rank3=ranks[,3],rank4=ranks[,4],rank5=ranks[,5])
ranks_data$rank2<-ranks_data$rank1+ranks_data$rank2
ranks_data$rank3<-ranks_data$rank3+ranks_data$rank2
ranks_data$rank4<-ranks_data$rank3+ranks_data$rank4
ranks_data$rank5<-ranks_data$rank4+ranks_data$rank5
ranks_data=melt(ranks_data,id="name")
ggplot(data = ranks_data, aes(x =variable, y = value, group=name)) +
  geom_line(aes(color=name),size=1.5)+
  geom_point(aes(colour=name), size=4, shape=21, fill="white")+
  labs(x='Rank',y='Cumulative Probability', title = "Possibility of Treatment Rank", subtitle = 'Safety Index')
ranks_data<-data.frame(name=c('D','DF','DS','F','S'),rank1=ranks[,1],rank2=ranks[,2],rank3=ranks[,3],rank4=ranks[,4],rank5=ranks[,5])
ranks_data=melt(ranks_data,id="name")
ggplot(data = ranks_data, aes(x = name, y = value, fill=variable)) +
  geom_bar(stat = "identity", position = "stack",width = 0.75) +
  labs(x = "Treatments", y = "Possibility", title = "Possibility of Treatment Rank", subtitle = 'Safety Index')
#theme(axis.title = element_text(size = 10), axis.text = element_text(angle = 45, size = 10))+
#theme(panel.background = element_blank(), axis.line = element_line(colour = "grey"))
###nodesplit
result <-mtc.nodesplit(network)
summary(result)
#names(result)
summary.ns <- summary(result)
#print(summary.ns)
#plot(summary.ns)
###nodesplitggplot
data_d<-summary.ns$dir.effect
data_d$effect<-c('direct','direct','direct','direct','direct','direct','direct','direct','direct')
data_i<-summary.ns$ind.effect
```

```
data_i$effect<-c('indirect','indirect','indirect','indirect','indirect','indirect','indirect','indirect','indirect')
data_di<-rbind(data_d,data_i)
data_n<-summary.ns$cons.effect
data_n$effect<-c('network','network','network','network','network','network','network','network','network')
data_din<-rbind(data_di,data_n)
data_p<-summary.ns$p.value
data_dinp<-merge(data_din,data_p,by.x = c('t1','t2'),by.y =c('t1','t2'))
data_dinp<-unite(data_dinp, name, t1, t2, sep= " vs ")
data_dinp<-unite(data_dinp, name, name, effect, sep= " - ")
data_dinp$p0='color'
n=1
for (i in data_dinp$p) {
  print(n)
  if (i>0.05){
    data_dinp[n,]$p0 = '>0.05'
    n<-n+1
  } else {
    data_dinp[n,]$p0 = '<0.05'
    n<-n+1
  }
}
ggplot(data=data_dinp)+
  aes(x=pe,y=name)+
  geom_errorbarh(aes(xmax=ci.u,xmin=ci.l),color='black',height=0.2,size=0.8,alpha=0.3)+
  geom_point(size=4,shape=18,aes(color=p0))+#
  geom_vline(xintercept = 1,linetype='dashed',size=1.2)+
  coord_trans(xlim=c(-10,10))+
  scale_x_continuous(breaks=c(-10,-8,-6,-4,-2,0,2,4,6,8,10))+
  labs(x='Odd Ratios',y='Treatment Comparison', title = 'Node-spliting Analysis of Inconsistency', subtitle = 'Safety Index')
```
