# Supplementary material for: Efficacy and Safety of Various First-Line Therapeutic Strategies for Fetal Tachycardias: A Network Meta-Analysis and Systematic Review
Source: Front Pharmacol. 2022 Jun 13;13:935455. doi: 10.3389/fphar.2022.935455 (PMC9235149; doi:10.3389/fphar.2022.935455)
Supplement: Supplementary file 3 [file DataSheet3.PDF]

Code Sharing

```
library("gemtc")
library("rjags")
library('ggplot2')
library('igraph')

###
data <- read.csv("safety.csv", sep=",", header=T)
id <- read.csv("id.csv", sep=",",header=T)
```

num	study	treatment	sampleSize(cardioversions in total group)	Responders(Intrauterine death in total group)
1	2	S	8	0
2	2	DS	9	3
3	4	DF	7	0
4	4	D	4	0
5	5	DS	1	0
6	5	S	4	0
7	5	D	25	0
8	6	DS	5	1
9	6	S	9	3
10	7	D	5	0
45	21	D	13	1

Data could be accessed in above tables, the order of data may be adjusted.

id	description
D	Digoxin
DF	Digoxin+Flecainide
F	Flecainide
DS	Digoxin+Sotalol

```
###network
network <- mtc.network(data, description="Example", treatments=id)
###networkplot
plot(network,vertex.color=c('#FFF5EE','#FFDAB9','#F4A460','#D2691E','#8B4513'),
        vertex.label.color='black',
        vertex.label.dist=2.5,
        edge.color='#DCDCDC')
###forest
model <-mtc.model(network, type = "consistency", factor = 2.5, n.chain =
4,linearModel="random")
results <- mtc.run(model, sampler = NA, n.adapt = 5000, n.iter = 20000, thin = 1)
###forestplot
```

```
summary(results)
forest<-summary(relative.effect(results, "D"))
summary(forest)
###forestggplot
forest_data<-data.frame(forest$summaries$quantiles)
forest_data<-forest_data[-5,]
forest_data$name<-c('DF','F','DS','S')
ggplot(data=forest_data)+
  aes(x=X50.,y=name)+
  geom_errorbarh(aes(xmax=X97.5.,xmin=X2.5.),color='black',height=0.2,size=0.8,alpha=0.3)+
  geom_point(size=4,shape=18)+
  geom_vline(xintercept = 1,linetype='dashed',size=1.2)+
  coord_trans(xlim=c(-8,5))+
  scale_x_continuous(breaks=c(-8,-6,-4,-2,0,2,4,6))+
  labs(x='Odd Ratios',y='Treatment', title = 'Forest Plot (Compared with Digoxin)', subtitle =
'Safety Index')
###assessing model convergence
plot(results)
gelman.plot(results)
gelman.diag(results)
###rank
ranks<- rank.probability(results,preferredDirection = 1)
print(ranks)
###rankplot
plot(ranks, beside=TRUE)
###rankggplot
ranks_data<-data.frame(name=c('D','DF','DS','F','S'),rank1=ranks[,1],rank2=ranks[,2],rank3=ranks[
,3],rank4=ranks[,4],rank5=ranks[,5])
ranks_data$rank2<-ranks_data$rank1+ranks_data$rank2
ranks_data$rank3<-ranks_data$rank3+ranks_data$rank2
ranks_data$rank4<-ranks_data$rank3+ranks_data$rank4
ranks_data$rank5<-ranks_data$rank4+ranks_data$rank5
ranks_data=melt(ranks_data,id="name")
ggplot(data = ranks_data, aes(x =variable, y = value, group=name)) +
  geom_line(aes(color=name),size=1.5)+
  geom_point(aes(colour=name), size=4, shape=21, fill="white")+
  labs(x='Rank',y='Cumulative Probability', title = "Possibility of Treatment Rank", subtitle =
'Safety Index')
ranks_data<-data.frame(name=c('D','DF','DS','F','S'),rank1=ranks[,1],rank2=ranks[,2],rank3=ranks[
,3],rank4=ranks[,4],rank5=ranks[,5])
ranks_data=melt(ranks_data,id="name")
ggplot(data = ranks_data, aes(x = name, y = value, fill=variable)) +
  geom_bar(stat = "identity", position = "stack",width = 0.75) +
  labs(x = "Treatments", y = "Possibility", title = "Possibility of Treatment Rank", subtitle = 'Safety
```

```
Index')
#theme(axis.title = element_text(size = 10), axis.text = element_text(angle = 45, size = 10))+
#theme(panel.background = element_blank(), axis.line = element_line(colour = "grey"))
###nodesplit
result <-mtc.nodesplit(network)
summary(result)
#names(result)
summary.ns <- summary(result)
#print(summary.ns)
#plot(summary.ns)
###nodesplitggplot
data_d<-summary.ns$dir.effect
data_d$effect<-c('direct','direct','direct','direct','direct','direct','direct','direct','direct')
data_i<-summary.ns$ind.effect
data_i$effect<-c('indirect','indirect','indirect','indirect','indirect','indirect','indirect','indirect','indir
ect')
data_di<-rbind(data_d,data_i)
data_n<-summary.ns$cons.effect
data_n$effect<-c('network','network','network','network','network','network','network','network
','network')
data_din<-rbind(data_di,data_n)
data_p<-summary.ns$p.value
data_dinp<-merge(data_din,data_p,by.x = c('t1','t2'),by.y =c('t1','t2'))
data_dinp<-unite(data_dinp, name, t1, t2, sep= " vs ")
data_dinp<-unite(data_dinp, name, name, effect, sep= " - ")
data_dinp$p0='color'
n=1
for (i in data_dinp$p) {
  print(n)
  if (i>0.05){
    data_dinp[n,]$p0 = '>0.05'
    n<-n+1
  } else {
    data_dinp[n,]$p0 = '<0.05'
    n<-n+1
  }
}
ggplot(data=data_dinp)+
  aes(x=pe,y=name)+
  geom_errorbarh(aes(xmax=ci.u,xmin=ci.l),color='black',height=0.2,size=0.8,alpha=0.3)+
  geom_point(size=4,shape=18,aes(color=p0))+#
  geom_vline(xintercept = 1,linetype='dashed',size=1.2)+
  coord_trans(xlim=c(-10,10))+
  scale_x_continuous(breaks=c(-10,-8,-6,-4,-2,0,2,4,6,8,10))+
```

```
    labs(x='Odd Ratios',y='Treatment Comparison', title = 'Node-spliting Analysis of Inconsistency',
subtitle = 'Safety Index')
```
