# Supplementary material for: Efficacy and Safety of Various First-Line Therapeutic Strategies for Fetal Tachycardias: A Network Meta-Analysis and Systematic Review
Source: Front Pharmacol. 2022 Jun 13;13:935455. doi: 10.3389/fphar.2022.935455 (PMC9235149; doi:10.3389/fphar.2022.935455)
Supplement: Supplementary file 4 [file DataSheet1.PDF]

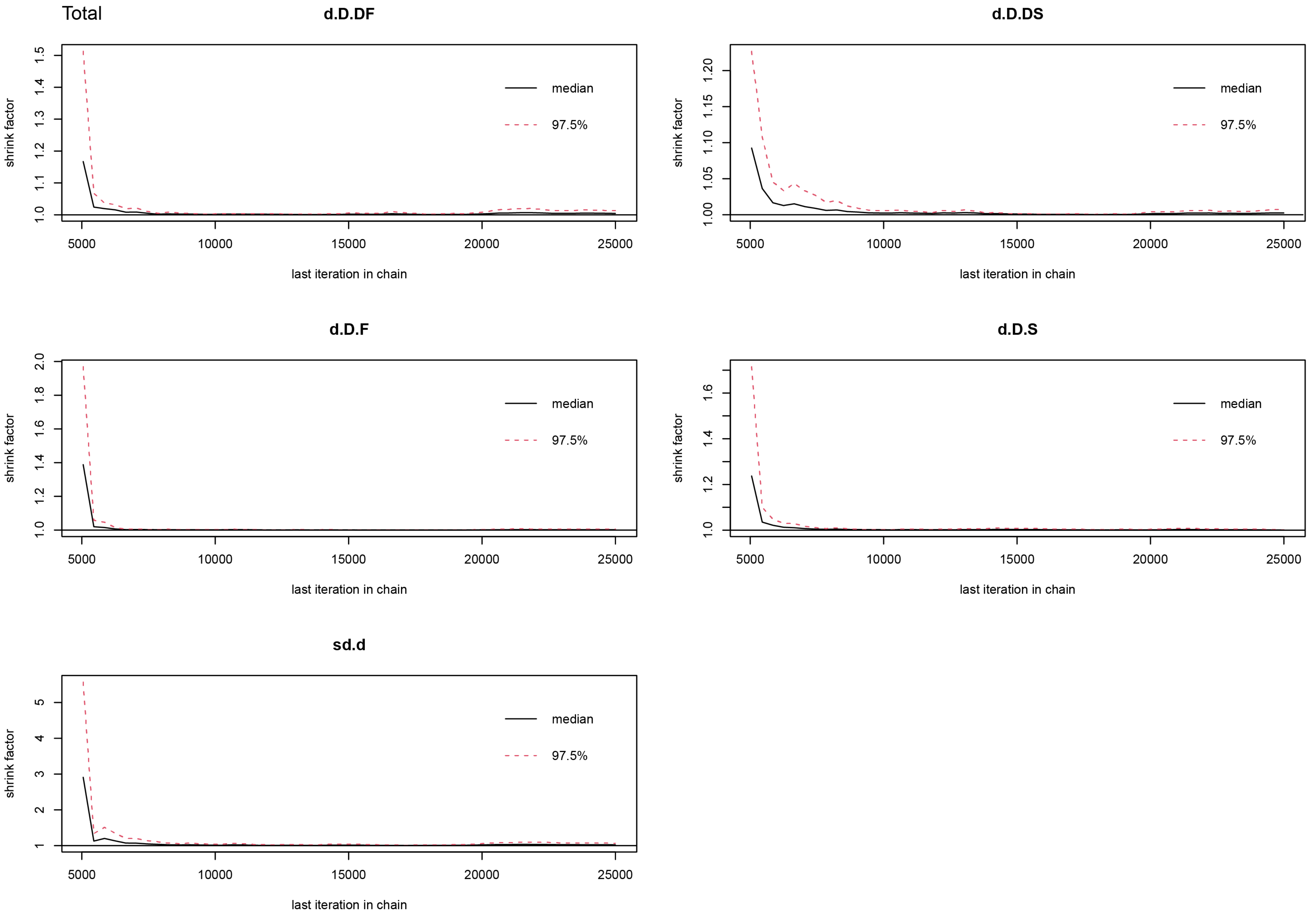

Total
d.D.DF
d.D.DS
d.D.F
d.D.S
sd.d
shrink factor
last iteration in chain
median
97.5%
5000
10000
15000
20000
25000

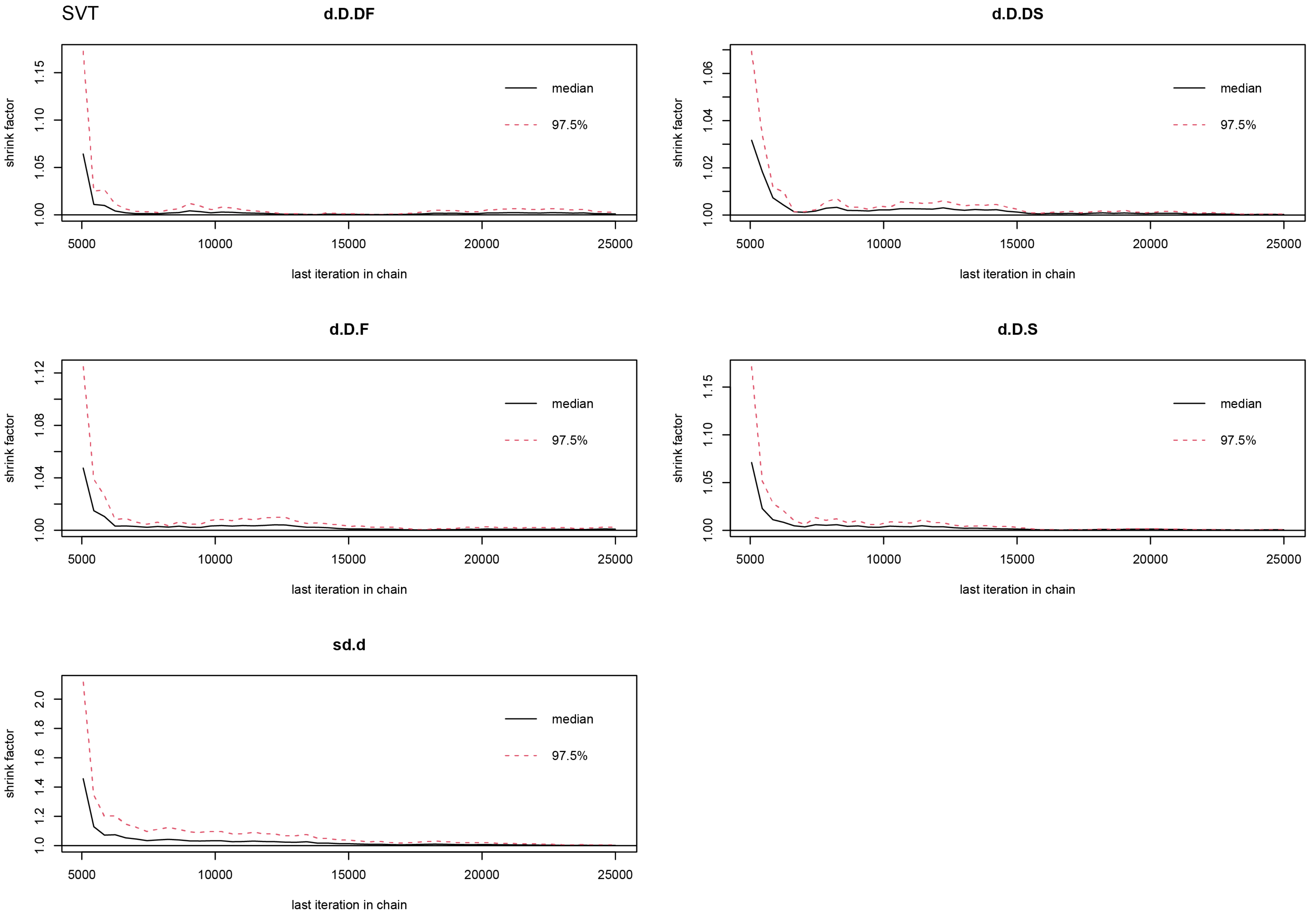
SVT
d.D.DF
d.D.DS
d.D.F
d.D.S
sd.d
shrink factor
last iteration in chain
median
97.5%
5000
10000
15000
20000
25000

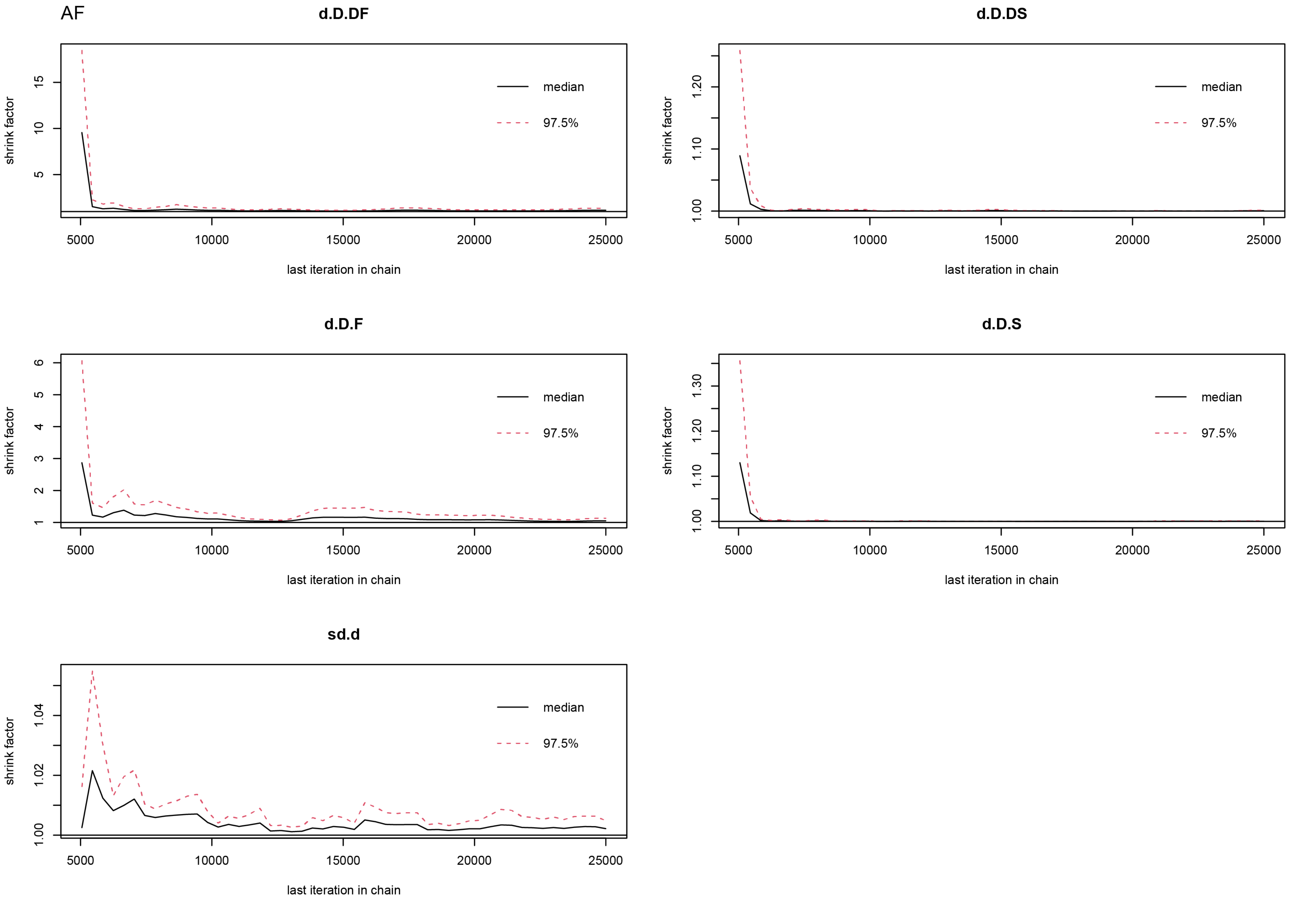
AF
d.D.DF
d.D.DS
d.D.F
d.D.S
sd.d
shrink factor
last iteration in chain
median
97.5%
5000
10000
15000
20000
25000
5
10
15
1.00
1.10
1.20
1.30
1
2
3
4
5
6
1.02
1.04

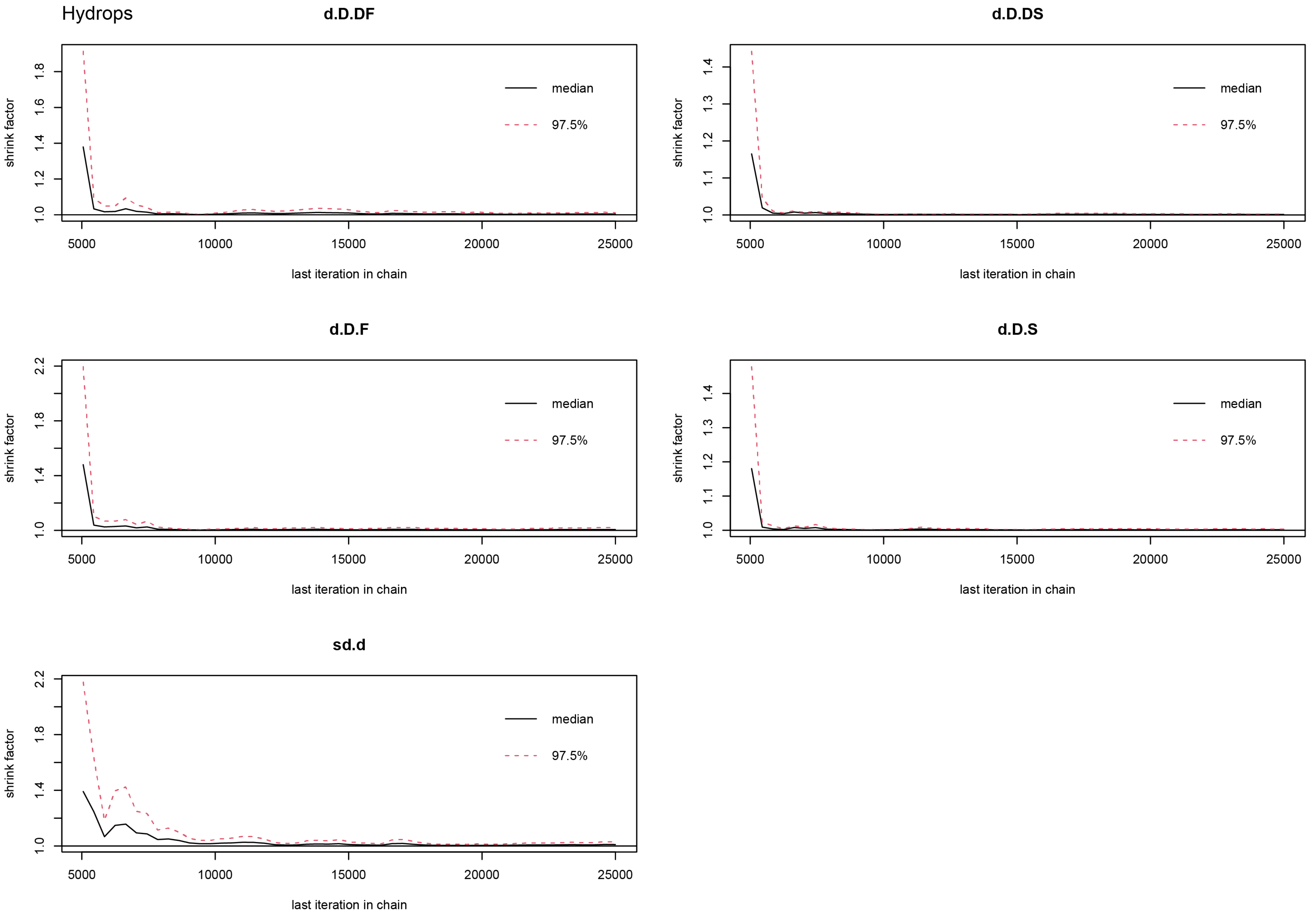
Hydrops
d.D.DF
d.D.DS
d.D.F
d.D.S
sd.d
shrink factor
last iteration in chain
median
97.5%
5000
10000
15000
20000
25000
1.0
1.1
1.2
1.3
1.4
1.6
1.8
2.2

Non-hydrops

d.D.DF

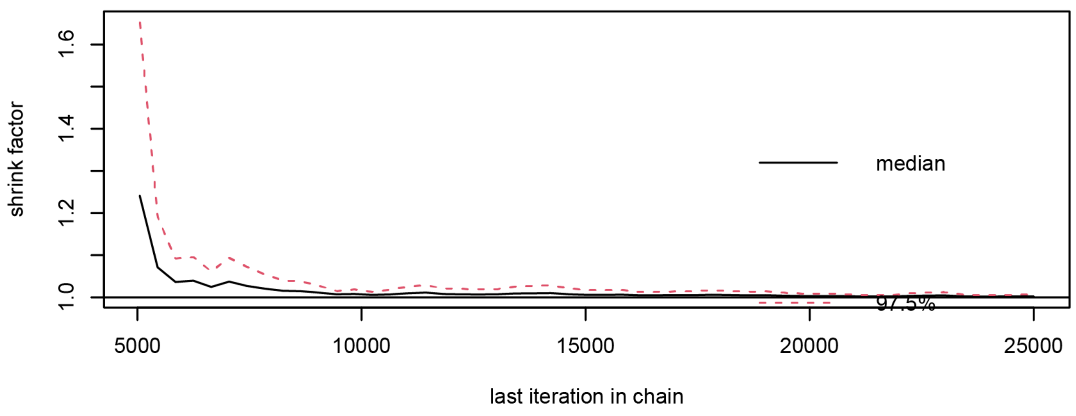

d.D.DS

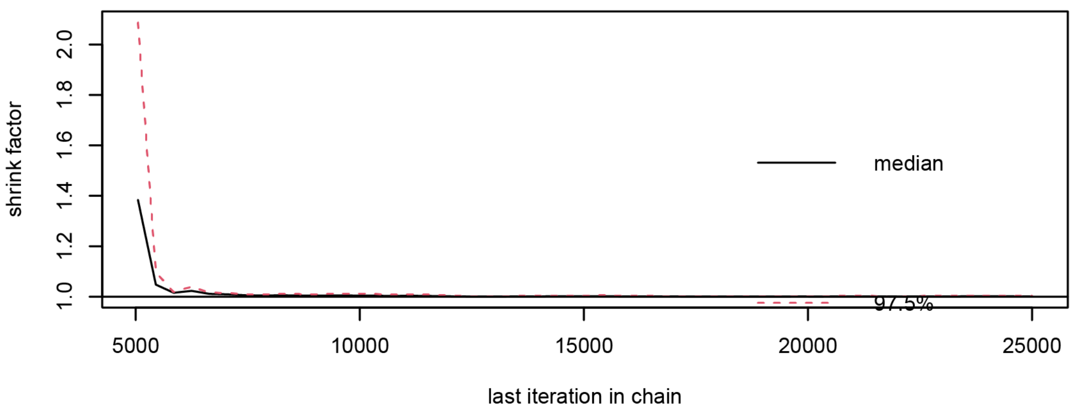

d.D.F

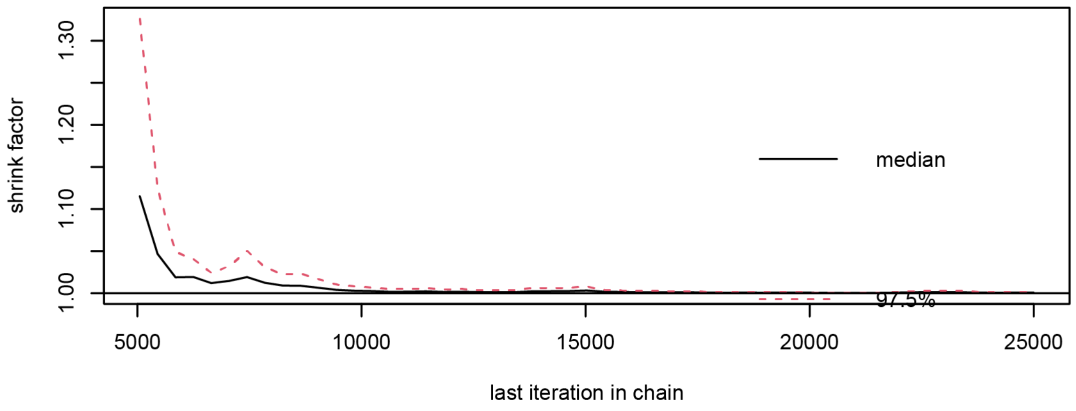

d.D.S

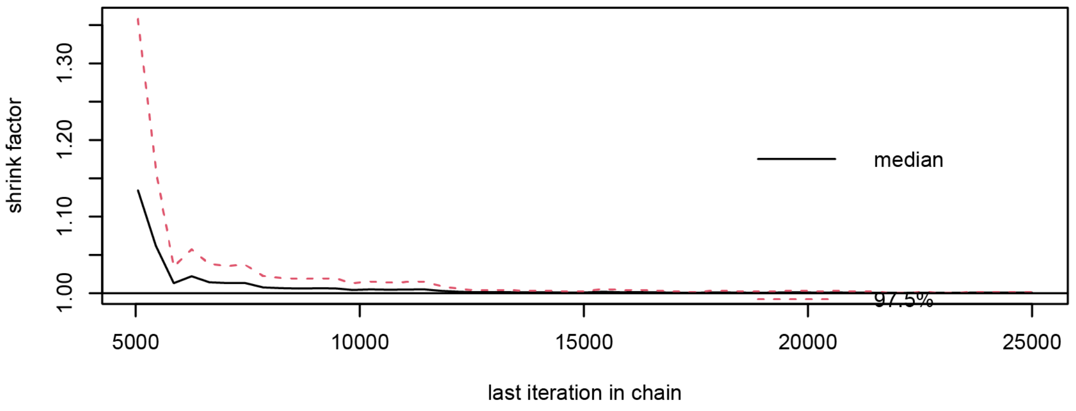

sd.d

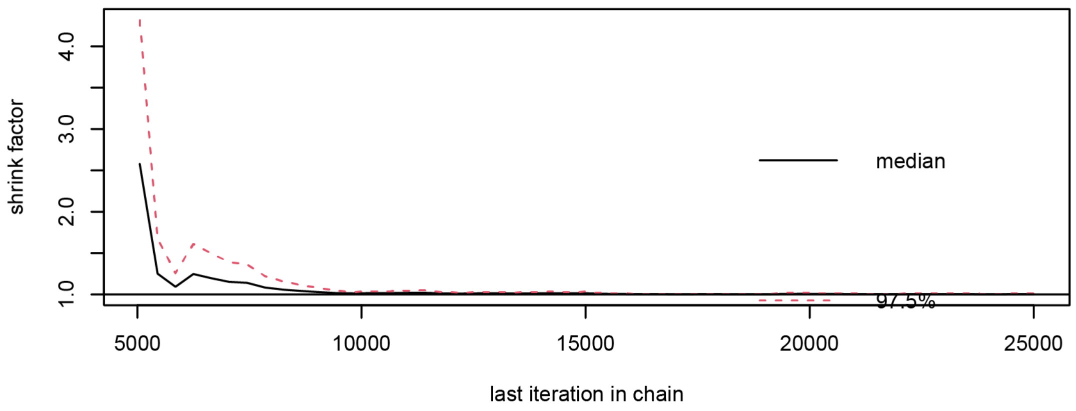

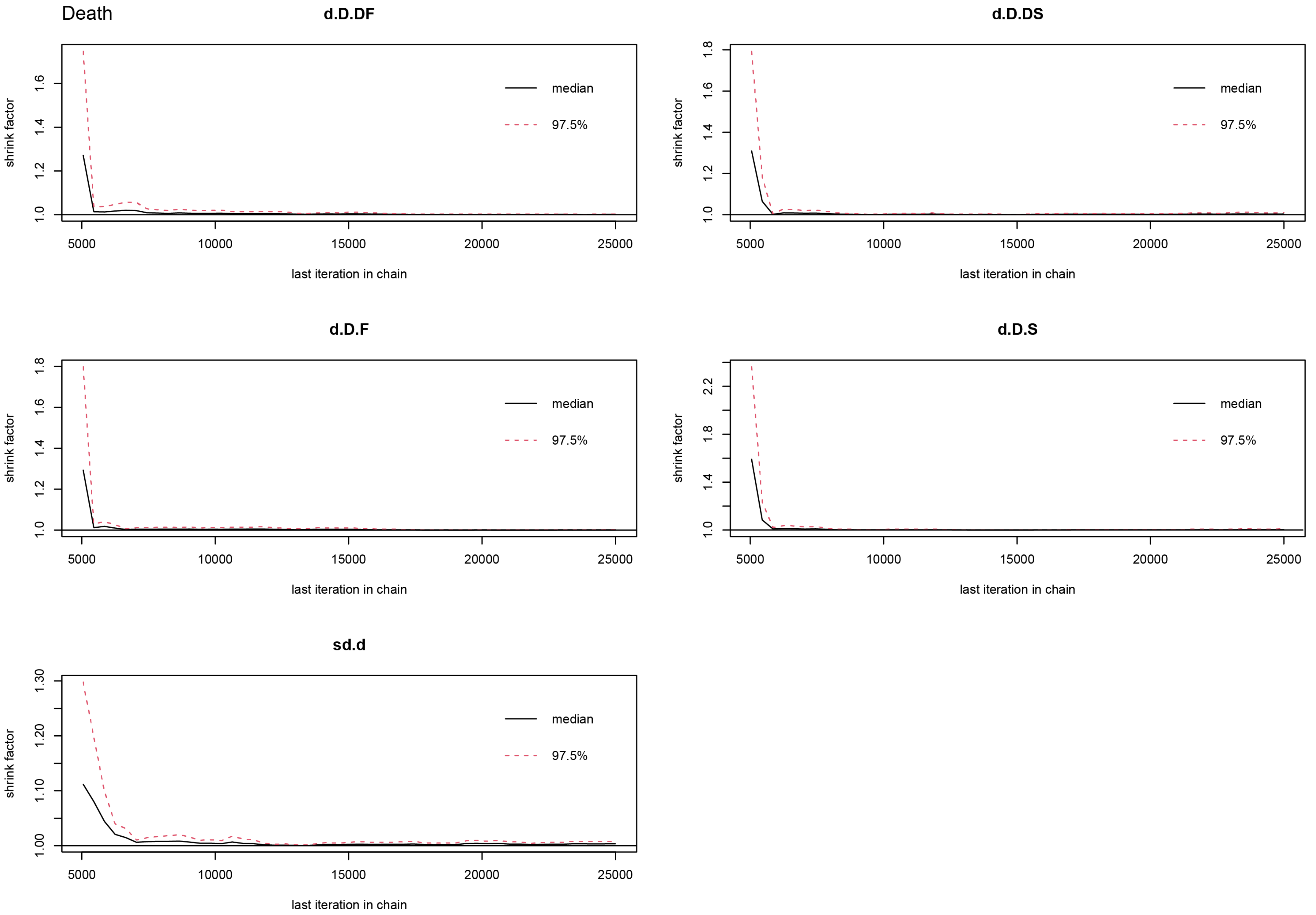
Death
d.D.DF
d.D.DS
d.D.F
d.D.S
sd.d
median
97.5%
shrink factor
last iteration in chain
5000
10000
15000
20000
25000

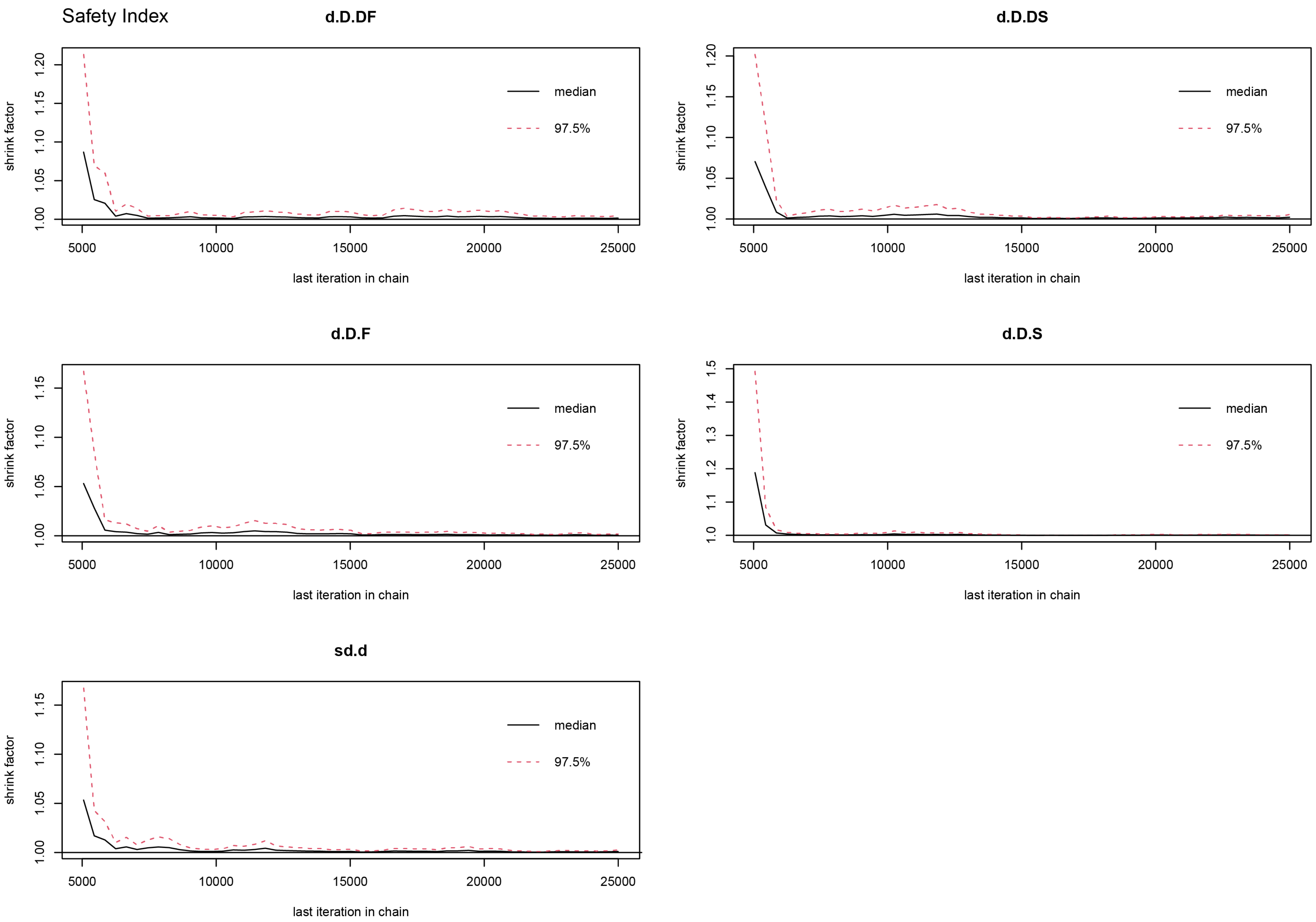
Safety Index
d.D.DF
d.D.DS
d.D.F
d.D.S
sd.d
shrink factor
last iteration in chain
median
97.5%
5000
10000
15000
20000
25000
1.00
1.05
1.10
1.15
1.20
1.0
1.1
1.2
1.3
1.4
1.5

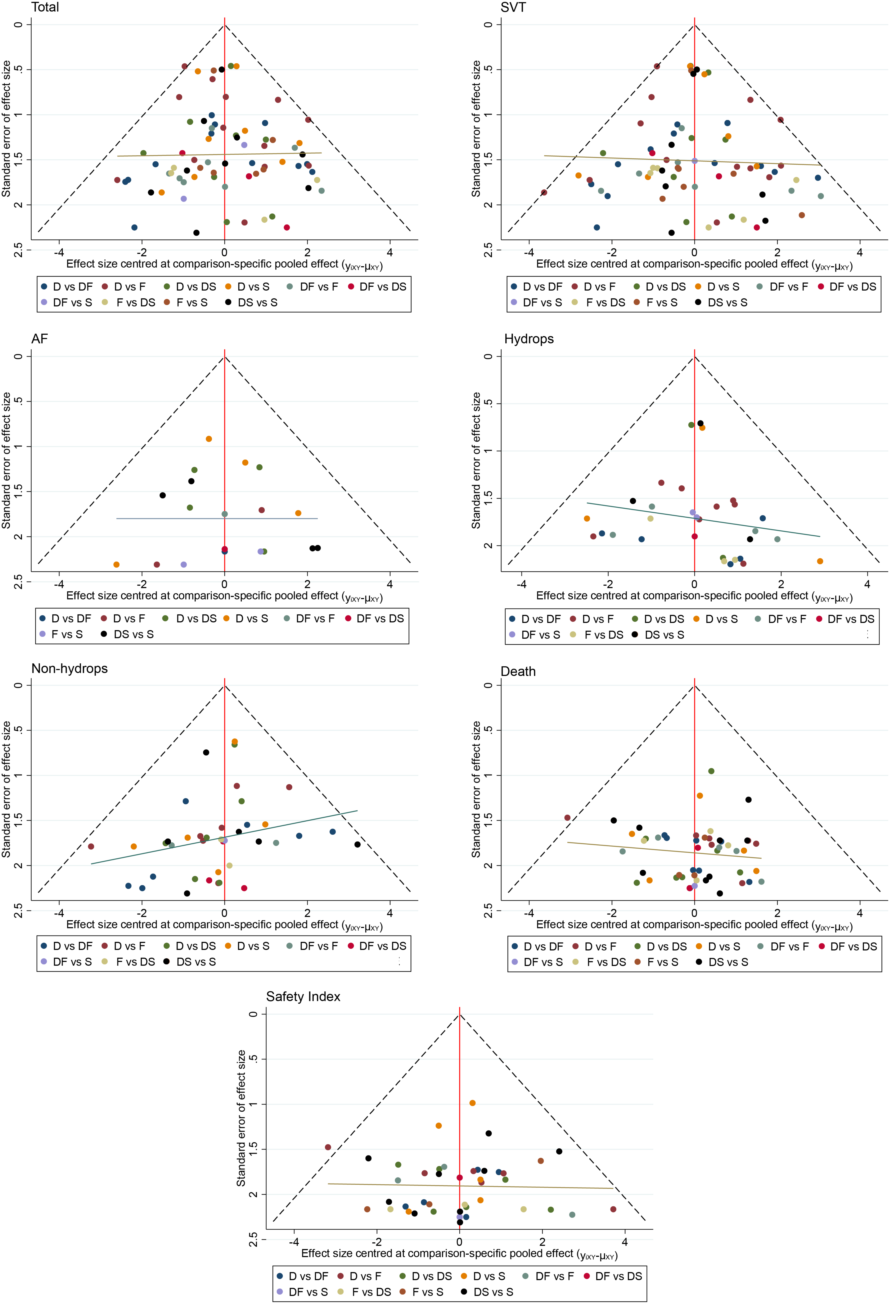

Total
SVT
AF
Hydrops
Non-hydrops
Death
Safety Index
Standard error of effect size
Effect size centred at comparison-specific pooled effect (y_{iXY}-μ_{XY})
0
.5
1
1.5
2
2.5
-4
-2
0
2
4
D vs DF
D vs F
D vs DS
D vs S
DF vs F
DF vs DS
DF vs S
F vs DS
F vs S
DS vs S
